# A novel small molecule inhibitor targeting KHSRP methylation suppresses colon cancer progression

**DOI:** 10.1016/j.apsb.2026.06.031

**Published:** 2026-06-18

**Authors:** Shuang Chen, Ningjing Zhang, Yuxiao Zhang, Jiebin Fang, Zhen Tang, Dashan Zhang, Xinyi Tang, Yalin Yu, Mengde Xia, Yan Yi, Wanjing Ding, Zhongjun Ma

**Affiliations:** a Hainan Institute, Zhejiang University, Sanya 572025, China; b Institute of Marine Biology and Pharmacology, Ocean College, Zhejiang University, Zhoushan 316021, China; c School of Life Sciences, Westlake University, Hangzhou 310000, China; d Center of Synthetic Biology and Integrated Bioengineering, Westlake University, Hangzhou 310000, China

**Keywords:** KHSRP, Arginine methylation, DNA damage, PRMT5, PLK1, ABPP, Colon cancer, Small molecule inhibitor

## Abstract

RNA-binding proteins (RBPs) are emerging as crucial regulators in cancer, but the development of therapeutic strategies targeting RBPs remains limited. Here, through chemical proteomics approaches, we identify DIQ01 as a novel small-molecule inhibitor of KH-type splicing regulatory protein (KHSRP), an RBP that is aberrantly hyper-expressed in human tumors and plays an essential role in proliferation, metastasis, and tumor progression. DIQ01 specifically binds to the KH3 and KH4 domains (Phe358 as the key residue) of KHSRP, inhibiting its interaction with mRNAs. Furthermore, the binding of DIQ01 diminishes the PRMT5-mediated arginine methylation of KHSRP, a post-translational modification required for its oncogenic activity. Mechanistically, transcriptomic and proteomic profiling suggested that DIQ01 functionally inactivates KHSRP, triggering destabilization and downregulation of its target *PLK1* mRNA. This leads to S-phase arrest, DNA damage, and apoptosis in HCT116 cells. Both *in vitro* and *in vivo* studies, including CRC xenograft and patient-derived organoid models, demonstrate that DIQ01 exhibits potent antitumor efficacy with minimal systemic toxicity. Our findings underscore the innovative approach of concurrently targeting the RNA-binding function of an RBP and its post-translational modification, highlighting DIQ01's unique mechanism and significant translational potential as a targeted cancer therapy.

## Graphical abstract

A chemical proteomics-guided screen identified DIQ01 as a novel KHSRP inhibitor that disrupts RNA binding and methylation, destabilizing *PLK1* mRNA and inducing DNA damage in colorectal cancer models.

**Figure:**
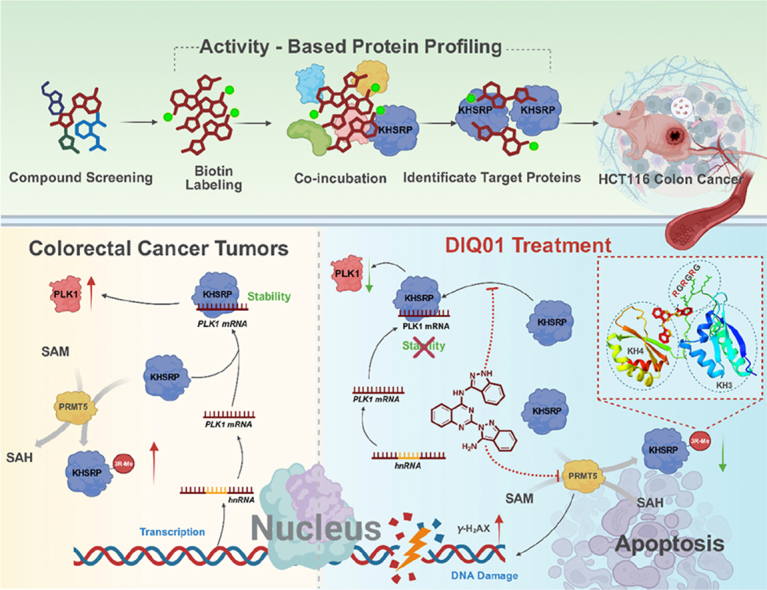

## 1 Introduction

Colorectal cancer (CRC) stands as a leading cause of cancer-related mortality globally. Despite significant advancements in chemotherapy and targeted therapies, such as 5-fluorouracil, oxaliplatin, regorafenib and EGFR inhibitors, the challenges of therapeutic resistance and tumor recurrence persist, particularly in advanced stages of the disease^1^, ^2^, ^3^, ^4^. This underscores the urgent need for innovative therapeutic targets that regulate CRC cell survival and genome maintenance^5^.

RBPs and their post-translational modifications have emerged as pivotal regulators of cancer biology, influencing nearly all hallmarks of cancer through their roles in mRNA splicing, stability, and translation^6^^,^^7^. In colorectal cancer, multiple RBPs (including LIN28, HuR, and IGF2BP family proteins) have been implicated in tumor growth, metastasis, and drug resistance^8^, ^9^, ^10^, ^11^, ^12^, ^13^, ^14^. Nevertheless, the majority of RBPs remain undruggable because of the noncatalytic and complex interactions between protein and RNA, which limit the discovery of specific inhibitors^15^.

KHSRP is a multifunctional RNA-binding protein that promotes the decay of AU-rich element (ARE)-containing mRNAs and regulates microRNA biogenesis^16^, ^17^, ^18^. Functionally, KHSRP has been implicated in promoting tumor cell proliferation, driving cell cycle progression, and facilitating adaptation to cellular stress^18^^,^^19^. It is frequently overexpressed in CRC and associated with poor prognosis^20^^,^^21^. Furthermore, studies have highlighted additional layers of regulation, such as SUMOylation of KHSRP by SUMO1, which prevents the biogenesis of TL-G-rich miRNAs, thereby promoting tumorigenesis^22^. ATM-mediated phosphorylation of KSRP plays an indispensable role in transmitting DNA damage signals to the miRNA processing machinery^23^. Acetylated KHSRP impairs DNA damage response and facilitates prostate cancer tumorigenesis^24^. Despite its critical role in cancer, pharmacological approaches to targeting KHSRP have been scarce. To date, only three small-molecule compounds have been reported to bind KHSRP^25^, ^26^, ^27^, yet their downstream functional consequences and *in vivo* efficacy remain unclear. The absence of potent and well-characterized KHSRP inhibitors has impeded our understanding of its therapeutic potential and regulatory mechanism in cancer.

In this study, we identify and characterize DIQ01, a marine-derived small molecule that binds to the KH3 and KH4 domains of KHSRP, with Phe358 identified as a key residue. DIQ01 disrupts its RNA-binding activity and subsequently suppresses its post-transcriptional regulation of oncogenic mRNAs. Mechanistically, DIQ01 reduces the expression of PLK1, a crucial mitotic kinase^28^, ^29^, ^30^, ^31^, regulated by KHSRP, and induces DNA damage in CRC cells. Surprisingly, we also discovered that DIQ01 decreases PRMT5-mediated arginine methylation of KHSRP, which is essential for its full oncogenic function. Our findings unveil a novel strategy for targeting KHSRP through dual interference with its RNA regulatory function and post-translational modification. Consequently, DIQ01 treatment leads to significant S-phase arrest, accumulation of DNA damage, and apoptosis in CRC cells. We establish that DIQ01 dualistically disables the oncogenic activity of KHSRP, thereby paving new pathways for therapeutic intervention in colorectal cancer.

## 2 Materials and methods

### 2.1 Chemicals and reagents

DIQ01 (C_22_H_16_N_8_, exact mass = 392.1498), DIQ02 (C_22_H_16_N_8_, exact mass = 392.1498), and Biotin-DIQ01 (C_41_H_47_N_11_O_6_S, exact mass = 821.3431) were synthesized in our laboratory and confirmed by ^1^H NMR, ^13^C NMR, MS analysis and X-ray diffraction techniques (Supporting Information). 5-Fluorouracil (MedChemExpress, Cat. No. HY-90006, Monmouth Junction, NJ, USA), regorafenib (MedChemExpress, Cat. No. HY-10331, Monmouth Junction, NJ, USA), GSK591 (MedChemExpress, Cat. No. HY-100235, Monmouth Junction, NJ, USA), EZM2302 (MedChemExpress, Cat. No. HY-111109, Monmouth Junction, NJ, USA), and puromycin dihydrochloride (MedChemExpress, Cat. No. HY-B1743A, Monmouth Junction, NJ, USA) were purchased from MedChemExpress. RIPA lysis buffer (Thermo Scientific, Cat. No. 89900, Waltham, MA, USA) and Halt protease and phosphatase inhibitor cocktail (Thermo Scientific, Cat. No. 78440, Waltham, MA, USA) were obtained from Thermo Scientific. Tween-20 (J&K Scientific/Bailingwei, Cat. No. 573169, Beijing, China) was sourced from Bailingwei. Beyozol reagent for total RNA extraction (Beyotime, Cat. No. R0011, Shanghai, China) was purchased from Beyotime. PVDF membranes (Merck Millipore, Immobilon-P, Cat. No. IPVH00010, Burlington, MA, USA) were obtained from Merck Millipore, and ECL chemiluminescent substrates (APExBIO, Cat. No. K1230, Houston, TX, USA) were obtained from APExBIO. Fetal bovine serum (Newzerum, Cat. No. FBS-CS500, Christchurch, New Zealand) was provided by Newzerum. 0.25% trypsin-EDTA digestion solution without phenol red (Servicebio, Cat. No. G4004, Wuhan, China) and McCoy's 5A medium (Servicebio, Cat. No. G4541, Wuhan, China) were purchased from Servicebio. The 6 × SDS loading buffer (TransGen Biotech, Cat. No. DL101-02, Beijing, China) was purchased from TransGen Biotech.

### 2.2 Cell lines and culture conditions

Human colorectal cancer HCT116, breast cancer MDA-MB-231, and non-small cell lung cancer A549 cell lines were purchased from the Type Culture Collection of the Chinese Academy of Sciences (Shanghai, China). All cell lines were cultured according to the supplier's instructions. Cells were routinely tested and confirmed to be free of mycoplasma contamination and used within 25 to 30 passages post-thaw.

### 2.3 Antibodies and plasmids

GAPDH (Proteintech, Cat. No. 10494-1-AP, Wuhan, China), Tubulin (Proteintech, Cat. No. 66031-1-Ig), *β*-actin (Proteintech, Cat. No. 20536-1-AP), c-Myc (Proteintech, Cat. No. 10828-1-AP), p53 (Proteintech, Cat. No. 60283-2-Ig), HA tag (Proteintech, Cat. No. 51064-2-AP), and *γ*-H2AX (Proteintech, Cat. No. 83307-2-RR) antibodies for Western blotting were obtained from Proteintech. CDK2 (Cell Signaling Technology, Cat. No. 2546T, Danvers, MA, USA) and CDK4 (Cell Signaling Technology, Cat. No. 12790T) antibodies for Western blotting were obtained from Cell Signaling Technology. KHSRP antibody for Western blotting and immunoprecipitation (ABclonal, Cat. No. A9075, Wuhan, China), pan-mono-methyl arginine antibody for Western blotting (ABclonal, Cat. No. A17984), and PRMT4 antibody for Western blotting (ABclonal, Cat. No. A21106) were obtained from ABclonal. FLAG (DYKDDDDK) tag antibody for Western blotting and immunoprecipitation (Smart-Lifesciences, Cat. No. SLAB0101, Changzhou, China) was obtained from Smart-Lifesciences. PLK1 antibody for Western blotting (MedChemExpress, Cat. No. HY-P80972, Monmouth Junction, NJ, USA) was obtained from MedChemExpress.

The FLAG–KHSRP expression plasmid was synthesized by Shhebio (Shanghai, China). Expression plasmids encoding HA-tagged PRMT4 and PRMT5 were purchased from Miaoling Biological Technology (Wuhan, China). FLAG-tagged full-length KHSRP mutants containing the Q349A, I356A, F358A, and K368A substitutions were synthesized by GenScript (Nanjing, China). Four truncated KHSRP variants, including the N-terminal region plus KH1-KH2 domains (KN, residues 1–305), KH1–KH2 domains (K12, residues 144–306), KH3–KH4 domains (K34, residues 321–520), and the C-terminal region (KC, residues 571–684), were generated by PCR amplification using full-length FLAG–KHSRP (1–711 aa) as the template. PCR products and the empty vector were digested with NheI-HF (New England Biolabs, Cat. No. R3131, Ipswich, MA, USA) and BamHI-HF (New England Biolabs, Cat. No. R3136), followed by ligation using T4 DNA ligase (TaKaRa, Cat. No. 2011A, Kusatsu, Shiga, Japan) at 4 °C overnight. The ligation products were transformed into competent DH5α Escherichia coli cells and subsequently plated onto LB agar containing 100 μg/mL ampicillin. Plates were incubated upside down at 37 °C overnight. Single bacterial colonies were picked and inoculated into 1 mL of liquid LB medium supplemented with ampicillin and cultured at 37 °C with shaking at 180 rpm in a thermostatic shaking incubator (Hualida, HZ-2410KB, Taicang, China) for 12–16 h. Cultures were preserved long-term by adding sterile 45% glycerol solution and storing at −80 °C. Plasmid DNA was purified using a FastPure EndoFree Plasmid Mini Plus Kit (Vazyme, Cat. No. DC204, Nanjing, China) according to the manufacturer's instructions.

### 2.4 Cytotoxicity assay

Cells were seeded into 96-well plates at a density of 5 × 10^3^ cells per well. After overnight adherence, cells were treated with various concentrations of test compounds and positive controls for 72 h, with each concentration assayed in triplicate. Following treatment, the culture medium was discarded, and cells were fixed with 100 μL of ice-cold 10% trichloroacetic acid at 4 °C overnight. After fixation, plates were thoroughly washed with flowing water at least four times and allowed to air dry. Cells were then stained with 0.4% sulforhodamine B (SRB) solution prepared in 1% acetic acid for 20 min at room temperature, followed by four washes with 1% acetic acid to remove excess dye. Plates were air-dried again, after which 100 μL of 10 mmol/L Tris base solution was added to each well to solubilize the protein-bound dye. Absorbance was measured at 515 nm using a multimode microplate reader (Tecan, Spark 10M, Männedorf, Switzerland). The IC_50_ values were determined using GraphPad Prism 8.0.1 software (GraphPad Software, San Diego, CA, USA).

### 2.5 Colony formation assay

The clonogenic potential of HCT116 cells was evaluated to determine the effects of DIQ01 treatment on cell proliferation and survival. Briefly, HCT116 cells (3 × 10^3^ cells per well) were seeded into six-well plates and allowed to adhere overnight. Cells were then treated with DIQ01 for 24 h, followed by incubation for an additional 7 days to allow colony formation. Colonies were subsequently fixed with 4% paraformaldehyde, stained with 0.5% crystal violet, and quantified by microscopic examination. Experiments were performed in triplicate, and results are presented as the mean ± standard deviation (SD) from three independent experiments.

### 2.6 Cell cycle analysis

HCT116 cells in logarithmic growth phase were seeded into six-well plates at a density of 5 × 10^5^ cells per well and incubated overnight in a humidified CO_2_ incubator (Thermo Scientific, Forma Series II 3111, Waltham, MA, USA) containing 5% CO_2_. The medium was then replaced with fresh medium containing the indicated concentrations of DIQ01 or vehicle control (DMSO) and incubated for an additional 24 h. Subsequently, cells were harvested by centrifugation at approximately 180×*g* for 10 min using a refrigerated centrifuge (Eppendorf, 5804 R, Hamburg, Germany), washed once with phosphate-buffered saline (PBS), and fixed overnight at −20 °C in 75% ethanol. After fixation, cells were pelleted by centrifugation at approximately 180×*g* for 5 min, washed twice with PBS to remove residual ethanol, and resuspended in Hank's balanced salt solution supplemented with RNase A (50 μg/mL) and propidium iodide (50 μg/mL). Cells were incubated at room temperature in the dark for 30 min before analysis. Cell cycle distribution was measured using a Gallios flow cytometer (Beckman Coulter, Gallios, Brea, CA, USA).

### 2.7 Wound-healing assay

The wound-healing assay was conducted to evaluate the inhibitory effects of compound DIQ01 on cell migration. Cells were seeded into 24-well plates at a density of 8 × 10^4^ cells per well and incubated overnight at 37 °C in a humidified incubator (Thermo Scientific) with 5% CO_2_ to achieve confluency. A scratch wound was subsequently created in the confluent cell monolayer using a sterile 20 μL pipette tip. Cells were then gently washed with phosphate-buffered saline (PBS) to remove detached cells and debris, followed by treatment with DIQ01 or vehicle control in fresh culture medium. After incubation for 24 and 48 h, images of wound closure were captured using an inverted fluorescence microscope (Leica Microsystems, DMi8, Wetzlar, Germany). All experiments were performed independently at least three times.

### 2.8 Western blot analysis

HCT116 cells were cultured in six-well plates and treated with the indicated concentrations of compounds, followed by incubation at 37 °C under standard culture conditions. Post-treatment, cells were washed briefly with ice-cold PBS and collected into microcentrifuge tubes. Cells were lysed in RIPA lysis buffer (Thermo Scientific) supplemented with 1 × protease inhibitor cocktail (Thermo Scientific), incubated on ice for 30 min, and centrifuged at 13,000×*g* for 20 min at 4 °C using a refrigerated centrifuge (Eppendorf). Protein concentrations were quantified using a DC Protein Assay Kit (Bio-Rad, Cat. No. 5000111, Hercules, CA, USA) according to the manufacturer's instructions. The lysates were mixed with 6 × Protein Loading Buffer (TransGen Biotech, Cat. No. DL101-02, Beijing, China) and denatured by heating at 95 °C for 10 min. Equal amounts of proteins were separated by SDS-polyacrylamide gel electrophoresis (SDS-PAGE) and transferred onto polyvinylidene difluoride (PVDF) membranes (0.45 μm; Millipore). Membranes were subsequently blocked in 5% non-fat milk in TBST (Tris-buffered saline with 0.1% Tween-20) at room temperature for 1 h and incubated overnight at 4 °C with appropriate primary antibodies. After washing three times with TBST, membranes were incubated with corresponding secondary antibodies at room temperature for 1 h. Following additional washes, protein signals were detected using an Odyssey infrared imaging system (LI-COR Biosciences, Lincoln, NE, USA).

### 2.9 Alkaline comet assay

HCT116 cells were seeded into six-well plates at a density of 2 × 10^5^ cells per well and incubated overnight to allow cell attachment. Following adhesion, cells were treated with the indicated concentrations of compounds for 12 h. Cells were subsequently harvested, washed gently with phosphate-buffered saline (PBS), and resuspended in 150 μL PBS. DNA damage was assessed using an alkaline comet assay kit (Beyotime, Cat. No. C2041S, Shanghai, China) according to the manufacturer's instructions. Briefly, the assay was independently repeated three times, and comet images were captured and analyzed using an inverted fluorescence microscope (Leica Microsystems).

### 2.10 Pull-down assay

The Smart Streptavidin Magnetic IP/Co-IP Kit (Smart-Lifesciences, Cat. No. SM017K02, Changzhou, China) was used for pull-down experiments. HCT116 cells were lysed using the lysis/wash buffer supplied with the kit, followed by protein quantification. Whole-cell protein extracts containing 1 mg of total protein were incubated with biotin, Biotin-DIQ01, or Biotin-DIQ01 together with excess unlabeled DIQ01 at 4 °C overnight. Streptavidin magnetic beads were then added to capture biotin-associated protein complexes according to the manufacturer's instructions. The bead-bound proteins were eluted and separated by SDS-PAGE. For target identification, gels were visualized using a Silver Stain Kit (Beyotime, Cat. No. P0017S, Shanghai, China), and gel bands containing the target proteins were carefully excised, followed by in-gel digestion and mass spectrometry-based protein identification performed by Beijing Biotech Pack Scientific Co., Ltd. (Beijing, China). For validation experiments, the eluted proteins were analyzed by Western blotting.

### 2.11 Proteome microarray assay

HuProt™ v2.0 Human Proteome Microarrays (CDI Laboratories, Baltimore, MD, USA) were utilized to identify proteins interacting with DIQ01. Microarrays were equilibrated to room temperature and incubated with blocking buffer (3% bovine serum albumin in phosphate-buffered saline) for 1 h. Biotin-DIQ01 (10 μmol/L) was then incubated with the microarrays for 1 h in reaction buffer (1% BSA in PBS). After washing with PBS three times for 5 min each, Cy3-conjugated streptavidin (diluted 1000-fold in sample incubation solution) was applied and incubated for 1 h (light exposure was avoided). Subsequent washing steps included PBS and ultrapure water rinses for 5 min each. The interactions between Biotin-DIQ01 and proteins were detected at 635 nm using a GenePix 4200A microarray scanner (Axon Instruments/Molecular Devices, GenePix 4200A, Union City, CA, USA). Data analysis was performed using GenePix Prospector software 6.0 (Axon Instruments, Union City, CA, USA), calculating the signal-to-noise ratio (SNR) as the median foreground signal divided by the median background signal. SNR values were averaged from duplicate spots on each microarray. SNR values with and without Biotin-DIQ01 were denoted as SNR (+) and SNR (−), respectively. The *Z*-score ratio was defined as SNR (Biotin-DIQ01)/SNR (Biotin).

### 2.12 Cellular thermal shift assay

HCT116 cells were treated with DIQ01 (10 μmol/L) or DMSO for 3 h. Cells were then aliquoted and heated at different temperatures ranging from 56 to 68 °C for 5 min using a thermal cycler (Bio-Rad, T100, Hercules, CA, USA). After cooling to room temperature, cells were lysed by five freeze-thaw cycles in liquid nitrogen. Soluble proteins were collected by centrifugation at 12,000×*g* for 15 min at 4 °C using a refrigerated centrifuge (Eppendorf), and then detected by Western blotting.

### 2.13 Protein expression and purification

All plasmid construction and protein expression were performed by Zoonbio Co., Ltd. (Nanjing, China). Full-length human KHSRP (residues 1–711) was cloned into the EcoRI/BamHI sites of the pcDNA3.4 vector. The recombinant plasmid was transfected into HEK293 cells, followed by supplementation with nitrogen sources and growth factors 24 h post-transfection. On Day 6, cell viability and morphology were assessed, and cells were harvested and lysed. The fusion protein was purified using Ni–IDA affinity chromatography, and its purity was verified by Coomassie Brilliant Blue (CBB) staining and Western blot analysis.

For expression of truncated domains, KH3 (residues 317–418) and KH4 (residues 423–525) were seamlessly cloned into the pSUMO-Mut expression vector. The resulting constructs, pSUMO-Mut-KH3 and pSUMO-Mut-KH4, were transformed into ArcticExpress competent cells and induced with 0.2 mmol/L IPTG at 37 °C for 4 h with shaking. After SUMO protease cleavage, proteins were sequentially purified by Ni affinity chromatography, Q ion-exchange chromatography, and size-exclusion chromatography. Protein purity was confirmed by CBB staining.

### 2.14 Differential scanning fluorimetry (DSF)

Recombinant proteins were diluted in DSF assay buffer consisting of 25 mmol/L HEPES-NaOH (pH 7.5), 300 mmol/L NaCl, 10% glycerol, 0.04% Triton X-100, and 0.5 mmol/L TCEP. Thermal shift assays were performed using a LightCycler® 480 System II (Roche Diagnostics, LightCycler 480 System II, Mannheim, Germany), with SYPRO Orange (Sigma–Aldrich, Cat. No. S5692, St. Louis, MO, USA) as the fluorescent probe. The dye binds to hydrophobic regions exposed upon protein unfolding, enabling real-time monitoring of thermal denaturation. Each 20 μL reaction contained 5 μmol/L protein, 5 × SYPRO Orange, and varying concentrations of DIQ01. The fluorescence signal was recorded at an excitation wavelength of 465 nm and an emission wavelength of 580 nm. The temperature was increased from 25 to 90 °C at a ramp rate of 1 °C min^−1^. The measured fluorescence was plotted as a function of temperature, and the melting temperature was calculated from the inflection point of the resulting sigmoidal fluorescence curve using GraphPad Prism software version 8.0.1 (GraphPad Software)^32^^,^^33^.

### 2.15 MST binding assay and nano-DSF

Microscale thermophoresis (MST) and nano-DSF analyses were performed by Readcrystal Biotechnology Co., Ltd. (Suzhou, China) to evaluate the interaction between KHSRP and the small molecule DIQ01. MST assays were conducted using a Monolith® NT.115 instrument (NanoTemper Technologies, Monolith NT.115, Munich, Germany) with standard treated capillaries. All measurements were performed in PBS containing 1% DMSO. Prior to analysis, all samples were centrifuged to remove potential aggregates. Ligands were diluted in target protein at twice the final concentration. MST was performed under high MST power with 100% LED intensity. Data were analyzed using MO. AffinityAnalysis software (NanoTemper Technologies).

Nano-DSF experiments were carried out in triplicate using a Prometheus NT.48 instrument (NanoTemper Technologies, Prometheus NT.48, Munich, Germany). Protein samples (0.5 mg/mL in PBS containing 1% DMSO) were loaded into manufacturer-recommended capillaries. Thermal unfolding was monitored by measuring the fluorescence ratio (*F*_350_/*F*_330_) during a linear temperature ramp from 25 to 95 °C at a rate of 1.8 °C/min, with excitation at 280 nm. Data analysis was performed using PR. ThermControl software (NanoTemper Technologies).

### 2.16 Quantitative real-time PCR analysis

Total RNA was extracted using Beyozol reagent (Beyotime) following the manufacturer's protocol. cDNA was synthesized from 1 μg of total RNA using the HiScript II 1st Strand cDNA Synthesis Kit (+gDNA wiper) (Vazyme, Cat. No. R212-02, Nanjing, China). Quantitative real-time PCR was performed using ChamQ Universal SYBR qPCR Master Mix (Vazyme, Cat. No. Q711-03, Nanjing, China) on a LightCycler® 480 System II (Roche Diagnostics). Relative mRNA expression levels were normalized to *GAPDH* and calculated using the 2^–ΔΔCt^ method.

### 2.17 RNA-seq and bioinformatics analysis

HCT116 cells were treated with DIQ01 (500 nmol/L) or vehicle control (DMSO) for 24 h, with three biological replicates per group. Total RNA was extracted using TRIzol™ reagent (Invitrogen, Cat. No. 15596026, Carlsbad, CA, USA) according to the manufacturer's instructions, and RNA samples were immediately snap-frozen in liquid nitrogen. RNA sequencing and preliminary data processing were performed by LC-Bio Technology Co., Ltd. (Hangzhou, China). Bioinformatic analyses were conducted using OmicStudio tools (LC-Bio Technology Co., Ltd., Hangzhou, China) (https://www.omicstudio.cn/tool).

### 2.18 Fluorescence polarization (FP) assay

5′-FAM-labeled RNA probes were synthesized by GenScript (Nanjing, China) with the following sequences: AU probe, 5′-FAM-UAUUUAUUAUUUAUUUAUUAUUUAU-3′; and unlabeled cold probe, 5′-UAUUUAUUAUUUAUUUAUUAUUUAU-3′^34^. Initial optimization experiments were performed in 384-well black plates (Corning, Cat. No. 3575, Corning, NY, USA) using a SYNERGY H1 multimode microplate reader (BioTek Instruments, Synergy H1, Winooski, VT, USA). For assay optimization, KHSRP or KH3 proteins were incubated with the AU probe in assay buffer (25 mmol/L Tris, pH 7.5, 25 mmol/L NaCl, 1% (*v*/*v*) Tween-20) at a final volume of 30 μL for 30 min at room temperature, followed by fluorescence polarization (FP) measurements. Based on the optimization results, KHSRP (75 nmol/L) with AU probe (10 nmol/L), and KH3 (2 μmol/L) with AU probe (60 nmol/L) were used for subsequent DIQ01 activity assays^35^.

### 2.19 Electrophoretic mobility shift assay (EMSA)

DIQ01 was pre-incubated with 1 μmol/L KHSRP for 10 min at room temperature, followed by the addition of 1 μmol/L 5′-FAM-labeled RNA probe in assay buffer (25 mmol/L Tris, pH 7.5, 25 mmol/L NaCl, 1% (*v*/*v*) Tween-20). Samples were subjected to non-denaturing polyacrylamide gel electrophoresis, and shifts in RNA–protein complexes were visualized using a G:BOX imaging system (Syngene, G:BOX, Cambridge, UK)^35^.

### 2.20 H&E staining and immunohistochemistry

Mouse tumors and tissues were fixed in formalin, paraffin-embedded, and sectioned at a thickness of 5 μm. For histological analysis, sections were deparaffinized in xylene for 2 × 15 min, rehydrated through a graded ethanol series, and rinsed in tap water. Sections were stained with Harris hematoxylin for 5 min, differentiated in 1% acid alcohol for 30 s, rinsed, counterstained with eosin for 5 min, dehydrated through ethanol and xylene, and mounted with neutral resin. Slides were examined microscopically. For immunohistochemistry and TUNEL staining, sections were processed according to the manufacturer's protocol by Servicebio (Wuhan, China). Sections were stained for TUNEL, cleaved caspase-3, and Ki-67. Images were captured using an inverted fluorescence microscope (Leica Microsystems).

### 2.21 Transient transfection

Small interfering RNAs (siRNAs) targeting human *KHSRP* were designed and synthesized by GenePharma (Shanghai, China). Transfection was performed using jetPRIME® transfection reagent (Polyplus-transfection, Cat. No. 101000027, Illkirch, France) according to the manufacturer's instructions. HCT116 cells were seeded at approximately 40% confluence, transfected the following day, and the medium was replaced after 6 h. Cells were harvested 48 h post-transfection for downstream analysis.

Plasmid transfection was performed using Lipofectamine™ 3000 transfection reagent (Invitrogen, Cat. No. L3000015, Carlsbad, CA, USA) according to the manufacturer's instructions. HCT116 or HEK293T cells were seeded to reach approximately 70% confluence the next day. Cells were harvested 48 h post-transfection for subsequent experiments.

### 2.22 Lentiviral construction of stable KHSRP knockdown HCT116 cell line

HCT116 cells in the logarithmic growth phase were seeded into 24-well plates at a density of 2 × 10^5^ cells per well in 500 μL of culture medium. The cells were incubated at 37 °C in a humidified CO_2_ incubator (Thermo Scientific) containing 5% CO_2_, and lentiviral infection was performed when cell confluence reached 30%–50%. Prior to infection, lentiviral particles were slowly thawed on ice, and the required viral volume per well was calculated based on a multiplicity of infection (MOI) of 20 and a viral titer of 1 × 10^8^ TU/mL. The original medium was removed, and 250 μL of fresh complete medium was added to each well. The calculated volume of lentiviral stock was then added and gently mixed with 5 μg/mL Polybrene (hexadimethrine bromide; Sigma–Aldrich, Cat. No. H9268, St. Louis, MO, USA) to enhance infection efficiency. After 4 h of incubation with the lentivirus, fresh complete medium was added to each well. Approximately 24 h post-infection, the virus-containing medium was replaced with an equal volume of fresh complete medium, and the cells were returned to the CO_2_ incubator (Thermo Scientific) for continued culture. After 48 h of infection, red fluorescent protein (RFP) expression was observed under a fluorescence microscope (Leica Microsystems) to assess infection efficiency. When the infection efficiency reached at least 80% and cell confluence was 60%–70%, fresh complete medium containing 4 μg/mL puromycin (MedChemExpress) was added to select stable transduced cell lines.

### 2.23 Proteomic sequencing

Proteomic profiling was conducted by Novogene Co., Ltd. (Beijing, China). HCT116 cells were treated with DIQ01 (500 nmol/L) or DMSO for 24 h, with three biological replicates per group. Cells were lysed in DB buffer containing 8 mol/L urea and 100 mmol/L triethylammonium bicarbonate (TEAB, pH 8.5), sonicated on ice, and centrifuged at 12,000×*g* for 15 min at 4 °C. Supernatants were reduced with 1 mol/L dithiothreitol (DTT) at 56 °C for 1 h, alkylated with iodoacetamide at room temperature in the dark for 1 h, and quenched on ice. Samples were diluted to 100 μL, digested with trypsin at 37 °C for 4 h and then overnight, acidified to pH < 3, and centrifuged. Peptides were desalted using C18 columns, washed with 0.1% formic acid in 3% acetonitrile, eluted with 70% acetonitrile, and lyophilized. LC–MS/MS analysis was performed in data-independent acquisition (DIA) mode, with an m/z range of 380–980, 300 variable windows, and a resolution of 240,000. Protein identification, quantification, and preliminary bioinformatic analyses were performed using the standard DIA proteomics workflow provided by Novogene Co., Ltd. Differentially expressed proteins (DEPs) were functionally annotated using public databases, including Gene Ontology (GO), InterPro (IPR), Kyoto Encyclopedia of Genes and Genomes (KEGG), and Clusters of Orthologous Groups (COG).

### 2.24 Immunoprecipitation analysis

Cells were washed with ice-cold PBS and lysed in IP lysis buffer consisting of 25 mmol/L Tris-HCl (pH 8.0), 150 mmol/L NaCl, 10% glycerol, and 1% IGEPAL CA-360, supplemented with a protease inhibitor cocktail (Thermo Scientific), for 30 min on ice. Cell lysates were centrifuged at 13,000×*g* for 20 min at 4 °C using a refrigerated centrifuge (Eppendorf). Protein concentrations were quantified using a DC Protein Assay Kit (Bio-Rad) according to the manufacturer's instructions. Equal amounts of lysates (1–5 mg) were incubated overnight at 4 °C with antibodies against KHSRP, FLAG, HA, or control IgG. Protein A/G magnetic beads (MedChemExpress, Cat. No. HY-K0202, Monmouth Junction, NJ, USA) were then added and incubated at room temperature for 1 h, followed by washing with wash buffer consisting of PBS and 0.5% Tween-20 (pH 7.4). The immunoprecipitates were analyzed by immunoblotting using the indicated primary antibodies.

### 2.25 RNA immunoprecipitation (RIP)

A total of 1 × 10^7^ HCT116 cells were seeded and processed using a PureBinding® RNA Immunoprecipitation Kit (Geneseed, Cat. No. P0101, Guangzhou, China) according to the manufacturer's instructions. Cell extracts were prepared using the lysis buffer supplied with the kit and incubated with 2 μg of anti-KHSRP antibody or normal human IgG control at 4 °C for 4 h. RNA–protein immunocomplexes were captured using protein A/G beads supplied with the kit, and RNA was subsequently purified from the complexes for RT-qPCR analysis.

### 2.26 Colorectal cancer organoid establishment and IC_50_ assay

CRC specimens were provided by Hangzhou Bio-Sincerity Pharma-Tech Co., Ltd. (Hangzhou, China) under approval No. CR-SR-24012. Human CRC organoid culture and compound sensitivity assays were performed by Hangzhou Bio-Sincerity Pharma-Tech Co., Ltd. according to its standard operating procedures. Briefly, fresh tumor samples were collected after surgical resection, transported to the laboratory at 4 °C, washed with pre-chilled DPBS, minced into small pieces, and enzymatically digested to generate single-cell suspensions. After filtration through a 40 μm cell strainer and centrifugation at 800×*g* for 5 min, the resulting cell pellet was resuspended in Matrigel and seeded into 24-well culture plates. After Matrigel solidification for 30 min, organoid culture medium was added. The culture medium was prepared according to the protocol described by Miyoshi and Stappenbeck (2013). For IC_50_ determination, organoids were treated with varying concentrations of the test compound for 72 h. Cell viability was assessed using a CellTiter-Glo-based luminescent viability assay according to the service provider's standard protocol^36^.

### 2.27 Actinomycin D assay

HCT116 cells were seeded into six-well plates at a density of 5 × 10^5^ cells per well and incubated overnight at 37 °C. Cells were pretreated with DIQ01 (1 μmol/L) or DMSO for 30 min, followed by treatment with actinomycin D (MedChemExpress, Cat. No. HY-17559, Monmouth Junction, NJ, USA) at 10 μg/mL to inhibit transcription. Total RNA was collected at 0, 2, 4, 6, and 8 h after actinomycin D addition. Quantitative RT-PCR was performed to analyze the transcript levels of *PLK1* and *GAPDH*. Transcript levels were normalized to the corresponding levels at *t* = 0, defined as the time of actinomycin D addition.

### 2.28 Cycloheximide (CHX) chase assay

HCT116 cells were seeded into six-well plates at a density of 5 × 10^5^ cells per well and incubated overnight at 37 °C in a humidified CO_2_ incubator (Thermo Scientific) containing 5% CO_2_ to allow cell attachment. The following day, cells were pretreated with DIQ01 (500 nmol/L) or DMSO for 30 min, followed by the addition of cycloheximide (CHX; MedChemExpress, Cat. No. HY-12320, Monmouth Junction, NJ, USA) at a final concentration of 100 μg/mL to inhibit protein synthesis. Cells were collected at 0, 2, 4, 8, 16, and 24 h after CHX addition, lysed, and subjected to Western blotting to detect KHSRP, PLK1, and GAPDH.

### 2.29 Mouse PK study

The pharmacokinetic study of DIQ01 was performed by Hunan Hengxing Medical Technology Co., Ltd. (Changsha, China). The pharmacokinetics of DIQ01 were assessed in male BALB/c mice (*n* = 3) following intravenous (i.v.) administration at 3 mg/kg or oral (*p.o.*) administration at 25 mg/kg. DIQ01 was formulated in a vehicle consisting of ethanol/Cremophor EL/PBS (1:1:8, *v*/*v*/*v*). Blood samples (0.1 mL) were collected through a catheter at various time points (0.08, 0.25, 0.5, 1, 2, 4, 6, 8, and 24 h) after DIQ01 administration. Blood samples were treated with K_2_-EDTA and kept on ice. Within 1 h of collection, the blood samples were centrifuged at 6800×*g* for 6 min at 4 °C, and the resulting plasma was transferred to polypropylene tubes and stored at −80 °C. Plasma concentrations of DIQ01 were determined using a validated LC–MS/MS method. For analysis, a 20 μL aliquot of plasma was subjected to protein precipitation with 200 μL of acetonitrile containing 100 ng/mL internal standard (IS). The mixture was vortexed for 1 min and centrifuged at 18,000×*g* for 7 min. Then, 150 μL of the supernatant was transferred to a 96-well plate and mixed with 150 μL of water. Finally, 1 μL of the resulting solution was injected for LC–MS/MS analysis. Pharmacokinetic parameters were calculated by noncompartmental analysis using Phoenix WinNonlin software version 8.3 based on the mean plasma concentration-time profiles.

### 2.30 Human colorectal tumor xenograft efficacy study *in vivo*

All animal experiments were approved by the Animal Ethics Committee of Zhejiang Ocean University (approval No. 2024002) and conducted in accordance with institutional guidelines for the care and use of laboratory animals. Male BALB/c nude mice aged 4–6 weeks and weighing 18–22 g were purchased from Hangzhou Qizhen Experimental Animal Technology Co., Ltd. (Hangzhou, China) and acclimated for approximately one week before experimentation. Human colorectal cancer HCT116 cells were cultured in McCoy's 5A medium and subcutaneously injected into the right flank of each mouse at 3 × 10^6^ cells per mouse. Once the average tumor volume reached approximately 80 mm^3^, mice were randomized into treatment groups (*n* = 9 per group), and treatment was initiated on Day 0 for a total duration of two weeks. DIQ01, 5-Fu, and regorafenib were dissolved in a vehicle consisting of ethanol/Cremophor EL/PBS (1:1:8, *v*/*v*/*v*). Intratumoral injections were administered to treatment groups 2–3 times per week at the designated doses, whereas control mice received an equivalent volume of vehicle. Tumor size and body weight were measured every two days using a digital caliper and an electronic balance, respectively. Tumor volume was calculated using Eq. (1):

(1) Tumor volume (mm^3^) = 1/2 × Length × Width^2^

After 14 days of treatment, mice were euthanized by cervical dislocation. Tumor xenografts were excised and weighed. Major organs, including the heart, liver, spleen, lungs, and kidneys, were collected for histological analysis by hematoxylin and eosin (H&E) staining.

The DIQ01 dose of 6 mg/kg was selected based on its *in vitro* potency (IC_50_ = 65 nmol/L) and mouse pharmacokinetic data. This dose achieved a free maximum plasma concentration (*C*_max,u_) of 0.56–2.23 μmol/L, which was approximately 8–33-fold higher than the IC_50_, and maintained exposure above the effective level for 10–16 h. These exposure levels support effective target engagement *in vivo*.

### 2.31 Limited proteolysis (LiP) followed by SDS-PAGE silver staining

Limited proteolysis of recombinant full-length KHSRP was performed with sequencing-grade clostripain (Arg-C; Promega, Cat. No. V1881, Madison, WI, USA) or sequencing-grade modified trypsin (Promega, Cat. No. V5113), followed by SDS-PAGE and silver staining using a Fast Silver Stain Kit (Beyotime, Cat. No. P0017S, Shanghai, China). Each 25 μL reaction contained approximately 1.875 μg of recombinant full-length KHSRP. KHSRP was pre-incubated with DIQ01 (0.4–10 μmol/L; DMSO-matched vehicle control) for 10 min at 37 °C in either Arg-C LiP buffer consisting of 50 mmol/L Tris–HCl (pH 7.6–7.9), 5 mmol/L CaCl_2_, 2 mmol/L EDTA, and freshly added 2.5 mmol/L DTT, or trypsin LiP buffer consisting of 50 mmol/L Tris-HCl (pH 8.0). Reactions were initiated by adding Arg-C (0.05 μg) or trypsin (0.025 μg), incubated at 37 °C for 10–90 min, quenched with 2 × SDS loading buffer, boiled at 95 °C for 5 min, resolved by SDS-PAGE, and visualized by silver staining. Each experiment was performed independently at least three times (Supporting Information Tables S1–S4).

### 2.32 *In vitro* PRMT5-mediated methylation of KHSRP

*In vitro* methylation reactions were assembled in a final volume of 30 μL containing 50 mmol/L Tris–HCl (pH 8.6), 0.02% Triton X-100, 2 mmol/L MgCl_2_, 2 mmol/L freshly prepared TCEP, 100 μmol/L freshly prepared *S*-adenosyl-l-methionine (SAM), and recombinant full-length KHSRP (2 μg). DIQ01 (0.4–10 μmol/L; DMSO-matched vehicle control) and, where indicated, the PRMT5 inhibitor GSK591 (10–100 nmol/L) were included during a 10 min pre-incubation at 37 °C. Reactions were initiated by adding recombinant PRMT5 (IVDSHOW, Cat. No. 31393, Beijing, China) at 0, 0.3, or 0.6 μg and incubated at 37 °C for 2–16 h. Reactions were quenched with 2 × SDS loading buffer, heated at 95 °C for 5 min, and 15 μL of each reaction was analyzed by SDS-PAGE and Western blotting with an anti-MMA antibody to detect KHSRP arginine methylation. A KHSRP-only reaction served as a negative control for substrate-derived MMA signal, and DMSO was matched across all conditions. Each experiment was performed independently at least three times.

### 2.33 Acute oral toxicity study

An acute oral toxicity limit test of DIQ01 was conducted in specific pathogen-free (SPF) BALB/c mice of both sexes, aged 6–9 weeks and weighing approximately 20 g. Mice were supplied by Hangzhou Qizhen Experimental Animal Technology Co., Ltd. (Hangzhou, China) and randomly assigned to four dose groups: 0, 75, 150, and 300 mg/kg, with three males and three females in each group. DIQ01 was administered once by oral gavage at the corresponding dose. After administration, all animals were closely monitored for clinical signs at 15, 30, 60, and 120 min and then daily for 14 days. Body weight was recorded daily throughout the observation period. At the end of the 14-day observation period, all animals were euthanized, and major organs, including the heart, liver, spleen, lungs, and kidneys, were collected for histopathological examination.

### 2.34 Statistical analyses

Data are presented as the mean ± SD and were analyzed using GraphPad Prism software version 8.0.1 (GraphPad Software). Statistical analyses included an unpaired two-tailed Student's *t*-test for comparisons between two groups and one-way analysis of variance (ANOVA) followed by Bonferroni's *post hoc* test for comparisons among multiple groups. A significance threshold of *P* < 0.05 was applied to determine statistical significance.

## 3 Results

### 3.1 Discovery of marine-derived DIQ01 as a potent inhibitor of tumor cell growth

**Figure 1:**
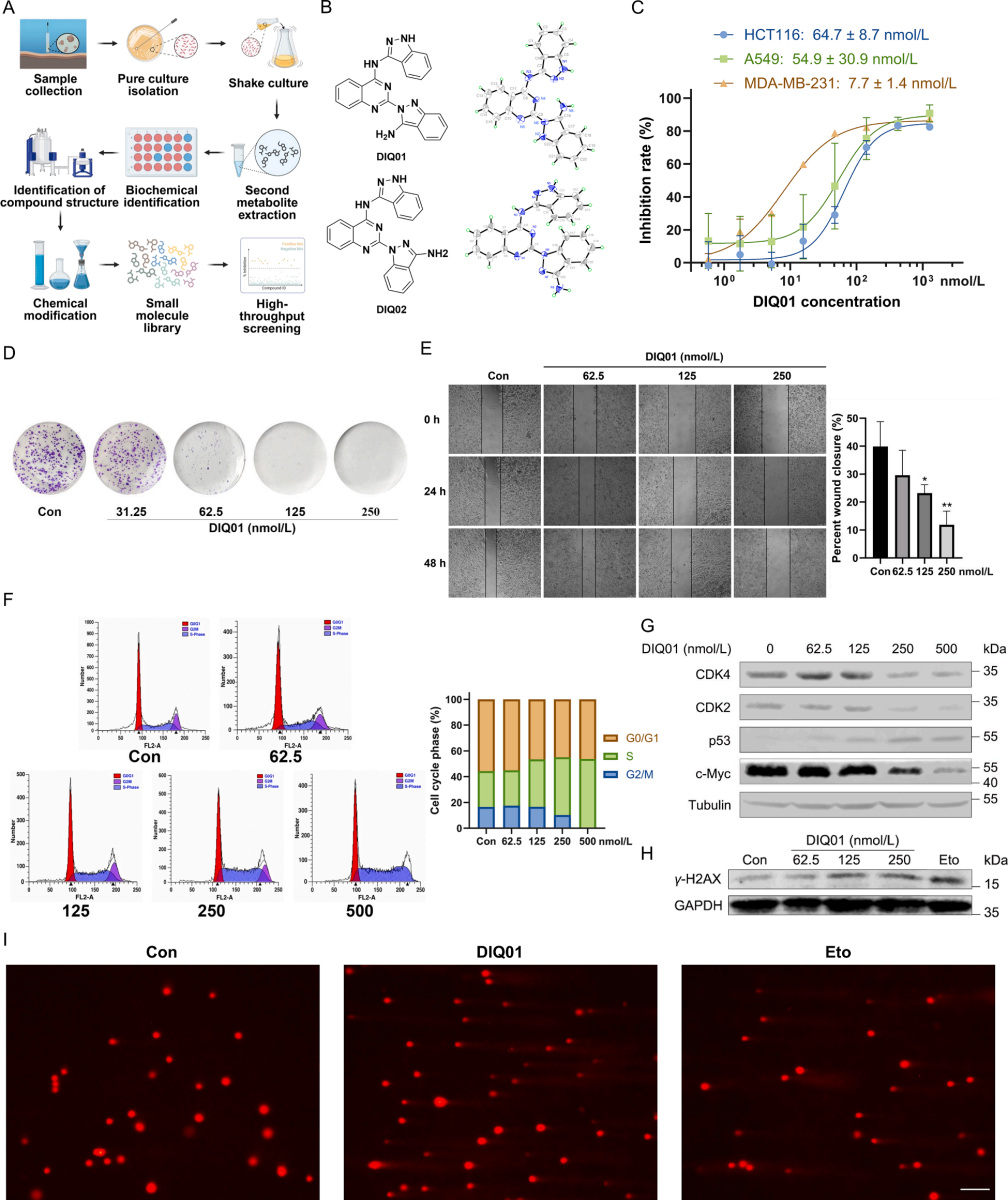
Identification and characterization of DIQ01, an antitumor agent derived from marine natural products. (A) Workflow depicting high-throughput screening of a small-molecule library from marine natural products. (B) Chemical and X-ray crystallographic structures of DIQ01 (CCDC 2449697) and its analog DIQ02 (CCDC 2449670). (C) Dose-response curves demonstrating antiproliferative effects of DIQ01 against three human cancer cell lines (HCT116, A549, and MDA-MB-231), assessed by the sulforhodamine B (SRB) assay after 72 h of treatment (mean ± SD, *n* = 3). (D) Representative colony formation assays of HCT116 cells treated with varying concentrations of DIQ01 for seven days, visualized by crystal violet staining. (E) Wound-healing assays assessing migration capacity of HCT116 cells treated with DIQ01 at increasing concentrations over 24 and 48 h intervals; cells treated with DMSO served as a negative control. Scale bar = 25 μm. Statistical significance: ∗*P* < 0.05, ∗∗*P* < 0.01, compared to control (mean ± SD, *n* = 3). (F) Flow cytometric analysis showing the effects of DIQ01 at gradient concentrations on the cell cycle progression of HCT116 cells after 24 h treatment. DMSO served as a vehicle control. (G) Western blot analysis revealing the expression levels of cell-cycle and proliferation-related proteins (p53, c-Myc, CDK4, and CDK2) in HCT116 cells after 24 h exposure to DIQ01, using Tubulin as a loading control. (H) Immunoblot detection of *γ*-H2AX expression as an indicator of DNA damage in HCT116 cells following 24 h treatment with DIQ01, with GAPDH as loading control and etoposide (Eto, 10 μmol/L) included as a positive control. (I) Alkaline comet assay illustrating DNA damage induced by DIQ01 (250 nmol/L) in HCT116 cells after 12 h treatment, with DMSO as negative control and Eto (10 μmol/L) as positive control. Scale bar = 5 μm.

Initially, we employed a high-throughput screening strategy to identify a series of bioactive natural small molecules from the fermentation products of marine actinobacteria. Their chemical structures were precisely elucidated using nuclear magnetic resonance (NMR), mass spectrometry (MS), and X-ray diffraction (XRD) techniques. To further enhance pharmacological efficacy and drug-likeness, we chemically modified the core molecular scaffold and successfully constructed a diverse small molecule library comprising over 2000 compounds (Fig. 1A)^37^, ^38^, ^39^, ^40^, ^41^. Subsequently, a systematic antitumor activity assessment revealed a pair of structurally unique isomeric compounds. These compounds consist of two 3-aminoindazoles linked to a quinazoline at the 2,4 positions, with a key structural difference in the position of nitrogen within the C–N bond between one of the 3-aminoindazoles and the quinazoline ring (Fig. 1B). We observed that DIQ01 and DIQ02 showed significant cytotoxicity irrespective of cancer type (Fig. 1C and Supporting Information Fig. S1A).

Given that DIQ01 exhibits superior activity against three distinct cancer cell lines, and considering the rising incidence of early-onset colorectal cancer, we selected DIQ01 for further investigation of its antitumor mechanism in the HCT116 colorectal cancer model. DIQ01 showed an IC_50_ value of 64.7 ± 8.7 nmol/L (Fig. 1C), exhibiting nearly two orders of magnitude greater potency than the standard chemotherapeutic agent 5-fluorouracil (5-Fu) (IC_50_: 4.3 ± 0.2 μmol/L) and the multikinase inhibitor regorafenib (Reg) (3.4 ± 0.1 μmol/L) (Fig. S1B). Plate colony formation assays also validated the effective anti-proliferative action of DIQ01 (Fig. 1D). Besides, wound healing assays demonstrated a concentration-dependent inhibition of tumor cell migration by DIQ01 (Fig. 1E). Altogether, these results indicated that DIQ01 exhibited strong antitumor activity *in vitro*. DIQ01 induced S-phase arrest in HCT116 colorectal cancer cells (Fig. 1F). Molecular analysis through Western blotting revealed that DIQ01 treatment significantly decreased the expression levels of key cell cycle and proliferation-regulatory proteins, such as CDK2, CDK4, and c-Myc, while upregulating p53 in a dose-dependent manner (Fig. 1G). The regulation of these proteins may indicate mechanisms of DNA damage. Specifically, p53 mediates cell cycle arrest in response to DNA damage^42^, while the inhibition of CDK2/CDK4 enforces G1/S phase arrest to facilitate repair^43^^,^^44^. Additionally, the loss of c-Myc can exacerbate DNA damage induced by replication stress^45^^,^^46^. Subsequently, Western blot analysis showed that DIQ01 treatment markedly increased levels of *γ*-H2AX, a hallmark of DNA double-strand breaks (Fig. 1H). Consistently, an alkaline comet assay demonstrated extensive DNA fragmentation in DIQ01-treated cells compared to control (Fig. 1I). Together, these findings suggest that the antitumor effects of DIQ01 are mediated by replication stress, which leads to irreparable DNA damage in cancer cells.

### 3.2 Identification of KHSRP as a direct target of DIQ01

**Figure 2:**
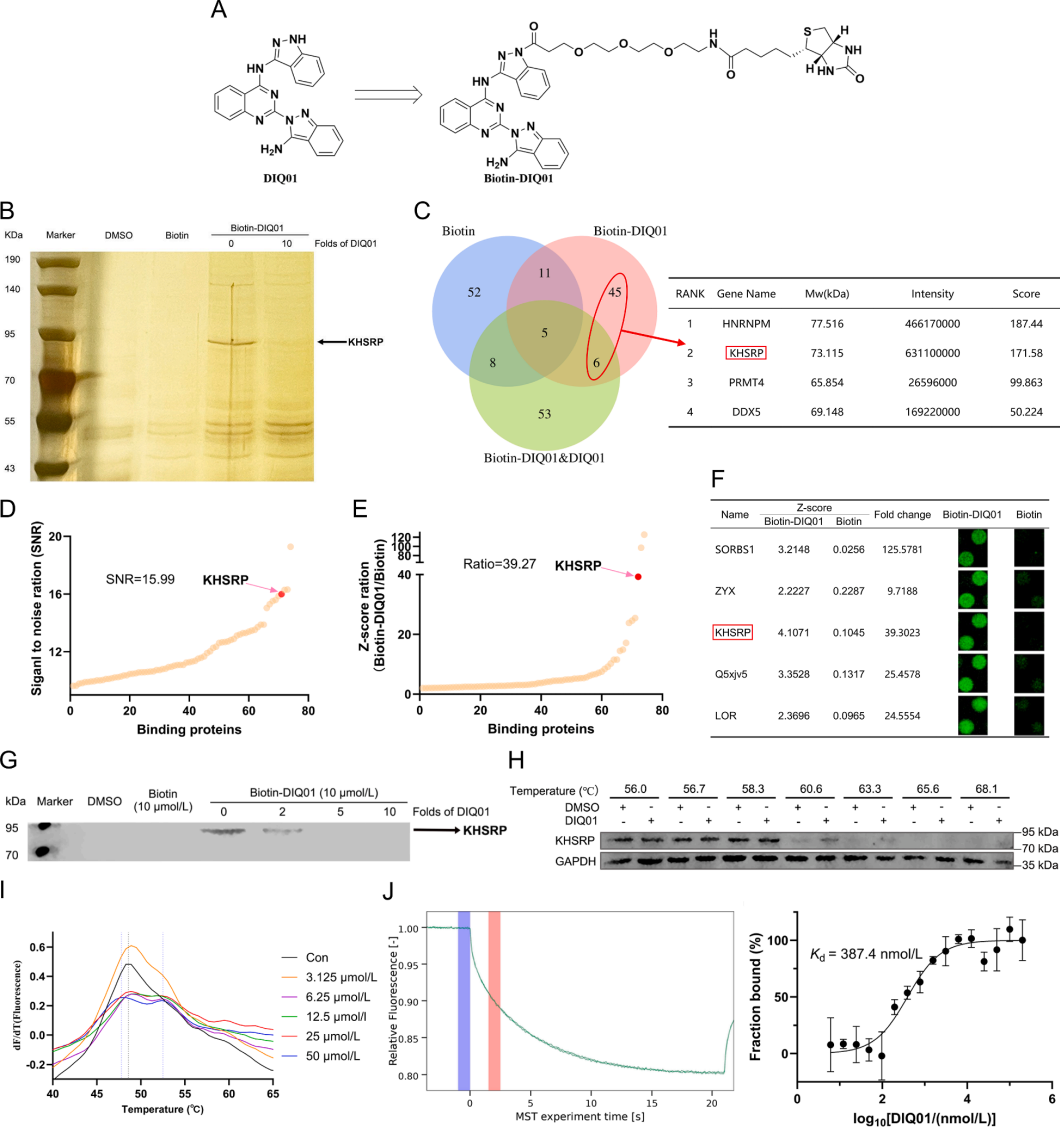
Identification of KHSRP as a direct cellular target of DIQ01. (A) Chemical structure of Biotin-DIQ01. (B) Biotin-DIQ01 specifically pulled down a protein band ranging between 70 and 95 kDa. HCT116 cell lysates were incubated overnight with Biotin-DIQ01 alone or with unlabeled DIQ01 as a competitor, followed by precipitation with streptavidin magnetic beads. Bound proteins were separated *via* gel electrophoresis and visualized using silver staining. The arrow indicated specific protein bands of interest. (C) Venn diagram illustrating protein overlaps identified by mass spectrometry (MS) among the Biotin control group, Biotin-DIQ01 group, and Biotin-DIQ01 plus DIQ01 competition group. Four candidate proteins were identified based on intensity and scoring analysis. (D) Proteome microarray identified KHSRP as a direct cellular target of DIQ01 using SNR analysis. (E) Proteome microarray identified KHSRP as a direct cellular target of DIQ01 using *Z*-score ratio analysis. (F) Combined analysis of SNR and *Z*-score ratio identified five potential protein targets. (G) Western blot analysis to determine the competitive binding of the Biotin-DIQ01 and DIQ01 to KHSRP. (H) CETSA assays confirmed the binding of DIQ01 to KHSRP in HCT116 cells, and GAPDH was used as the internal control. (I) DSF assay showed the interaction of DIQ01 with KHSRP. (J) MST analysis was used to assess the binding affinity between DIQ01 and KHSRP. Data are presented as the mean ± SD, *n* = 3.

The unique structural features and significant bioactivity of DIQ01 prompted us to further investigate its molecular target. To this end, we conducted a systematic analysis using activity-based protein profiling (ABPP), HuProt™ proteome microarray screening, and kinase profiling to identify potential binding targets of DIQ01. First, we synthesized a biotinylated DIQ01 probe (Biotin-DIQ01) to facilitate target identification (Fig. 2A), which retained comparable anticancer activity to DIQ01 (Supporting Information Fig. S2A). We then performed a pull-down assay in HCT116 cell lysates, followed by SDS-PAGE and silver staining. A distinct protein band of 70–95 kDa was enriched by Biotin-DIQ01, which was absent with a biotin-only control and was eliminated by competition with excess free DIQ01 (Fig. 2B). MS analysis identified the four most enriched candidate proteins (Fig. 2C). Importantly, unbiased screening *via* a HuProt™ proteome microarray (Fig. S2B) independently validated KHSRP as the sole overlapping candidate, highlighting it as the prime candidate for DIQ01's direct target. Notably, KHSRP emerged as a top hit on the microarray, showing a high signal-to-noise ratio (SNR) and *Z*-score among the candidates (Fig. 2D–F). Moreover, although the quinazoline-indazole scaffold of DIQ01 is reminiscent of many kinase inhibitors^47^^,^^48^, a broad kinase profiling panel revealed no significant inhibition of common kinases at 200 nmol/L (Fig. S2C). At this stage, multiple independent lines support KHSRP as the primary cellular effector of DIQ01, although minor contributions from additional targets cannot be entirely excluded.

We then thoroughly investigated the interaction between DIQ01 and KHSRP using a series of complementary assays. Streptavidin pull-down followed by Western blot confirmed that Biotin-DIQ01 specifically binds KHSRP from cell lysates, and this binding was competitively displaced by excess free DIQ01 (Fig. 2G). Cellular thermal shift assay (CETSA) demonstrated that DIQ01 treatment stabilizes endogenous KHSRP against heat-induced denaturation in intact cells (Fig. 2H); notably, the DIQ01 analog DIQ02 produced a similar stabilization of KHSRP in CETSA (Fig. S2D). For direct biophysical confirmation, full-length recombinant KHSRP was expressed and purified (Fig. S2E) to enable *in vitro* binding studies. Differential scanning fluorimetry (DSF) revealed that DIQ01 binding induces a pronounced conformational change in KHSRP. While the free KHSRP protein displayed a single thermal unfolding transition, the DIQ01-bound KHSRP showed a biphasic melting curve with two distinct inflection points (Fig. 2I). This shift from a single to dual melting peak suggested that DIQ01 binding may perturb the conformation of KHSRP, potentially by engaging one or more of its four KH domains (interconnected by flexible loops, Fig. S2F) and thereby differentially altering their stability^32^^,^^49^. Finally, we performed microscale thermophoresis (MST) analysis, which determined the binding affinity (*K*_d_) of DIQ01 to KHSRP as 387.4 nmol/L (Fig. 2J). Together, these results established KHSRP as the direct cellular target of DIQ01 and suggested that ligand-induced conformational changes in KHSRP may underlie the mechanism of action of DIQ01.

### 3.3 DIQ01 targets the KH3 domain of KHSRP and disrupts RNA binding

#### 3.3.1 Phe358 as the key residue for DIQ01-KHSRP binding

**Figure 3:**
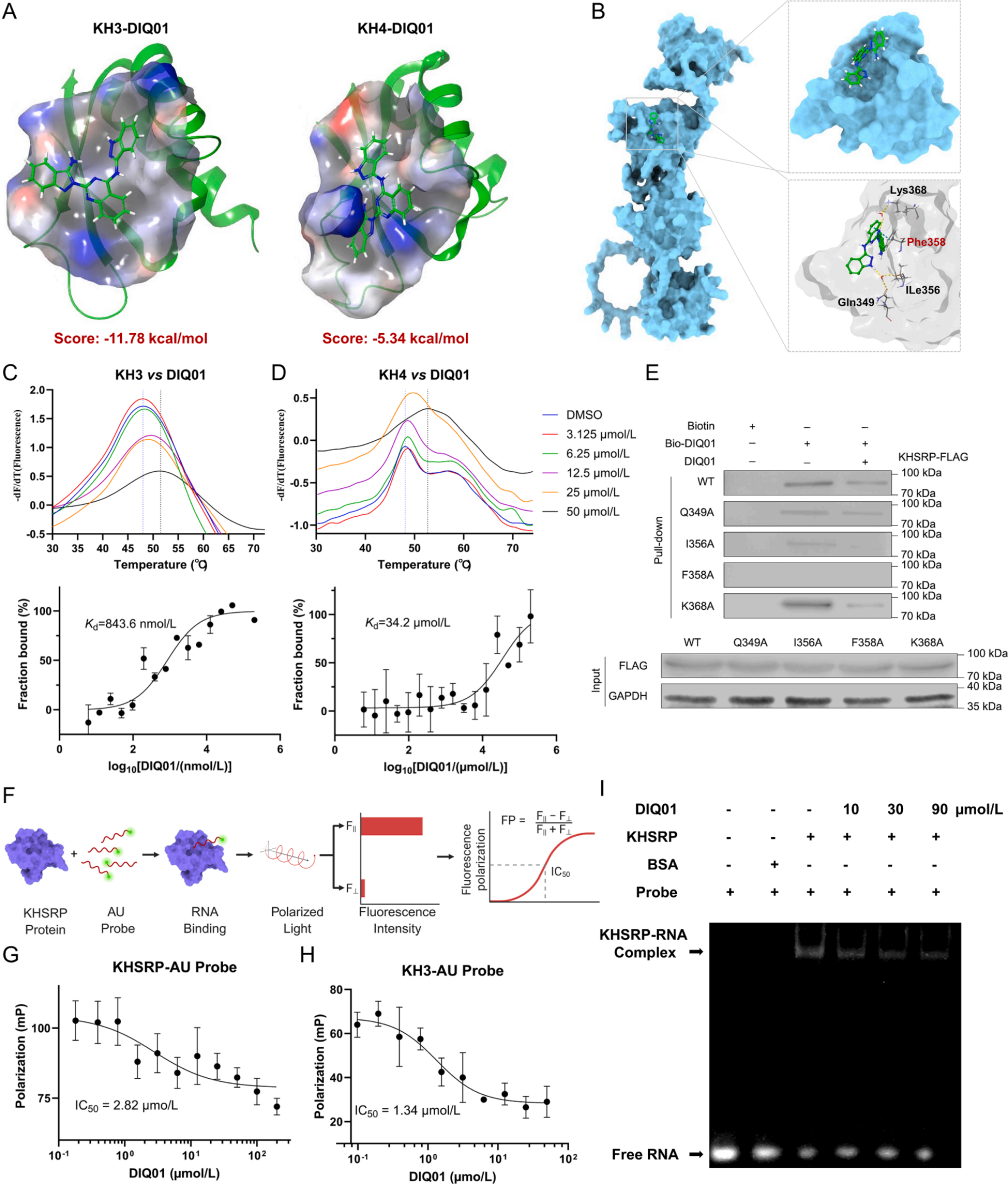
Phe358 is a key residue mediating the binding between DIQ01 and KHSRP. (A) Top surface view of DIQ01 docked to the KH3 (PDB: 2HH3) and KH4 (PDB: 2HH2) domains of KHSRP, along with corresponding docking scores. (B) Binding mode of DIQ01 within full-length KHSRP, highlighting its interactions with residues Gln349, Ile356, Phe358, and Lys368. Representative protein–ligand interactions are shown. (C) DSF (top) and MST (bottom) analyses showing the binding between DIQ01 and the KH3 domain truncation. (D) DSF (top) and MST (bottom) analyses showing the binding between DIQ01 and the KH4 domain truncation. (E) HEK293T cells were transfected with FLAG-tagged wild-type KHSRP or mutants (Q349A, I356A, F358A, and K368A). Cell lysates were incubated overnight with biotinylated DIQ01 or DIQ01; Biotin served as a control. Pull-down results were analyzed by Western blot. (F) Schematic diagram illustrating the fluorescence polarization (FP) assay design for detecting KHSRP binding to AU-rich RNA probes. (G) FP analysis of DIQ01-mediated inhibition of KHSRP (75 nmol/L)–RNA probe (10 nmol/L) binding. (H) FP analysis of DIQ01-mediated inhibition of KH3 (2 μmol/L)–RNA probe (60 nmol/L) binding. (I) EMSA showing KHSRP binding to a fluorescein-labeled RNA probe. KHSRP (1 μmol/L) was pretreated with increasing concentrations of DIQ01 and then incubated with 1 μmol/L probe. The shifted RNA–protein complex is indicated by an arrow. Error bars in (C), (D), (G) and (H) represent mean ± SD, *n* = 3.

KHSRP is a 711-amino-acid RNA-binding protein that contains four KH domains critical for RNA metabolism. Among these, the KH3 and KH4 domains predominantly mediate the degradation of ARE-containing mRNAs, with KH3 exhibiting the highest RNA-binding affinity and establishing the core RNA recognition specificity of KHSRP^16^^,^^34^^,^^50^^,^^51^. To elucidate the binding site of DIQ01 on KHSRP, we initially utilized the Schrödinger Glide module for molecular docking to generate potential binding poses and evaluate the interactions between DIQ01 and each of KHSRP's four KH domains. The top-ranked poses were subsequently refined through induced-fit docking (IFD) to account for both ligand and protein flexibility. IFD results revealed that DIQ01 predominantly interacts with the KH3 domain of KHSRP (docking score = −11.78), with weaker binding observed for the KH4 domain (docking score = −5.34) (Fig. 3A). To further validate the predicted binding modes and assess the stability of these interactions, molecular dynamics simulations of DIQ01-KH3 and DIQ01-KH4 complexes were performed using the Desmond module with the SPC water model and OPLS4 force field. This integrated computational approach confirmed a stable binding configuration of DIQ01 within the RNA-binding pocket of the KH3 domain, providing mechanistic insights into its interaction with KHSRP. Structurally, the quinazoline moiety of DIQ01 engages in a *π*–*π* stacking interaction with the aromatic side chain of Phe358 in the KH3 domain. Additionally, the quinazoline and indazole nitrogen atoms form hydrophobic contacts with KHSRP residues Val334, Ile335, and Ile346, and establish water-mediated polar interactions with Gln349, Ile356, Gln357, and Lys368 (Fig. 3B and Supporting Information Fig. S3A). To experimentally validate our computational predictions, we expressed and purified the isolated KH3 and KH4 domains of KHSRP using an *E. coli* expression system (Fig. S3B and S3C). DSF and nano-DSF assays demonstrated direct binding of DIQ01 to both KH3 and KH4 domain fragments, as evidenced by significant thermal stabilization upon ligand addition (Fig. 3C and D, Fig. S3D). However, MST analyses revealed a striking affinity differential, with DIQ01 binding approximately 40-fold more strongly to KH3 (*K*_d_ = 0.84 μmol/L) compared to KH4 (*K*_d_ = 34.2 μmol/L), confirming KH3 as the primary target domain for DIQ01 (Fig. 3C and D). To precisely identify the critical amino acid residues in KH3 that mediate DIQ01 recognition, we conducted site-directed mutagenesis, introducing individual alanine substitutions at four key residues predicted by our computational models (Fig. S3A). Pull-down assays employing Biotin-DIQ01 revealed that only the F358A mutation substantially disrupted DIQ01-KHSRP binding, while mutations at other predicted interaction sites had negligible effects (Fig. 3E). Collectively, these complementary computational and experimental approaches establish that DIQ01 selectively engages KHSRP at the KH3 domain, with Phe358 serving as a critical anchor point for molecular recognition.

#### *3.3.2* *DIQ01 inhibits KHSRP*–*RNA binding*

Having established that DIQ01 selectively targets the RNA-binding KH3 domain of KHSRP, we investigated whether this interaction disrupts KHSRP's ability to bind its target mRNAs. To directly quantify KHSRP–RNA interactions, we established a fluorescence polarization (FP) assay (Fig. 3F), using a 25-nt FAM-labeled single-stranded RNA (ssRNA) probe containing the canonical AUUUA motif, a well-characterized binding sequence recognized by KHSRP^34^^,^^51^. Titration of full-length KHSRP protein and the isolated KH3 domain against this probe yielded a concentration-dependent increase in polarization, indicating specific binding (Fig. S3E). The binding exhibited specificity and reversibility, as excess unlabeled RNA probe effectively displaced the fluorescein-labeled probe from both full-length KHSRP and the isolated KH3 domain, returning polarization signals to baseline levels (Fig. S3F). After confirming the reliability of this assay, we examined whether DIQ01 could competitively inhibit KHSRP–RNA interactions. Indeed, DIQ01 disrupted KHSRP–RNA binding in a concentration-dependent manner, with IC_50_ values of 2.82 μmol/L for full-length KHSRP (Fig. 3G) and 1.34 μmol/L for the isolated KH3 domain (Fig. 3H). We corroborated these findings using an electrophoretic mobility shift assay (EMSA), which confirmed that DIQ01 prevents formation of KHSRP–RNA complexes *in vitro* in a concentration-dependent manner (Fig. 3I). These results demonstrate that DIQ01 directly interferes with KHSRP's RNA-binding function by targeting the critical KH3 domain.

### 3.4 DIQ01 inhibits PRMT5-mediated methylation of KHSRP

**Figure 4:**
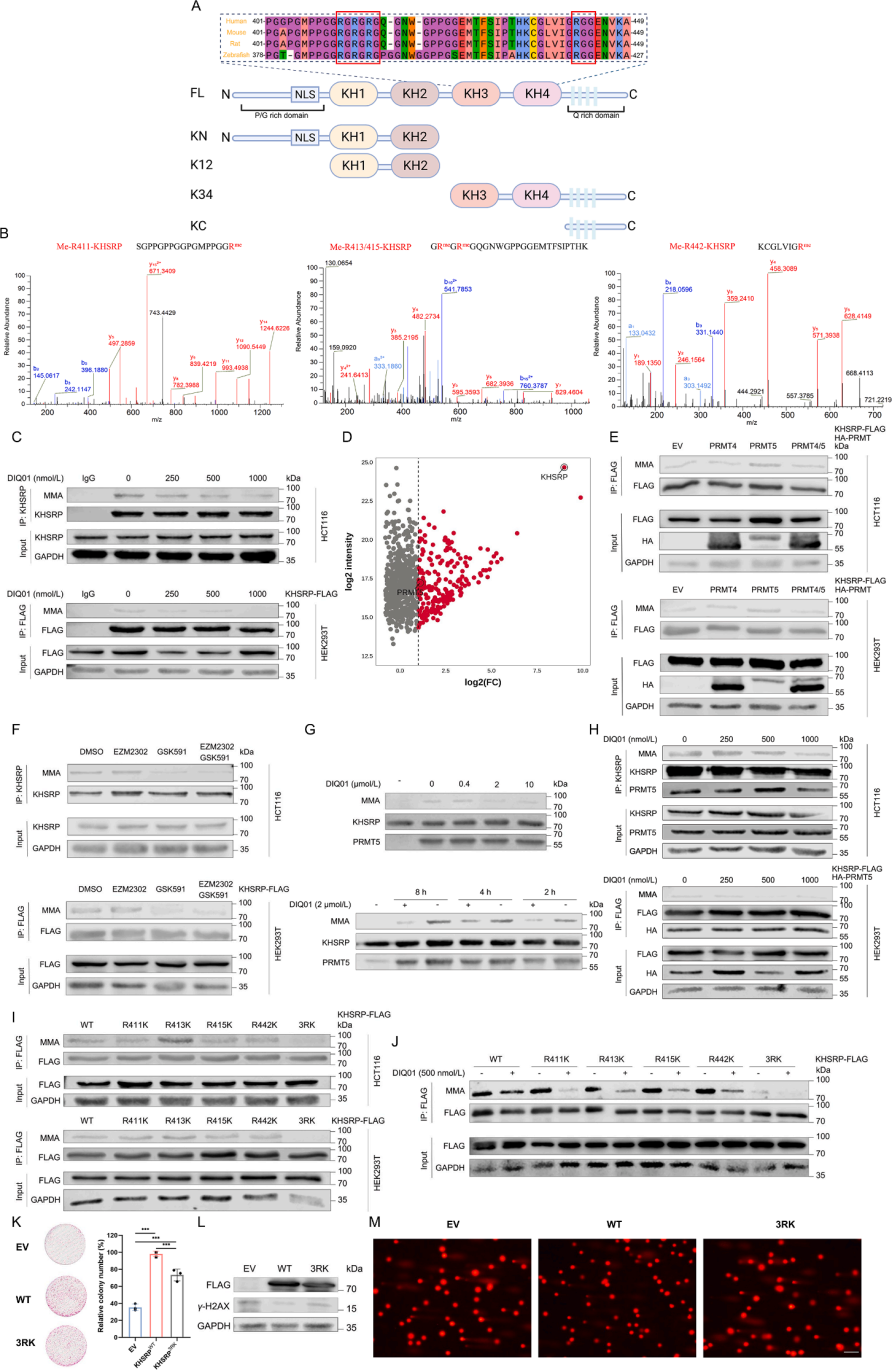
DIQ01 inhibits PRMT5-mediated methylation of KHSRP. (A) Schematic representation of KHSRP domain architecture across multiple species, highlighting conserved arginine residues (3R: R411, R413, R415; and R442) and the four truncation mutants used for interaction mapping. (B) Representative mass spectra confirming arginine methylation sites on KHSRP. (C) Detection of monomethylarginine (MMA) levels in endogenous KHSRP (HCT116) and exogenously expressed FLAG-tagged KHSRP (HEK293T) following DIQ01 treatment. KHSRP was immunoprecipitated using anti-KHSRP or anti-FLAG antibodies prior to Western blot analysis of MMA. (D) Volcano plot of proteins identified as KHSRP interactors by IP–MS in HCT116 cells. (E) HCT116 and HEK293T cells were co-transfected with FLAG-tagged KHSRP and either HA-tagged PRMT4, PRMT5, or both, with empty vector (EV) as control. KHSRP was immunoprecipitated using anti-FLAG antibody, and monomethylarginine (MMA) levels were detected by Western blot to evaluate PRMT4/5-mediated methylation of KHSRP. (F) Detection of MMA levels in endogenous KHSRP (HCT116) and exogenously expressed FLAG-tagged KHSRP (HEK293T) following treatment with PRMT inhibitors, including a PRMT4-specific inhibitor (EZM2302), a PRMT5-specific inhibitor (GSK591), or their combination. DMSO served as a vehicle control. (G) DIQ01 directly blocked PRMT5-mediated methylation of KHSRP *in vitro*. Top: DIQ01 concentration series (0–10 μmol/L; “–” no PRMT5, “0” vehicle with PRMT5). Bottom: time course with 2 μmol/L DIQ01 for 2–8 h (“+” DIQ01, “–” vehicle). MMA levels were detected by anti-MMA blot; total KHSRP and PRMT5 served as controls. Data represent ≥3 independent repeats. (H) HCT116 cells and HEK293T (co-expressing FLAG-tagged KHSRP and HA-tagged PRMT5) cells were treated with increasing concentrations of DIQ01. Co-immunoprecipitation was performed using anti-KHSRP antibody (HCT116) or anti-FLAG antibody (HEK293T), followed by immunoblotting for MMA, PRMT5, KHSRP, and FLAG. (I) FLAG-tagged KHSRP mutants carrying single arginine-to-lysine substitutions (R411K, R413K, R415K, or R442K), as well as the combined 3RK mutant, were expressed in HCT116 and HEK293T cells. Immunoprecipitation was performed using anti-FLAG antibody, followed by Western blot analysis of MMA levels. (J) HEK293T cells expressing the same KHSRP mutants (R411K, R413K, R415K, R442K, or 3RK) were treated with or without DIQ01 (500 nmol/L). MMA levels were evaluated by Western blot following FLAG-based immunoprecipitation. (K–M) Functional assays evaluating DNA damage and cell proliferation in HCT116 cells with stable KHSRP knockdown reconstituted with EV, KHSRP^WT^ or KHSRP^3RK^. (K) Colony formation assay: cells were cultured for five days, stained with SRB, and relative colony numbers were quantified (mean ± SD, *n* = 3; ∗∗∗*P* < 0.001). (L) Western blot analysis of *γ*-H2AX expression and (M) alkaline comet assay were performed after 48 h to assess DNA damage, scale bar = 5 μm.

To further elucidate the molecular mechanisms underlying DIQ01-mediated inhibition of KHSRP function, we investigated the potential involvement of post-translational modifications. Comprehensive sequence alignment of KHSRP orthologs across multiple species revealed that the linker region between the KH3 and KH4 domains is enriched in RGG/RG motifs^52^, which are canonical sites for arginine methylation (Fig. 4A). Given the limited knowledge regarding KHSRP methylation, we hypothesized that characterizing its methylation profile could provide new insights into KHSRP regulation. We initially investigated whether KHSRP undergoes arginine methylation in cellular contexts. Immunoprecipitation of endogenous KHSRP from HCT116 cells coupled with mass spectrometry analysis (IP–MS) identified four methylated arginine residues: R411, R413, R415, and R442. Notably, three of these residues (R411/413/415) cluster within an RGRGRG sequence motif located in the KH3–KH4 linker region; we designated this conserved triad as “3R” (Fig. 4B). Western blot analysis using a pan-monomethylarginine (MMA) antibody further validated the presence of arginine methylation on KHSRP in cellular environments (Supporting Information Fig. S4A). Subsequently, we examined whether DIQ01, which binds in proximity to the KH3–KH4 interface, could modulate KHSRP's methylation status. Remarkably, DIQ01 treatment elicited a dose-dependent reduction in the MMA signal on immunoprecipitated KHSRP, a phenomenon observed with both endogenous KHSRP in HCT116 cells and ectopically expressed FLAG-tagged KHSRP in HEK293T cells (Fig. 4C).

To identify the arginine methyltransferase (PRMT) responsible for KHSRP methylation, we performed an integrative proteomic analysis. IP–MS analysis of KHSRP-interacting proteins in HCT116 cells (Fig. 4D), combined with ABPP-MS (Fig. 2C), highlighted PRMT4 and PRMT5 as candidate enzymes. Co-immunoprecipitation experiments confirmed that KHSRP interacts with both PRMT4 and PRMT5 in HCT116 cells (endogenous KHSRP) and HEK293T cells (overexpressed FLAG–KHSRP) (Fig. S4B). To determine which enzyme actually mediates the methylation of KHSRP, we modulated the expression levels and enzymatic activity of PRMT4 and PRMT5. Overexpression of PRMT5 led to an increase in KHSRP MMA signal, whereas overexpression of PRMT4 had little additional effect (Fig. 4E). Furthermore, treating cells with a selective PRMT5 inhibitor (GSK591) dramatically reduced KHSRP methylation, while the PRMT4 inhibitor (EZM2302) showed no obvious effect (Fig. 4F). These results establish PRMT5 as the primary methyltransferase responsible for arginine methylation of KHSRP.

Having identified PRMT5 as the major enzyme mediating KHSRP methylation, we next established an *in vitro* PRMT5-KHSRP methylation assay to determine whether DIQ01 directly affects this process. Recombinant PRMT5 robustly methylated full-length KHSRP, as detected by anti-MMA immunoblotting, and this signal was reduced by the selective PRMT5 inhibitor GSK591, validating the assay specificity (Fig. S4C). DIQ01 decreased PRMT5-mediated KHSRP methylation in a concentration-dependent manner and also produced time-dependent suppression at 2 μmol/L (Figs. 4G and S4D). Importantly, DIQ01 did not induce a detectable decrease in global arginine methylation patterns in whole-cell lysates (Fig. S4E). Moreover, DSF analysis of recombinant PRMT5 showed no appreciable thermal shift across 1–100 μmol/L DIQ01, indicating no detectable direct binding or stabilization of PRMT5 (Fig. S4F). These data suggest that DIQ01 does not act as a general PRMT5 inhibitor, but selectively impairs PRMT5-mediated methylation of KHSRP.

We then mapped the PRMT5-interacting region on KHSRP by generating a panel of truncation mutants spanning different regions of the protein (KN, K12, K34, and KC) (Fig. 4A). Among the constructs tested, only the K34 fragment encompassing residues 321–520 (covering the KH3 and KH4 domains) retained the ability to bind PRMT5, identifying this region as the interface for PRMT5 interaction (Fig. S4G). Given that DIQ01 binds to both domains, we hypothesized that DIQ01 may inhibit KHSRP methylation by affecting PRMT5's access to this site. However, DIQ01 treatment did not disrupt the physical interaction between KHSRP and PRMT5, as determined by Co-IP assays (Fig. 4H). We speculated that DIQ01 binding might induce an allosteric conformational change in the KH3–KH4 region, thereby indirectly affecting PRMT5's catalytic activity toward KHSRP. Therefore, we subjected purified KHSRP to limited proteolysis (LiP). In trypsin digests (cleaving C-terminally to lysine and arginine) and Arg-C digests (cleaving C-terminally to arginine), DIQ01 consistently increased the number of discrete fragments and altered banding patterns, indicating DIQ01-dependent changes in protease accessibility consistent with conformational remodeling (Supporting Information Fig. S5). Two proteases with complementary specificities were employed to minimize enzyme-specific bias. Arg-C proved particularly informative for regions rich in RGG/RG motifs near PRMT5 methylation sites on KHSRP, while trypsin provided broader coverage of both lysine and arginine contexts. Together with the observed thermal shifts in DSF assays (Figs. 2I, 3C and D), these data support the conclusion that DIQ01 induces conformational remodeling of KHSRP.

To further identify the key methylation sites on KHSRP, we engineered a series of arginine-to-lysine substitution mutants: four single-point variants (R411K, R413K, R415K, R442K) and one triple mutant (3RK: R411/413/415K). Our analyses demonstrated that while individual substitutions had minimal impact, only the 3RK triple mutant exhibited substantial abrogation of KHSRP MMA methylation in both HCT116 and HEK293T cell lines (Fig. 4I). We next asked whether DIQ01 shows site selectivity. DIQ01 suppressed the residual MMA signal in every mutant, demonstrating that it effectively blocks methylation at each identified site (Fig. 4J). To evaluate the functional consequences of KHSRP arginine methylation, we performed rescue experiments in KHSRP-depleted HCT116 cells by reintroducing either wild-type KHSRP (KHSRP^WT^) or the methylation-deficient 3RK mutant (KHSRP^3RK^). Colony formation assays revealed that 3RK-reconstituted cells exhibited a reduced proliferative capacity, yielding fewer colonies (Fig. 4K). Consistently, cells expressing methylation-deficient KHSRP exhibited markers of heightened genomic instability, including elevated *γ*-H2AX levels detected by Western blot (Fig. 4L) and increased DNA fragmentation, as evidenced by longer tail moments in comet assays (Fig. 4M). Together, these findings indicated that arginine methylation is required for KHSRP to fully exert its pro-proliferative and genome-protective functions in HCT116 cells.

### 3.5 DIQ01 suppresses colorectal cancer growth *in vivo*

**Figure 5:**
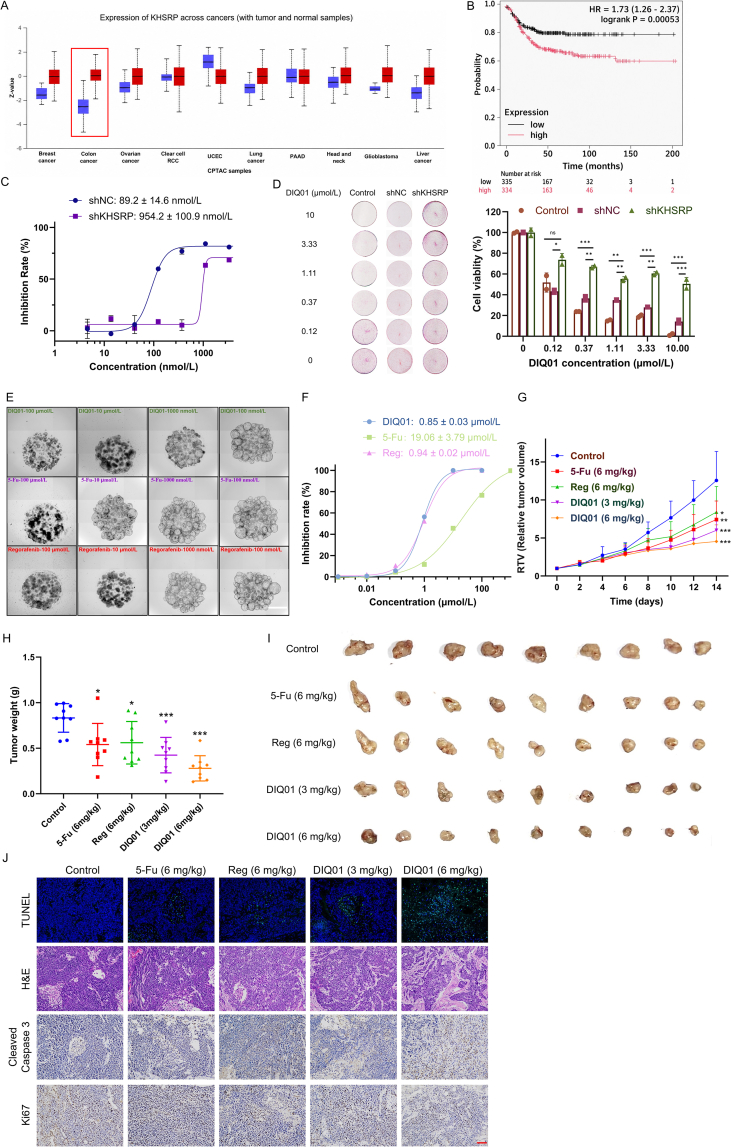
DIQ01 suppresses KHSRP-dependent colorectal cancer growth in organoid and xenograft models. (A) Pan-cancer analysis of KHSRP protein expression using the UALCAN database. Red indicates tumor tissues, and blue represents normal tissues. Colon cancer is highlighted with a red box, showing significantly elevated KHSRP expression in tumors. (B) Kaplan–Meier analysis of overall survival based on KHSRP expression in colon cancer patients, performed using the KMplot database (http://kmplot.com). (C) Dose–response curves in HCT116 cells stably expressing control shRNA (shNC) or KHSRP shRNA (shKHSRP). Growth-inhibition (%) was quantified after DIQ01 treatment; data are mean ± SD (*n* = 3). (D) Left: SRB staining images of HCT116 cells treated with a gradient concentration of DIQ01 (0, 0.12, 0.37, 1.11, 3.33, 10.00 μmol/L) for 36 h in 24-well plates under normal, shKHSRP, and shNC conditions. Right: Bar graph showing the cell viability (%) based on SRB assay. (E) Representative brightfield images of CRC patient-derived organoids treated with DIQ01, 5-Fu, or regorafenib at the indicated concentrations. Scale bars, 1 mm. (F) Drug dose–response curves of CRC patient-derived organoids treated with the DIQ01, 5-Fu and regorafenib. Growth-inhibition (%) was quantified after compound treatment; data are mean ± SD (*n* = 3). (G) Tumor growth curves showing average tumor volume in each group over the treatment period following administration of DIQ01, vehicle control, or positive control drugs. (H) Final tumor weights from individual mice across the five treatment groups. (I) Representative images of excised tumors from mice in each group. (J) Histological and immunohistochemical analysis of tumor tissues, including TUNEL staining, H&E staining, and immunohistochemical staining for cleaved caspase-3 and Ki-67. Scale bar = 100 μm. Error bars in (C, D, F–H) represent mean ± SD. Sample sizes: *n* = 3 (C–F), *n* = 9 (G–I). Statistical significance was determined relative to the control group: ∗*P* < 0.05, ∗∗*P* < 0.01, and ∗∗∗*P* < 0.001.

Having demonstrated that DIQ01 functionally inactivates KHSRP *via* two distinct mechanisms (blocking RNA binding and inhibiting methylation), we next examined whether these molecular effects translate into antitumor activity. Initially, we analyzed KHSRP expression across various cancer types using publicly available UALCAN and GEPIA datasets. In CRC patient samples, both *KHSRP* mRNA and KHSRP protein levels were significantly overexpressed in tumors relative to normal colon tissue (Fig. 5A, and Supporting Information Fig. S6A). Furthermore, Kaplan–Meier analysis of clinical data revealed that CRC patients with higher KHSRP expression had markedly poorer overall survival (Fig. 5B). These correlative data implicate KHSRP as a potential oncogenic protein in colorectal cancer and a viable therapeutic target. To directly assess KHSRP's functional role in the growth of colorectal cancer cells, we conducted both transient and stable KHSRP knockdown in HCT116 cells. Colony formation assays demonstrated that the loss of KHSRP significantly impaired the proliferative capacity of HCT116 cells (Fig. S6B–S6E). Moreover, in rescue experiments, reintroducing wild-type KHSRP into KHSRP-knockdown cells restored their viability, while an empty-vector control had no effect (Fig. S6F). Notably, the antitumor efficacy of DIQ01 was significantly diminished in KHSRP-knockdown HCT116 cells, with an 11-fold increase in IC_50_ compared to control cells expressing intact KHSRP, indicating that DIQ01's antitumor activity is mediated through KHSRP (Fig. 5C and D). In patient-derived colorectal cancer organoid models, DIQ01 still retained robust antiproliferative activity, with an IC_50_ of 0.85 μmol/L, markedly lower than that of 5-Fu (19.06 μmol/L) and slightly below that of regorafenib (0.94 μmol/L) (Fig. 5E and F).

Next, we evaluated the antitumor efficacy of DIQ01 in an *in vivo* colorectal cancer xenograft model. Acute toxicity studies indicated that the median lethal dose (LD_50_) of DIQ01 exceeded 300 mg/kg (Fig. S6G–S6I). Given the suboptimal pharmacokinetics of DIQ01, characterized by poor oral bioavailability and highly variable systemic exposure (Supporting Information Table S5), systemic administration by the oral or intraperitoneal route was unlikely to maintain therapeutic drug levels. We therefore chose intratumoral injection to ensure reliable and localized delivery. In mice bearing HCT116 xenografts, DIQ01 treatment produced a robust tumor-suppressive effect. Mice were treated with DIQ01 at doses of either 3 or 6 mg/kg, and both doses significantly inhibited tumor growth relative to vehicle controls. By the end of the study, DIQ01 achieved tumor growth inhibition (TGI) rates of approximately 52% (3 mg/kg) and 64% (6 mg/kg), accompanied by tumor weight reductions of 43% (3 mg/kg) and 64% (6 mg/kg), respectively. Notably, DIQ01 outperformed equimolar doses of 5-Fu (TGI: 41% and tumor weight reduction: 34%) and regorafenib (TGI: 33% and tumor weight reduction: 34%) in this model (Fig. 5G–I). Throughout treatment, DIQ01 administration did not result in any significant body weight loss, suggesting minimal systemic toxicity (Fig. S6J). Histopathological analysis of tumor tissues *via* H&E staining revealed extensive nuclear condensation and morphological features of apoptosis after DIQ01 treatment. This observation was corroborated by TUNEL assays and immunohistochemical staining for cleaved caspase-3 and the proliferation marker Ki-67, which demonstrated increased apoptotic cell death and reduced proliferative activity in DIQ01-treated tumors (Fig. 5J). Western blot analysis of tumor lysates revealed increased *γ*-H2AX expression following DIQ01 treatment, consistent with DNA damage induction (Fig. S6K). Importantly, histological examination of major organs revealed no signs of tissue damage or overt toxicity in DIQ01-treated mice (Fig. S6L). Collectively, these findings demonstrate that DIQ01, a novel small-molecule inhibitor of KHSRP, effectively suppresses colorectal tumor growth *in vivo* while exhibiting a favorable safety profile, underscoring the therapeutic potential of targeting KHSRP in colorectal cancer.

### 3.6 DIQ01 induces DNA damage by KHSRP-mediated PLK1 suppression

**Figure 6:**
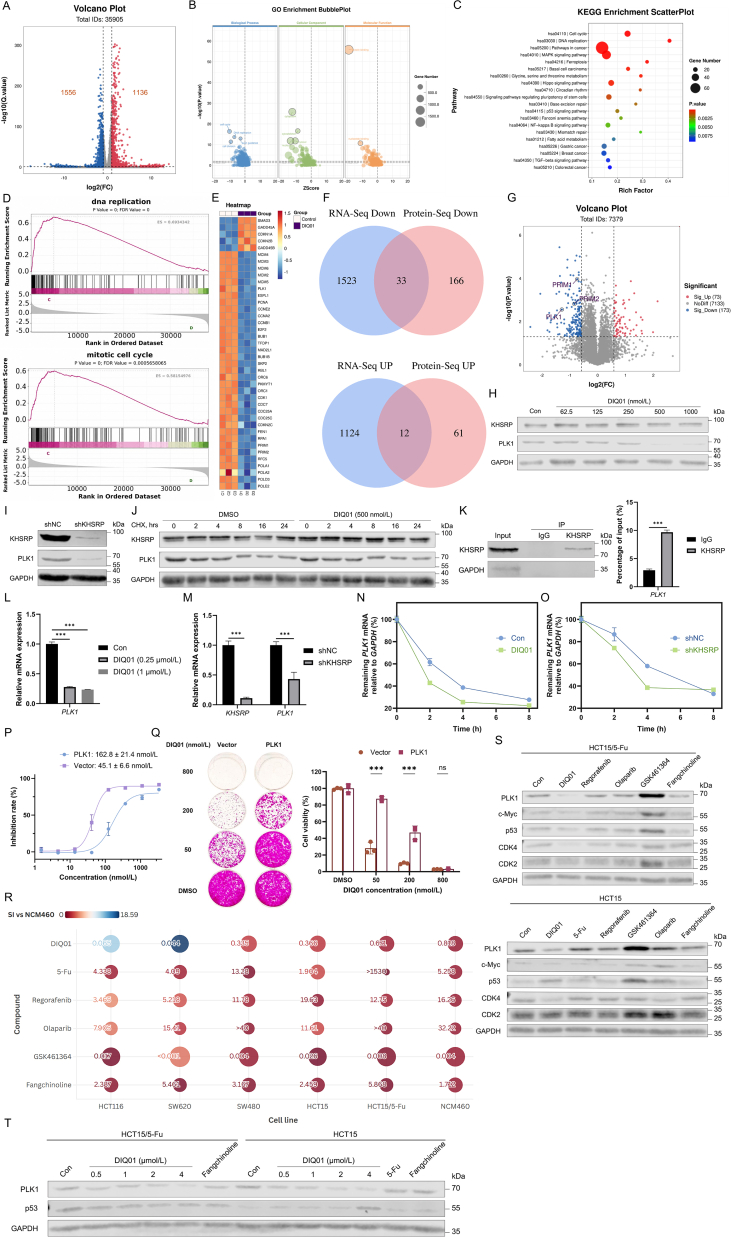
DIQ01 induces DNA damage by KHSRP-mediated PLK1 suppression. (A) Volcano plot of differentially expressed genes in HCT116 cells following DIQ01 treatment (500 nmol/L, 24 h), based on RNA-seq analysis. (B) Gene Ontology (GO) enrichment analysis of differentially expressed genes, shown as a bubble plot. (C) KEGG pathway enrichment analysis of DIQ01-regulated transcripts. (D) Gene set enrichment analysis (GSEA) plots showing negative enrichment of DNA replication and mitotic cell cycle gene sets in DIQ01-treated cells. ES, enrichment score. (E) Heatmap of differentially expressed genes associated with DNA replication and cell cycle progression. (F) Venn diagram showing the overlap of upregulated and downregulated genes identified by RNA-seq and proteomic analysis after DIQ01 treatment. (G) Volcano plot of differentially expressed proteins identified by proteomics in DIQ01-treated HCT116 cells, highlighting PLK1, PRIM1, and PRIM2. (H) Western blot analysis showing dose-dependent downregulation of PLK1 protein in HCT116 cells treated with increasing concentrations of DIQ01. (I) Western blot analysis showing reduced PLK1 expression in HCT116 cells with stable KHSRP knockdown. (J) Cycloheximide (CHX, 100 μg/mL) chase assay showing the time-dependent stability of PLK1 protein in HCT116 cells treated with DIQ01 (500 nmol/L) or DMSO. GAPDH served as the internal control. (K) RNA immunoprecipitation (RIP) analysis confirming the binding of KHSRP to *PLK1* mRNA in HCT116 cells, validated by Western blot and RT-qPCR. (L) RT-qPCR analysis of *PLK1* mRNA levels in HCT116 cells treated with 0.25 or 1 μmol/L DIQ01. (M) RT-qPCR analysis of *KHSRP* and *PLK1* mRNA expression in HCT116 cells with shKHSRP versus shNC. (N) Decay of *PLK1* mRNA following actinomycin D treatment in HCT116 cells with or without DIQ01 (1 μmol/L), measured by RT-qPCR. (O) Decay of *PLK1* mRNA in HCT116 cells stably expressing shKHSRP or shNC after actinomycin D treatment, assessed by RT-qPCR. *GAPDH* was used as an internal control. Error bars in K–O represent mean ± SD, *n* = 3. Statistical significance: ∗∗∗*P* < 0.001 compared to control. (P) Dose–response curves for HCT116 cells transfected with PLK1 or an empty vector, exposed to DIQ01 for 72 h. Viability was quantified by SRB staining. Data are mean ± SD (*n* ≥ 3). (Q) SRB staining of HCT116 cells 2 days after transfection with PLK1 or vector, followed by DIQ01 treatment at 0, 50, 200, or 800 nmol/L for 5 days. Left, representative SRB-stained wells; right, quantification of SRB absorbance normalized to DMSO. Bars represent mean ± SD (*n* ≥ 3); ∗∗∗*P* < 0.001; ns, not significant. (R) Bubble plot showing drug activity in HCT116, SW620, SW480, HCT15, the 5-Fu–resistant subline HCT15/5-Fu, and non-malignant colonic epithelial cells (NCM460). The number inside each bubble is the IC_50_ (μmol/L). Bubble area is proportional to potency (linearly scaled to −log_10_(IC_50_), so lower IC_50_ → larger bubble). Colour encodes the selectivity index relative to NCM460, SIIC_50_(NCM460)/IC_50_ (tumour); higher SI (blue end of the colour scale) indicates greater tumour selectivity/larger therapeutic window. The NCM460 column reports activity in the normal cell line (its SI is defined as 1). Censored measurements are displayed at the assay bounds as <0.001 μmol/L, ≥40 μmol/L, or ≥1530 μmol/L as indicated. (S) Western blot analysis of PLK1, c-Myc, p53, CDK4, and CDK2 in HCT15/5-Fu (top) and parental HCT15 (bottom) cells following 24 h treatment with DIQ01 (1 μmol/L), 5-Fu (10 μmol/L), regorafenib (20 μmol/L), the PLK1 inhibitor GSK461364 (50 nmol/L), olaparib (40 μmol/L), or fangchinoline (10 μmol/L). GAPDH was used as a loading control. (T) HCT15/5-Fu cells and parental HCT15 cells were treated with increasing concentrations of DIQ01 (0.5–4 μmol/L) for 24 h, with 5-Fu (10 μmol/L) and fangchinoline (10 μmol/L) as controls. The expression of PLK1 and p53 was assessed by Western blot, with GAPDH as a loading control.

To further elucidate the downstream effects of DIQ01-mediated KHSRP inhibition, we conducted comprehensive transcriptomic profiling of DIQ01-treated HCT116 cells. Volcano plot analysis revealed substantial transcriptional reprogramming, with 1556 genes significantly upregulated and 1136 genes downregulated (Fig. 6A). Gene Ontology (GO) enrichment analysis demonstrated that these differentially expressed genes cluster in pathways controlling cell cycle, DNA replication, and cell division, all of which are essential for cellular proliferation and survival (Fig. 6B and Supporting Information Fig. S7A). Correspondingly, KEGG pathway mapping identified significant downregulation of cell cycle, DNA replication, and p53 signaling pathways, together with perturbations in MAPK, Hippo, and apoptotic signaling cascades (Fig. 6C, Fig. S7B and S7C). Gene Set Enrichment Analysis (GSEA) further confirmed that DIQ01 preferentially attenuates gene sets associated with mitotic progression and DNA synthesis (Fig. 6D and Fig. S7D). These transcriptomic alterations align with our earlier functional data (Fig. 1F–I), and support a model in which DIQ01 blocks KHSRP, impairs DNA replication, and thereby induces genomic instability, cell-cycle arrest and apoptosis.

To identify the key mediators downstream of DIQ01, we combined transcriptomic and proteomic datasets. RNA-seq revealed 40 genes significantly altered by DIQ01 and annotated to cell-cycle or DNA-replication pathways (Fig. 6E). Subsequently, overlaying quantitative proteomics data and retaining only those genes whose protein abundance changed concordantly with their mRNA reduced the list to three: *PLK1*, *PRIM1* and *PRIM2* (Fig. 6F and G). Among these, PLK1 showed the highest overall expression and displayed the strongest positive correlation with KHSRP in colorectal-cancer samples, as queried in the GEPIA database (Fig. S7E and S7F). Extensive studies have established that PLK1, a member of the serine/threonine kinase family, plays a crucial role in driving cell cycle progression and tumor proliferation in colorectal cancer^53^, ^54^, ^55^, ^56^. Collectively, these observations nominate PLK1 as the most likely downstream effector of DIQ01, and we therefore focused subsequent experiments on this kinase.

Further experiments confirmed that DIQ01 down-regulates PLK1 expression. Western blot analysis showed that DIQ01 treatment led to a dose-dependent decrease in PLK1 protein levels (Fig. 6H), phenocopying the effect of KHSRP knockdown (Fig. 6I). To determine whether this regulation occurs at the level of protein stability, we performed cycloheximide (CHX) chase assays. DIQ01 did not appreciably alter the decay rate or half-life of PLK1 protein after CHX treatment, suggesting that DIQ01 does not reduce PLK1 abundance by accelerating protein degradation (Fig. 6J). We next investigated whether KHSRP regulates PLK1 expression at the RNA level. RIP-qPCR assays confirmed that KHSRP directly binds to *PLK1* mRNA (Fig. 6K). Consistently, both DIQ01 treatment and KHSRP knockdown significantly reduced *PLK1* mRNA levels (Fig. 6L and M). Actinomycin D chase assays further demonstrated that DIQ01 reduced the stability of *PLK1* transcripts (Fig. 6N), an effect also observed upon KHSRP depletion (Fig. 6O). In line with this mechanism, DIQ01 treatment in HCT116 cells induced a concentration-dependent increase in *γ*-H2AX levels, which coincided with progressive PLK1 downregulation (Fig. S7G). To assess whether the DIQ01 phenotype involves the KHSRP–PLK1 axis, we ectopically expressed PLK1. PLK1 overexpression shifted the IC_50_ of DIQ01 by approximately fourfold (from 45.1 ± 6.6 to 162.8 ± 21.4 nmol/L), indicating reduced drug sensitivity (Fig. 6P and Q). Moreover, exogenous PLK1 expression counteracted DIQ01-induced CDK2 downregulation and limited p53 induction, whereas KHSRP knockdown attenuated DIQ01-mediated PLK1 downregulation (Fig. S7H and S7I). Collectively, these results demonstrate that DIQ01 suppresses colorectal cancer cell proliferation by disrupting KHSRP-mediated post-transcriptional regulation of *PLK1*, ultimately impairing DNA replication, disturbing cell cycle progression, and inducing genomic instability.

DIQ01 exhibited submicromolar IC_50_ values across all tested colorectal cancer cell lines (HCT116, SW620, SW480, and HCT15) and maintained potent activity in the 5-Fu-resistant HCT15/5-Fu model (IC_50_ = 0.671 ± 0.074 μmol/L) (Supporting Information Table S6 and Fig. S8A–S8F). In contrast, 5-Fu showed markedly reduced potency in this resistant line, with an IC_50_ exceeding 1.53 mmol/L, while regorafenib and olaparib were effective only at high micromolar concentrations. Notably, the KHSRP inhibitor fangchinoline exhibited an IC_50_ of 5.868 ± 0.195 μmol/L, approximately ten-fold higher than that of DIQ01. When using NCM460 normal cells as a reference, fangchinoline displayed selectivity indices (SI) below 1 across all tumor cell lines, indicating a lack of therapeutic specificity (Fig. 6R). In comparison, DIQ01 demonstrated significantly improved selectivity, with SI values of approximately 12.6 in HCT116, 18.6 in SW620, underscoring its favorable therapeutic window (Fig. 6R). Although the PLK1 inhibitor GSK461364 exhibited high potency in tumor cells, it also showed strong activity in NCM460 normal cells (IC_50_ = 0.004 μmol/L) (SI range: 0.15–4.0), reflecting limited selectivity (Fig. 6R). Together, these results highlight that DIQ01 possesses not only high potency but also enhanced selectivity toward cancer cells.

In HCT116 cells, DIQ01 induced a dose-dependent decrease in PLK1 levels, accompanied by increased *γ*-H2AX expression, supporting its ability to trigger DNA damage. DIQ01 also decreased the levels of c-Myc, CDK2, and CDK4, while increasing p53 expression. In comparison, most reference compounds did not reduce total PLK1 protein levels; notably, the PLK1 kinase inhibitor GSK461364 did not decrease PLK1 abundance, consistent with its role as a catalytic inhibitor rather than a regulator of PLK1 expression (Fig. S8G and S8H). Similar effects were observed in parental HCT15 and 5-Fu-resistant HCT15/5-Fu cells. DIQ01 reduced PLK1, c-Myc, and CDK4 expression in both cell lines, whereas regorafenib and other reference inhibitors failed to consistently downregulate PLK1 (Fig. 6S and T). The effect on p53 was cell-context dependent: p53 increased in HCT15 cells, whereas the elevated basal p53 level in HCT15/5-Fu cells was reduced after DIQ01 treatment (Fig. 6T). Together, these results indicate that DIQ01 maintains potent activity in multiple colorectal cancer models, including a 5-Fu-resistant context, and is distinguished from reference agents by its coordinated suppression of PLK1-associated proliferative and cell-cycle regulatory proteins.

To explore the combination potential, we profiled DIQ01 in combination with 5-Fu or the PARP inhibitor olaparib across HCT116, HCT15, and HCT15/5-Fu cell lines using 6 × 6 dose matrices. Drug interactions were analyzed in SynergyFinder 3.0 with the ZIP model and baseline correction, with inhibition (%) as the response readout^57^. Following standard practice, ZIP > +10 indicates synergy, −10 to +10 indicates additivity, and < −10 indicates antagonism. The most synergistic area (MSA) is interpreted similarly. As shown in Supporting Information Fig. S8I–S8N, the combination of DIQ01 and 5-Fu was additive to weakly synergistic in HCT116 (ZIP +2.176; MSA 6.43) and near-additive in HCT15 (ZIP −0.566; MSA 1.56), whereas in HCT15/5-Fu it showed a modest global synergy signal with a distinct synergistic hotspot (ZIP +6.159; MSA 10.99). In contrast, DIQ01 in combination with olaparib was near-additive in HCT116 (ZIP −0.797, MSA 3.10) and HCT15 (ZIP −0.986, MSA 2.16), and showed no synergy with a trend toward antagonism in HCT15/5-Fu (ZIP −8.687, MSA −0.73). These results suggest that DIQ01 has a more favorable combination profile with 5-Fu than with olaparib, particularly in the 5-Fu-resistant setting, whereas PARP inhibitor-based synergy appears to be model-dependent under our experimental conditions.

Based on the collective evidence, this study establishes that DIQ01 mediates potent and selective antitumor activity in multiple colorectal cancer models, including a 5-Fu-resistant model, through targeting KHSRP and consequent disruption of PLK1-dependent signaling. The compound demonstrates a favorable selectivity profile, target-specific mechanisms of action, and coordinated downregulation of proliferation-related proteins, with preserved efficacy in resistant contexts. These findings support DIQ01 as a promising candidate for further therapeutic development (Supporting Information Fig. S9–S24).

## 4 Discussion

RBPs play a crucial role in orchestrating post-transcriptional gene regulation and are frequently dysregulated in cancer, impacting the expression of both oncogenes and tumor suppressors^58^^,^^59^. KHSRP has been linked to tumourigenesis and correlates with poor patient prognosis^18^. Classically, KHSRP recognises AU-rich elements (AREs) in the 3′-UTR of target mRNAs through its KH3 domain, recruiting the exosome and poly(A) ribonuclease (PARN) to facilitate rapid mRNA decay^16^. Recent evidence suggests that KHSRP can also act as a context-dependent mRNA stabilizer. For example, the prostate cancer-associated lncRNA PRCAT71 scaffolds KHSRP onto androgen receptor (*AR*) mRNA, enhancing *AR* mRNA stability and signaling^60^. Similarly, KHSRP recognizes m^6^A-modified transcripts of key genes in the FAK signaling pathway in pancreatic cancer, stabilizing them to activate FAK signaling^19^. These observations raise the possibility that transcripts lacking canonical AREs may still be regulated by KHSRP *via* alternative mechanisms. PLK1, a key serine/threonine kinase that drives mitosis, is frequently over-expressed in many tumors, including colorectal cancer, where high PLK1 levels are associated with invasion, metastasis and poor outcome^61^^,^^62^. Through integrated transcriptomic and proteomic analysis, we identified PLK1 as a representative target of DIQ01-mediated KHSRP inhibition in HCT116 cells. Both KHSRP knock-down and DIQ01 exposure significantly decreased *PLK1* mRNA and protein in HCT116 cells, indicating that KHSRP positively regulates PLK1 expression. Furthermore, PLK1 overexpression could reduce the sensitivity of HCT116 cells to DIQ01. Therefore, DIQ01 disrupts the KHSRP–PLK1 axis and consequently attenuates PLK1-driven malignant phenotypes such as uncontrolled proliferation and enhanced DNA repair.

Our head-to-head benchmarking clarifies the potential advantages of targeting the KHSRP–PLK1 axis with DIQ01 rather than inhibiting PLK1 catalysis directly. Across colorectal-cancer models, DIQ01 maintained submicromolar potency, preserved activity in the HCT15/5-Fu line, and showed a wider selectivity window when normalized to NCM460. Consistent with its distinct mechanism, DIQ01 induced dose-dependent downregulation of PLK1, c-Myc, and CDK4, along with *γ*-H2AX induction. In contrast, GSK461364 did not reduce total PLK1 and occasionally increased its levels, while exhibiting minimal effects on other cell-cycle regulators beyond p53. These observations suggest that perturbing KHSRP-dependent post-transcriptional control of PLK1 may enlarge the therapeutic window relative to direct PLK1 blockade in proliferating normal tissues and may be less susceptible to kinase-domain resistance mutations.

Post-translational modifications (PTMs) constitute a critical regulatory mechanism that modulates the diverse functions of RBPs. Among these modifications, arginine methylation is particularly prevalent in RBPs, often targeting RGG/RG motifs^52^ that facilitate RNA and protein binding. In tumors, protein arginine methyltransferases (PRMTs) are frequently upregulated, and aberrant methylation of RBPs has been implicated in tumorigenesis^63^. For instance, PRMT1/5/7 methylate numerous RBPs, including heterogeneous nuclear ribonucleoproteins (hnRNPs), splicing factors, and translation regulators^64^, ^65^, ^66^, ^67^. The loss of methylation can disrupt RNA processing and stress responses. We identified that KHSRP undergoes arginine methylation, primarily mediated by PRMT5. This methylation occurs at specific residues in the KH3–KH4 linker region, marked by an RGG/RG motif. Methylation is essential for KHSRP's roles in promoting cell proliferation and safeguarding genomic integrity, as its absence leads to diminished cell growth and heightened genomic instability. The compound DIQ01 specifically targets the KH3 and KH4 domains of KHSRP, reducing its methylation without affecting overall arginine methylation levels, ultimately leading to suppressed proliferation and increased DNA damage. Although the precise mechanism remains to be elucidated, we hypothesize that DIQ01 binding induces a conformational change or creates a steric blockade that prevents PRMTs from accessing the methylation sites on KHSRP. Small molecules are known to interfere with protein modifications and interactions, either allosterically or sterically. For example, WX-02-23 is a covalent ligand that modifies SF3B1, inhibiting spliceosome assembly *in vitro* and similarly enhancing SF3B1 thermostability^68^. Oleic acid binds to the N-terminal RRM domain of MSI1, inducing a conformational change^69^. By analogy, we propose that DIQ01 binding induces a conformational rearrangement or establishes a steric barrier that prevents PRMT5 from accessing arginine methylation sites on KHSRP. Since inhibitors targeting RBPs that directly modulate their post-translational modifications are rarely reported, our discovery offers a valuable contribution to this underexplored area of research.

Three agents with KHSRP inhibitory activity have been identified, but their mechanisms and specificity have limitations. Fangchinoline, a natural bis-benzylisoquinoline alkaloid, was reported to bind KHSRP with moderate affinity (*K*_d_ = 245.5 μmol/L)^27^. Nevertheless, it lacks domain-specific interaction mapping. And its polypharmacological profile, involving multiple molecular targets, also reduces its efficacy as a selective KHSRP probe^70^, ^71^, ^72^, ^73^. Compared to DIQ01, fangchinoline showed markedly inferior performance, displaying antitumor activity only at low micromolar concentrations and poor selectivity (selectivity indices, SIs <1), indicating a compromised therapeutic window. GO-Y086, a synthetic analogue of curcumin, engages KHSRP's C-terminal region and disrupts its regulation of c-Myc^26^. However, its mechanism of action relies on a broadly reactive enone structure, raising concerns about potential off-target effects^74^, ^75^, ^76^, ^77^. DKC1125 is a synthetic small molecule that binds the KH4 domain of KHSRP and disrupts the oncogenic KITENIN–KHSRP–Dvl2 complex^25^. Nevertheless, it has not been shown to directly block KHSRP's RNA-binding activity. In contrast, DIQ01 was identified as a direct KHSRP ligand using multiple chemical proteomics approaches. Structure-based docking, MST, and DSF confirmed that DIQ01 binds to both the KH3 and KH4 domains of KHSRP, with Phe358 identified as a key site *via* site-directed mutagenesis. Further, FP and EMSA assays demonstrated that DIQ01 effectively prevented RNA binding to KHSRP. Functionally, DIQ01 disrupts the KHSRP–PLK1 axis and shows potent antitumor activity in colorectal cancer cell lines, organoids, and *in vivo* models, outperforming 5-Fu and regorafenib.

Currently, the therapeutic potential of DIQ01 is constrained by its low oral bioavailability, which is attributed to poor solubility/permeability and significant first-pass metabolism (Supporting Information Table S5)^78^^,^^79^. A multi-pronged optimization strategy is being pursued to address these limitations: a) designing bioreversible prodrugs (*e.g.*, phosphate or L-valyl moiety) at the 3-aminoindazole group to enhance dissolution and absorption^80^; b) developing lipid-based self-emulsifying systems (SEDDS/SMEDDS), amorphous solid dispersions, and nanosuspensions to improve solubility and stability^79^^,^^81^; c) modifying the structure through salt/polymorph screening, N-alkylation to reduce polarity, introducing a tertiary amine for salt formation, and incorporating fluorine/methyl groups or *N*-methyl protection to block metabolic soft spots. These approaches are expected to improve the pharmacokinetic profile of DIQ01 and facilitate its further development as a novel anticancer agent.

## 5 Conclusion

Our study highlights the potential of KHSRP as a therapeutic target in cancer. DIQ01 binds to the KH3 and KH4 domains of KHSRP, modulating mRNA biological processes (such as PLK1), while influencing an oncogenic post-translational modification of KHSRP. These dual actions contribute to broad anti-cancer effects in colorectal tumor models. Our findings reinforce the increasing recognition of RBPs as viable cancer drug targets. DIQ01 serves as a proof-of-principle, demonstrating that small molecules can selectively inhibit RBP function and alter cancer cell behavior, providing a basis for future RBP-targeted therapeutic development.

## Author contributions

Shuang Chen designed and conducted experiments, and wrote the manuscript. Ningjing Zhang, and Zhen Tang contributed to molecular biology experiments. Yuxiao Zhang and Ningjing Zhang offered support to animal experiments. Jiebin Fang provided molecular docking and assisted in target identification. Dashan Zhang, Yalin Yu, Mengde Xia, Yan Yi, and Xinyi Tang coordinated the experiments. Zhongjun Ma and Wanjing Ding conceived the idea and supported the project. All authors discussed the results and commented on the manuscript.

## Conflicts of interest

The authors declare no conflicts of interest.

## Appendix A Supporting information

The following is the Supporting Information to this article:
