# Supplementary material for: A novel small molecule inhibitor targeting KHSRP methylation suppresses colon cancer progression
Source: Acta Pharm Sin B. 2026 Jun 18;16(8):5237–58. doi: 10.1016/j.apsb.2026.06.031 (PMC13464083; doi:10.1016/j.apsb.2026.06.031)

Supporting Information for

Original article

A novel small molecule inhibitor targeting KHSRP methylation suppresses colon cancer progression

Shuang Chen[a,b], Ningjing Zhang[b], Yuxiao Zhang[b], Jiebin Fang[a,b], Zhen Tang[c], Dashan Zhang[d], Xinyi Tang[b], Yalin Yu[b], Mengde Xia[a,b], Yan Yi[b], Wanjing Ding[b,*], Zhongjun Ma[a,b,*]

[a]*Hainan Institute, Zhejiang University, Sanya 572025, China*

[b]*Institute of Marine Biology and Pharmacology, Ocean College, Zhejiang University, Zhoushan 316021, China*

[c]*School of Life Sciences, Westlake University, Hangzhou 310000, China*

[d]*Center of Synthetic Biology and Integrated Bioengineering, Westlake University, Hangzhou 310000, China*

Received 10 June 2025; received in revised form 2 September 2025; accepted 20 May 2026

*Corresponding authors.

E-mail addresses: mazj@zju.edu.cn (Zhongjun Ma), wading@zju.edu.cn (Wanjing Ding).

Supporting figures

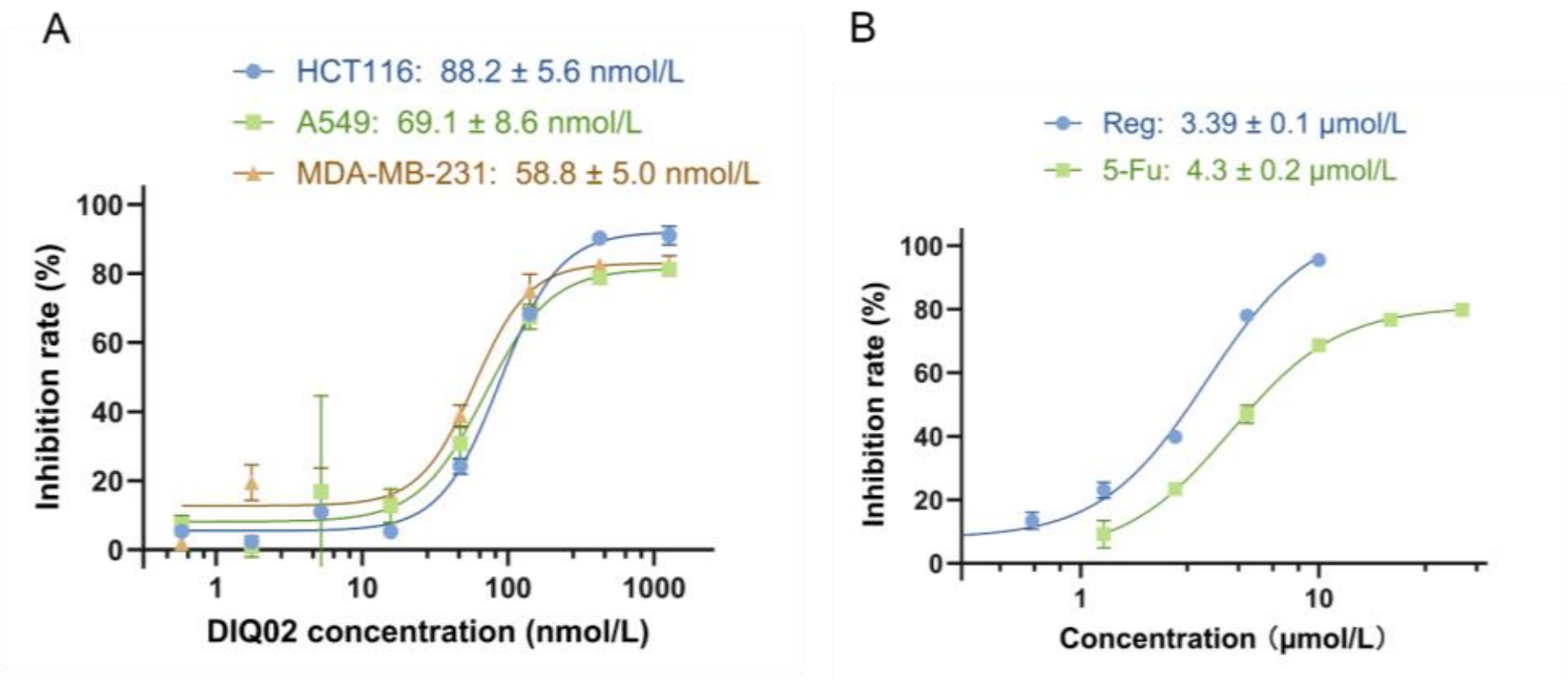

Figure S1 (A) Dose-response curves demonstrating antiproliferative effects of DIQ02 against three human cancer cell lines (HCT116, A549, and MDA-MB-231), assessed by the SRB assay after 72 h of treatment. (B) Dose–response curves showing the antiproliferative effects of 5-Fu and regorafenib on HCT116 cells, assessed by the SRB assay after 72 h of treatment. (A, B) Data are presented as the mean ± SD, *n* = 3.

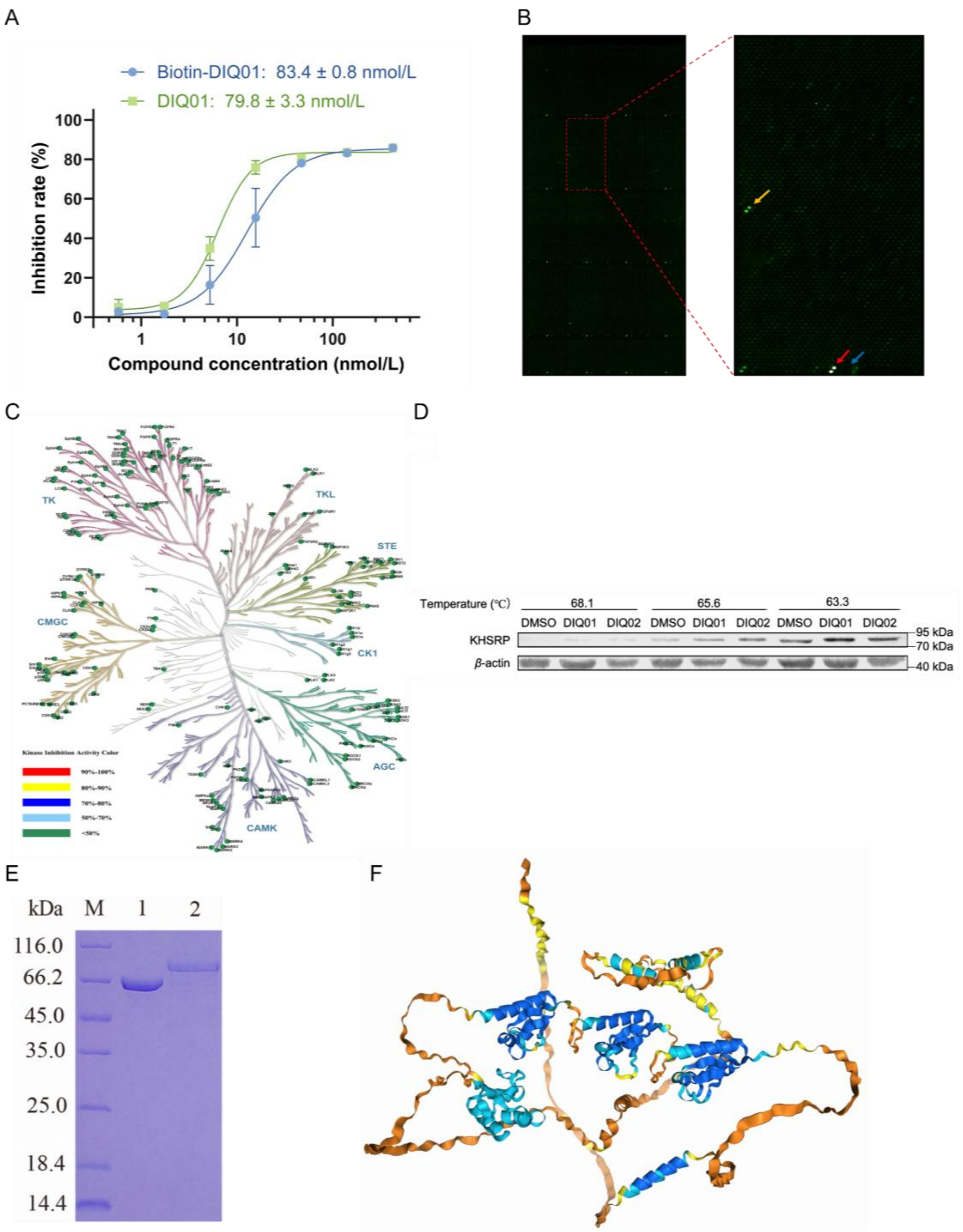

Figure S2 (A) Dose–response curves showing the antiproliferative effects of Biotin-DIQ01 and DIQ01 on HCT116 cells. Data are presented as the mean ± SD, *n* = 3. (B) Representative microarray interaction assay results. Left: full scan of the DIQ sample microarray. Right: enlarged view of Block 6. Red arrows indicate positive controls (Biotin-BSA), and blue arrows indicate negative controls (BSA). All control spots performed as expected, indicating the assay quality was acceptable. (C) Kinase profiling of DIQ01 at 200 nmol/L. (D) CETSA assays confirmed the binding of DIQ01 and DIQ02 to KHSRP in HCT116 cells. *β*-actin was used as a loading control. (E) SDS-

PAGE analysis of purified KHSRP protein. Lane M: protein molecular weight marker; Lane 1: BSA; Lane 2: purified target protein. (F) Predicted structural model of KHSRP generated by AlphaFold3.

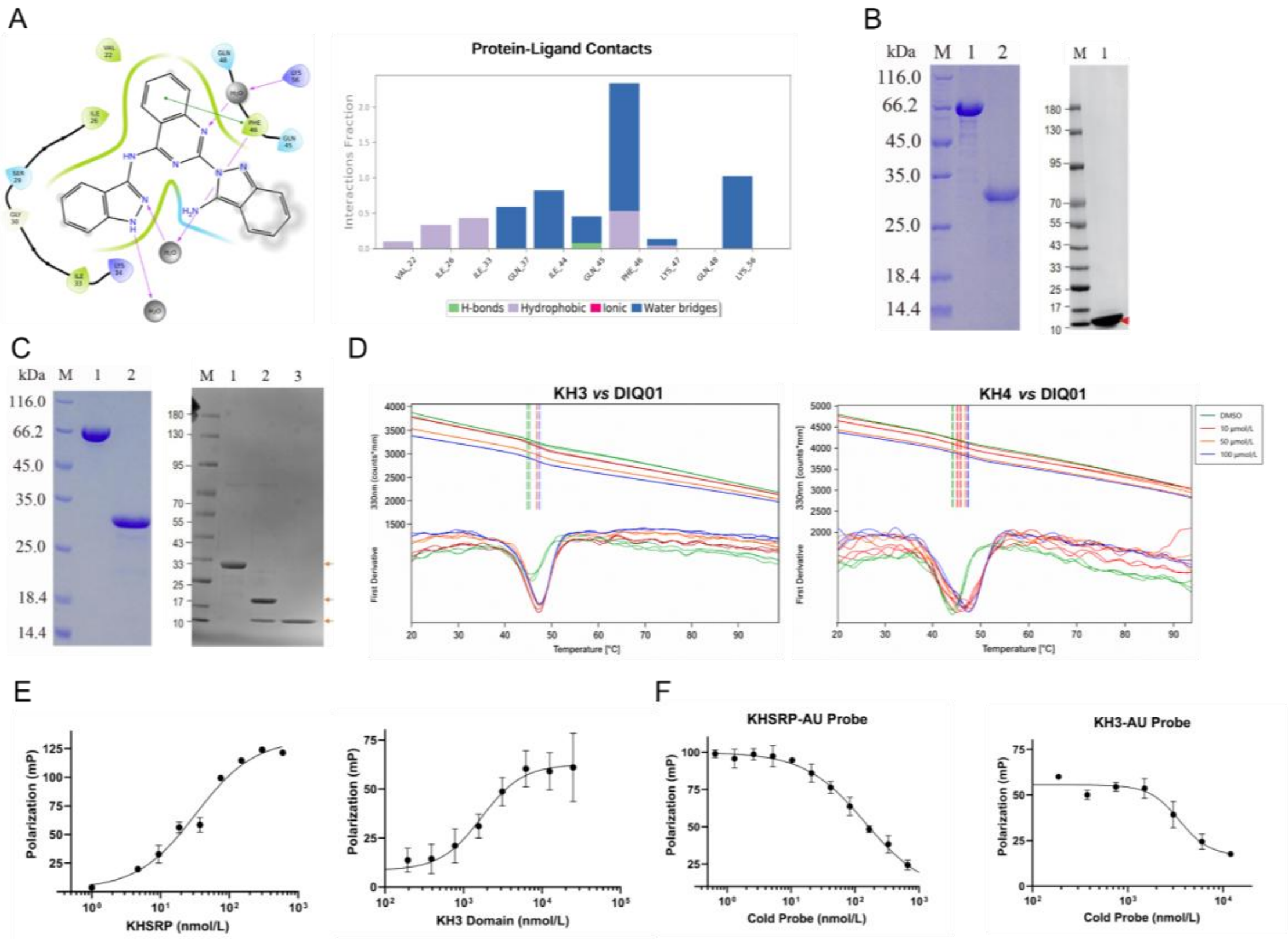

Figure S3 (A) Detailed interactions of DIQ01 with the KH3 domain and corresponding interaction fractions. Key residues in KH3 domain: Gln37, Ile44, Phe46, and Lys56, corresponding to residues in the full-length protein: Gln349, Ile356, Phe358, and Lys368. (B) SDS-PAGE analysis of purified proteins. Lane M: protein molecular weight marker; Lane 1: BSA; Lane 2: sumo-KH3 protein (left). Lane M: protein molecular weight marker; Lane 1: KH3 protein (right, after enzymatic cleavage). (C) SDS-PAGE analysis of purified proteins. Lane M: protein molecular weight marker; Lane 1: BSA; Lane 2: sumo-KH4 protein (left). Lane M: protein molecular weight marker; Lane 1: sumo-KH4 protein; Lane 2: sumo protein4; Lane 3: KH4 protein (right, after enzymatic cleavage). (D) Nano-DSF showing the binding between DIQ01 and the KH3 domain truncation (left) and the KH4 domain truncation (right). (E) Titration of KHSRP protein with 10 nmol/L AU probe (left), and titration of KH3 protein with 60 nmol/L AU probe (right). (F) Unlabeled Cold probe efficiently displaces tagged AU

probe bound to KHSRP (75 nmol/L) (left), or KH3 (2 μmol/L) in a dose-dependent manner (right). Error bars in (E) and (F) represent mean ± SD, n = 3.

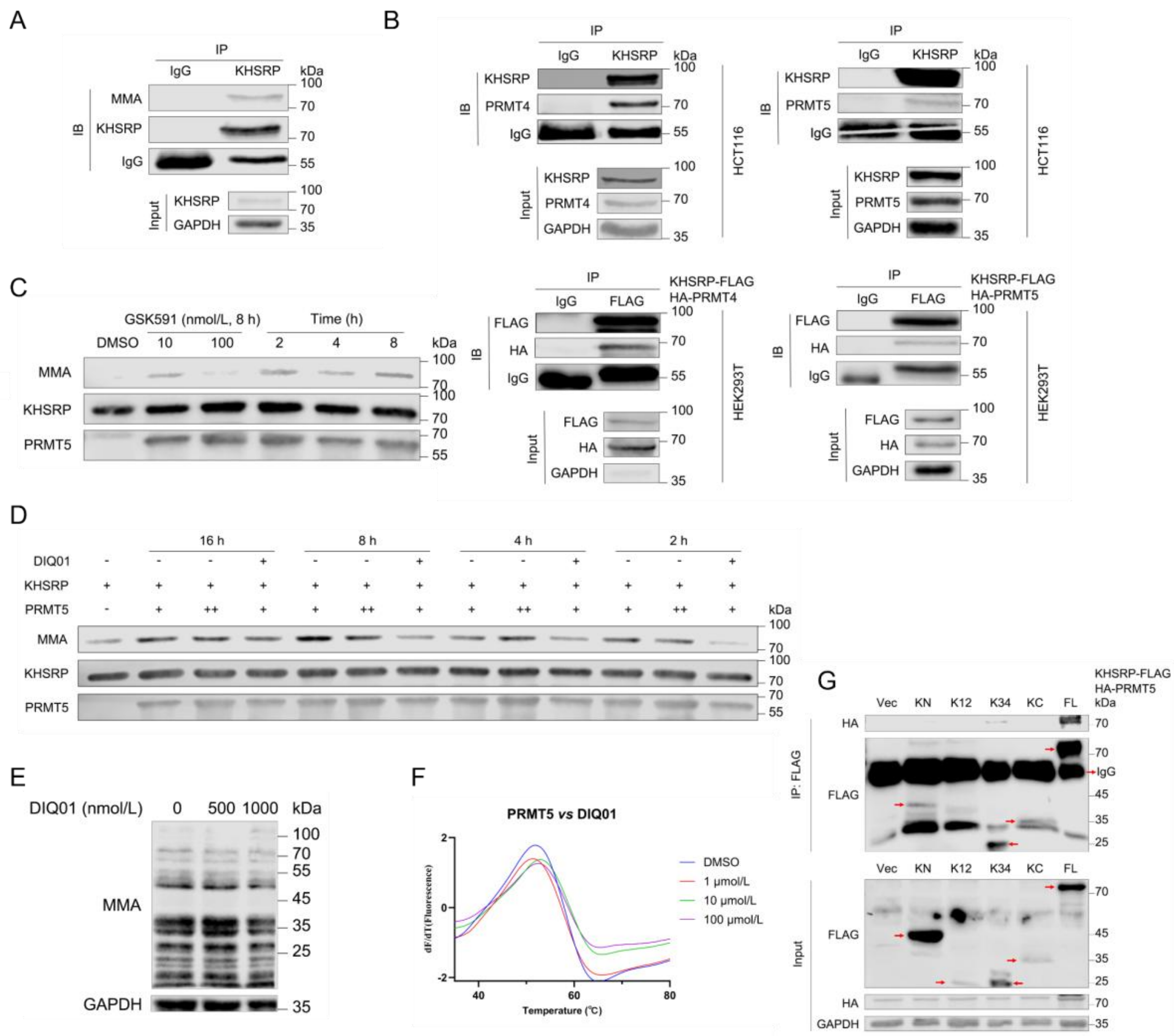

Figure S4 (A) Immunoprecipitation (IP) of endogenous KHSRP from HCT116 cells followed by Western blot using a pan-monomethylarginine (MMA) antibody confirms that KHSRP is arginine-methylated. (B) Co-IP assays demonstrating that both endogenous KHSRP in HCT116 cells and exogenously expressed FLAG-tagged KHSRP in HEK293T cells interact with PRMT4 and PRMT5. (C, D) *In vitro* PRMT5-mediated methylation of KHSRP: pharmacologic and kinetic controls. Reactions were performed as described in Methods and analyzed by Western blot with anti-MMA to detect KHSRP arginine methylation; total KHSRP and PRMT5 serve as loading/enzyme controls. (C) GSK591 (10–100 nmol/L, 8 h) suppressed KHSRP methylation, whereas vehicle controls displayed time-dependent methylation (2–8 h). (D) Time-course analysis (2–16 h) of PRMT5-mediated KHSRP methylation with or without DIQ01 (2 μmol/L). PRMT5 enzyme loads were tested (+ = 0.3 μg; ++ = 0.6

μg). The results show time- and enzyme-dependent methylation, which was effectively suppressed by DIQ01. (E) Western blot analysis of whole-cell lysates from HCT116 cells treated with increasing concentrations of DIQ01 (0, 500, and 1000 nmol/L). (F) DSF analysis of recombinant PRMT5 with DIQ01 (1–100 μmol/L) or DMSO. The dF/dT curves overlapped, showing no reproducible, concentration-dependent Tm shift; representative of ≥ 3 independent experiments. (G) Mapping of the PRMT5-interacting region on KHSRP using a panel of FLAG-tagged truncation mutants expressed in HEK293T cells. Co-immunoprecipitation was performed with anti-FLAG antibody, and HA-tagged PRMT5 interaction was detected by immunoblotting.

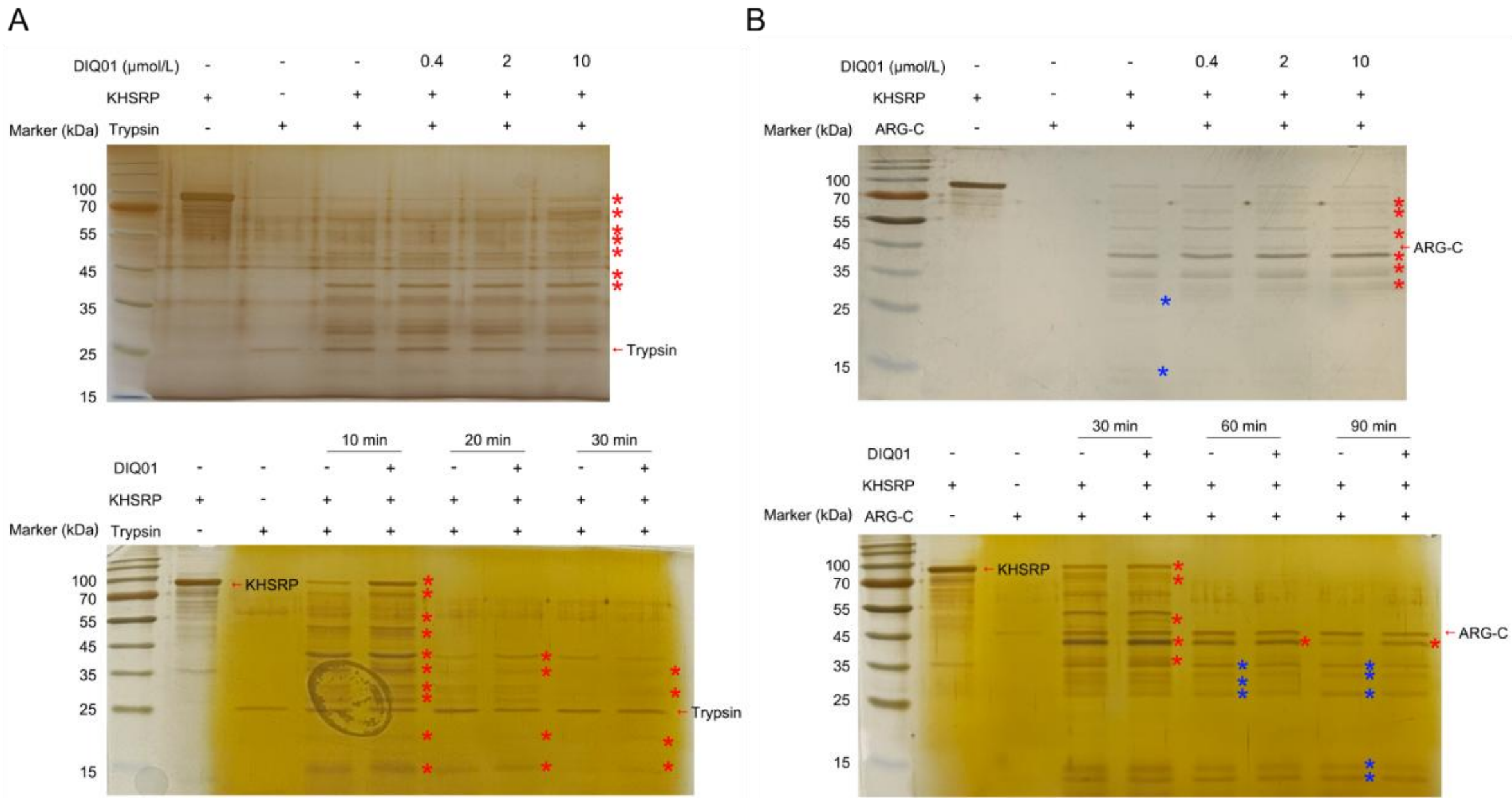

Figure S5 DIQ01 induces conformational remodeling of KHSRP revealed by limited proteolysis (LiP) and silver staining. (A) Trypsin-mediated limited proteolysis of recombinant full-length KHSRP. Top: DIQ01 dose-response (0–10 μmol/L) at a fixed digestion time of 10 min, with appropriate controls (substrate only, enzyme only, vehicle). Bottom: time course (10, 20, and 30 min) with DIQ01 (+) *versus* vehicle (−). Red asterisks denote DIQ01-dependent fragment bands indicative of altered protease accessibility; arrows mark the full-length KHSRP and trypsin bands. (B) Arg-C-mediated limited proteolysis of recombinant full-length KHSRP. Top: DIQ01 concentration-response (0–10 μmol/L) at a fixed digestion time of 30 min, with appropriate controls (substrate only, enzyme only, vehicle). Bottom: time course at 2 μmol/L DIQ01 (30, 60, and 90 min) *versus* vehicle. Red asterisks denote DIQ01-dependent fragment bands indicative of altered protease accessibility, while blue

asterisks denote fragments preferentially enriched in the vehicle condition. Arrows mark the full-length KHSRP and Arg-C bands.

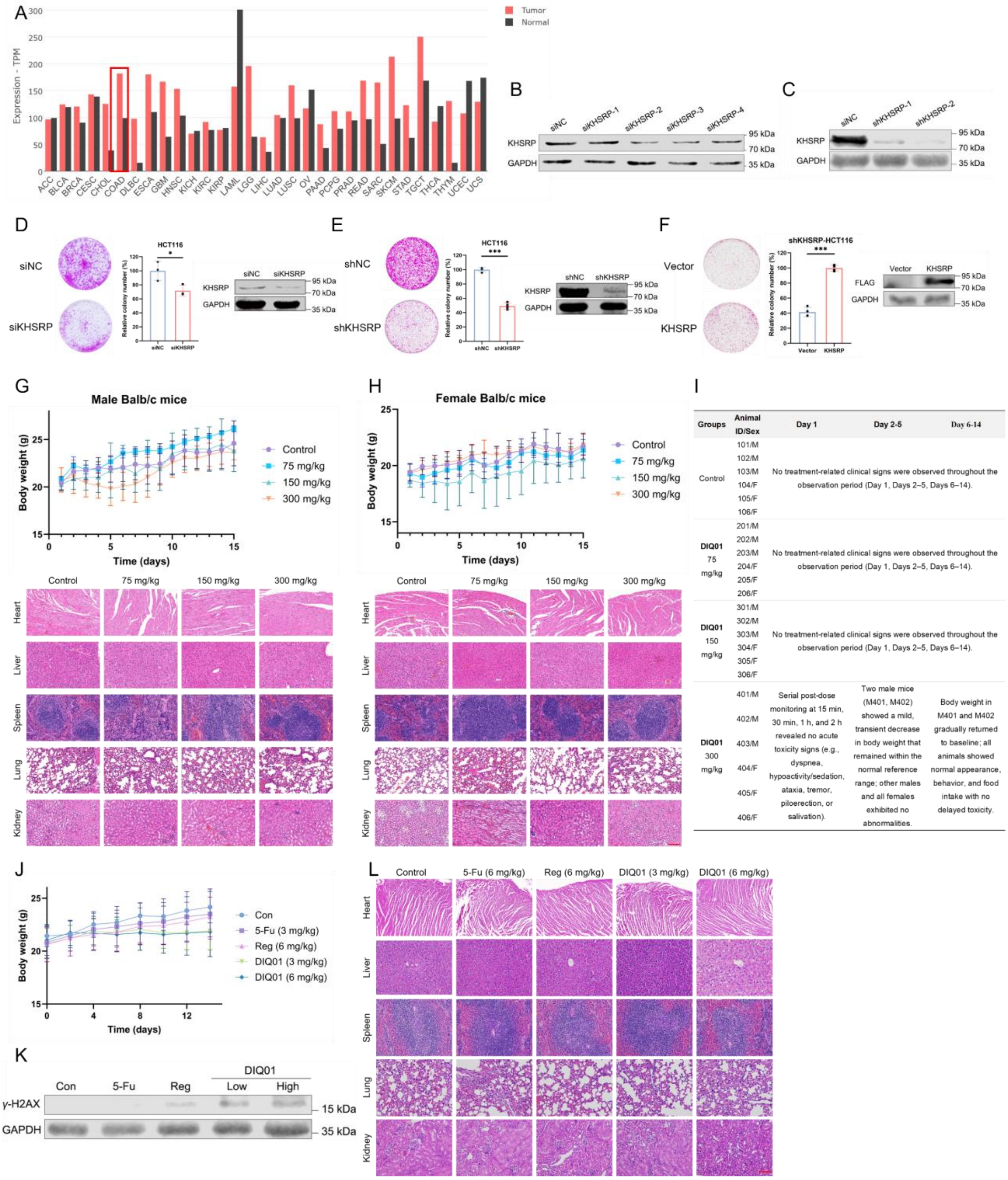

Figure S6 (A) Transcript-level expression of KHSRP across various cancer types analyzed *via* the GEPIA database. Colon adenocarcinoma (COAD) is marked in red, demonstrating higher mRNA levels of KHSRP in tumor tissues. (B) The expression of KHSRP was knocked down using four different siRNAs (siKHSRP-1, siKHSRP-2,

siKHSRP-3, and siKHSRP-4). GAPDH was used as the loading control. siKHSRP-3 demonstrated the most effective knockdown and was selected for further experiments. (C) The expression of KHSRP was stably knocked down using different shRNAs (shKHSRP-1, shKHSRP-2). GAPDH was used as the loading control. shKHSRP-2 demonstrated the most effective knockdown and was selected for subsequent experiments. (D) Representative colony formation assay of HCT116 cells subjected to transient knockdown of KHSRP using siRNA or a siNC. Cells were cultured for five days, stained with SRB, and cell viability was quantified by optical density (OD) measurement. (E) Representative colony formation assay of HCT116 cells with stable knockdown of KHSRP using shRNA or a non-targeting shRNA control (shNC). Cells were cultured for five days, stained with SRB, and OD values were measured to assess cell viability. (F) Rescue experiment in HCT116 cells with stable KHSRP knockdown. Cells were transfected with either a KHSRP expression construct or an empty vector as a control. Cells were cultured for five days, stained with SRB, and OD values were measured to assess cell viability. The data represent mean ± SD (n = 3). Statistical significance, *$P < 0.05$, ***$P < 0.001$. (G) Male mice (n = 3 per dose): body-weight trajectories after a single oral gavage of vehicle, 75, 150, or 300 mg/kg DIQ01. Points show mean ± SEM. Representative H&E-stained sections of heart, liver, spleen, lung, and kidney on Day 14 (400×) show no treatment-related lesions across doses. (H) Female mice (n = 3 per dose): body-weight trajectories (mean ± SEM) at the same dose levels. Representative H&E-stained sections of heart, liver, spleen, lung, and kidney on Day 14 (400×) show no treatment-related lesions. (I) Clinical-observation summary table. Serial post-dose monitoring at 15, 30, 60, and 120 min and daily checks revealed no treatment-related clinical signs in control, 75 and 150 mg/kg groups. (J) Body weight changes in mice during the treatment period. No significant differences were observed between the DIQ01-treated groups and the vehicle control group. (K) Western blot analysis of γ-H2AX levels in tumor tissues from the five treatment groups. (L) H&E staining of major organs (heart, liver, spleen, lung, and kidney, 400×) from mice in each treatment group.

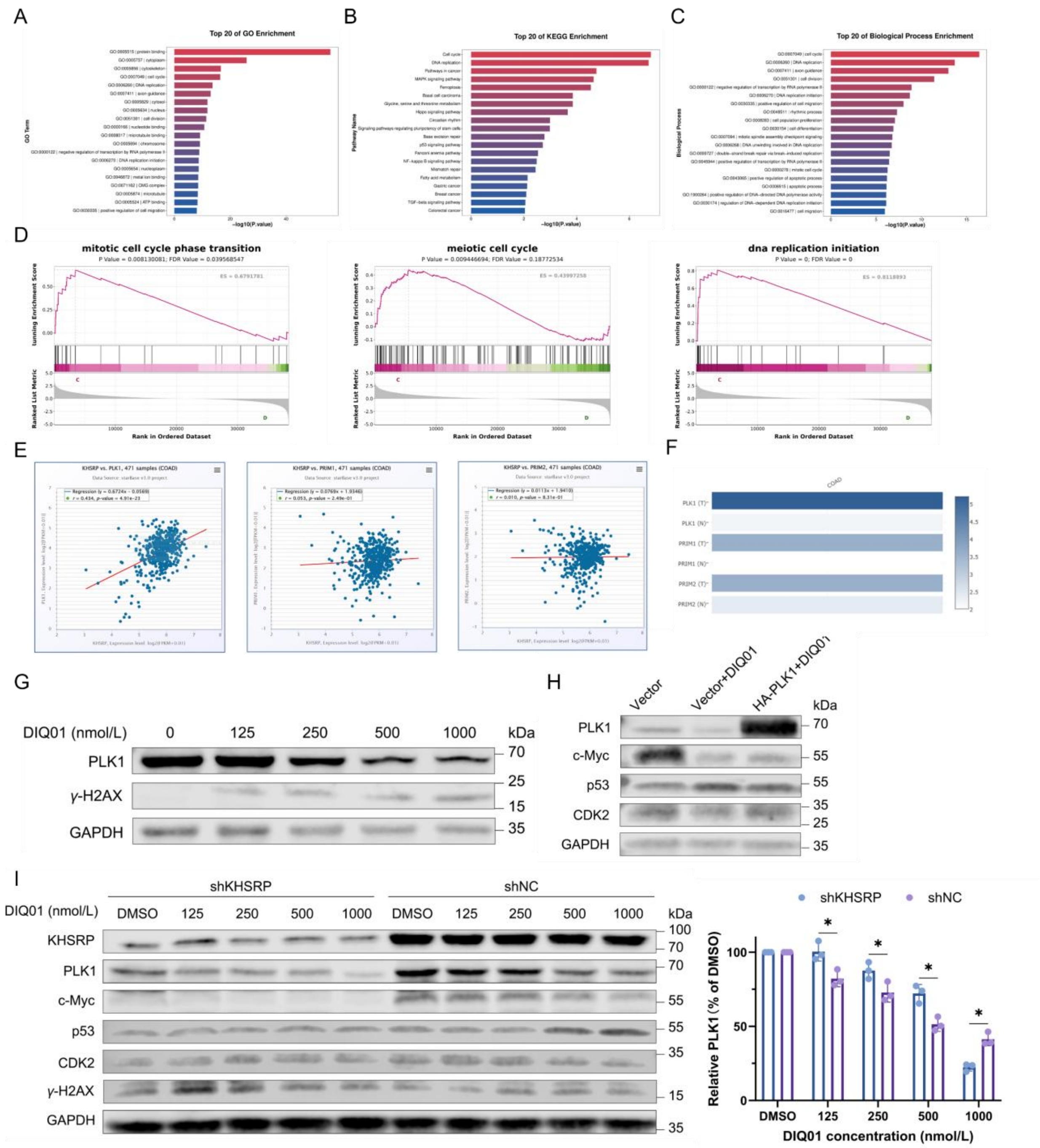

Figure S7 (A) Top 20 GO enrichment terms based on RNA-seq data from DIQ01-treated HCT116 cells. (B) Top 20 KEGG pathway enrichment results derived from the same RNA-seq dataset. (C) Top 20 enriched biological processes identified in DIQ01-treated cells based on GO biological process terms. (D) GSEA plots showing negative enrichment of DNA replication-related gene sets in the DIQ01 treatment group compared to DMSO. (E) Correlation analysis of *PLK1*, *PRIM1*, and *PRIM2* with *KHSRP* expression in colon cancer samples using the starBase database. (F) Expression levels of PLK1, PRIM1, and PRIM2 in normal tissues and colon cancer tissues,

analyzed *via* the GEPIA database. (G) Western blot analysis of PLK1 and γ-H2AX levels in DIQ01-treated HCT116 cells, with GAPDH as the loading control. (H) PLK1 overexpression partially reverses DIQ01-induced signaling changes in HCT116 cells. HCT116 cells were transfected with PLK1 or empty vector and analyzed by Western blot after treatment with DIQ01 (500 nmol/L), as indicated (lanes: Vector, Vector+DIQ01, HA-PLK1+DIQ01). Blots for PLK1, c-Myc, p53, and CDK2 are shown, with GAPDH as a loading control. (I) Left: Immunoblots of HCT116 cells with stable KHSRP knockdown (shKHSRP) or control (shNC) treated with DIQ01. Proteins probed: KHSRP, PLK1, c-Myc, p53, CDK2, γ-H2AX; GAPDH as loading control. Right: Quantification of PLK1 levels (GAPDH-normalized, relative to DMSO control). Data: mean ± SD, $n = 3$; $*P < 0.05$.

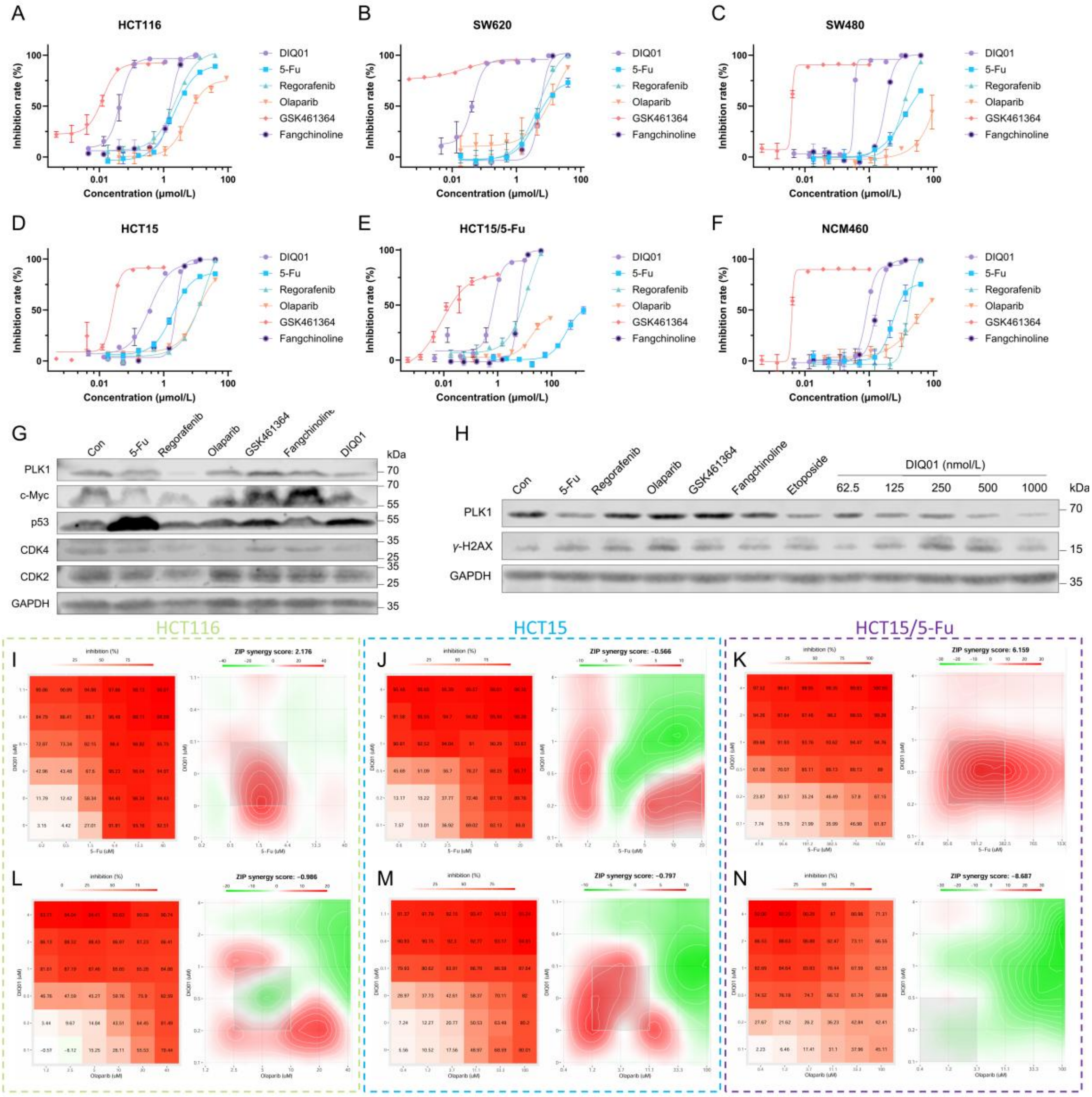

Figure S8 (A–F) Cell viability assays showing the concentration-dependent inhibitory effects of DIQ01, 5-fluorouracil (5-Fu), regorafenib, olaparib, the PLK1 inhibitor GSK461364, and fangchinoline in (A) HCT116, (B) SW620, (C) SW480, (D) HCT15, (E) 5-Fu-resistant HCT15/5-Fu, and (F) non-malignant colonic epithelial NCM460 cells. Cells were treated with the indicated compounds for 72 h, and inhibition rates (%) were determined. Data are presented as mean ± SD from three independent experiments, and IC_{50} values were derived from nonlinear regression analysis. (G) Immunoblot analysis of PLK1, c-Myc, p53, CDK4 and CDK2 after 24 h treatment with DIQ01 (500 nmol/L), 5-Fu (10 μmol/L), regorafenib (10 μmol/L), GSK461364 (50 nmol/L), olaparib (10 μmol/L) or fangchinoline (10 μmol/L). (H) Immunoblots collected after 8 h treatment at the same concentrations to capture the early DNA-damage signal before γ-H2AX attenuation; a DIQ01 dose series (62.5–1000 nmol/L) is shown, together with

single-dose comparators and etoposide as a positive control for γ-H2AX. GAPDH served as the loading control. Blots are representative of at least three independent experiments. (I–N) DIQ01 in combination with 5-Fu or olaparib across CRC cell lines. Six-by-six dose-response matrices (SRB readout, % inhibition; DIQ01 titrated 0.1–4 μmol/L) were analyzed in SynergyFinder using the ZIP model with baseline correction. For each condition, the left heatmap shows observed inhibition and the right map shows ZIP synergy scores (red = synergy; green = antagonism). (I–K) DIQ01 + 5-Fu in HCT116, HCT15, and HCT15/5-Fu, respectively: global ZIP +2.176 (MSA 6.43), −0.566 (MSA 1.56), and +6.159 (MSA 10.99). (L–N) DIQ01 + olaparib in HCT116, HCT15, and HCT15/5-Fu: global ZIP −0.797 (MSA 3.10), −0.986 (MSA 2.16), and −8.687 (MSA −0.73). Partner-drug dose ranges are indicated on the axes.

Supporting tables

Table S1 Primer sequences for qPCR.

Gene (human)	Primer	Sequence (5′–3′)	Company
KHSRP	Forward	GCCGCTTACTACGGACAGAC	BGI tech
	Reverse	ACATTCATTCGATTCATTGAGC	
PLK1	Forward	TCTGGAGGTGAAAATAGGGGA	BGI tech
	Reverse	TAGGCAAGAAGTCTCAAAAGGTG	
GAPDH	Forward	CAATGACCCCTTCATTGACC	BGI tech
	Reverse	TTGATTTTGGAGGGATCTCG	

Table S2 Primer sequences for truncation-based construction.

Name	Primer	Sequence (5′–3′)	Company
KN	Forward	CTAGCTAGCTAGATGAGCGACTACT	BGI tech
	Reverse	CGGGATCCCGTTCTCGCAGAATGTC	
K12	Forward	CTAGCTAGCTAGAGCATGACCGAAG	BGI tech
	Reverse	CGGGATCCCGCCTTTCTCGCAGAAT	
K34	Forward	CTAGCTAGCTAGATCGGAGGCGGTA	BGI tech
	Reverse	CGGGATCCCGTGGATTGAATGGACC	
KC	Forward	CTAGCTAGCTAGGAGAAGATTGAGGGTC	BGI tech
	Reverse	CGGGATCCCGTTGCCGATAGTACTC	

Table S3 siRNA sequences.

Name		Sequence (5′–3′)	Company
siRNA-KHSRP-1	Sense	CCGAGAAGAUUGCUCAUAUTT	Genepharma
	Antisense	AUAUGAGCAAUCUUCUCGGTT	
siRNA-KHSRP-2	Sense	GAGUGAAGAUGAUCUUAAUTT	Genepharma
	Antisense	AUUAAGAUCAUCUUCACUCTT	
siRNA-KHSRP-3	Sense	GAGGAGGUGAACAAAUUAATT	Genepharma
	Antisense	UUAAUUUGUUCACCUCCUCTT	
siRNA-KHSRP-4	Sense	GAUCAACCGGAGAGCAAGATT	Genepharma
	Antisense	UCUUGCUCUCCGGUUGAUCTT	

Table S4 shRNA sequences.

Name	Fluorescent Protein	Sequence (5′–3′)	Company
shRNA-KHSRP-1	RFP	GACTTCAATGACAGAAGAGT	Genepharma
shRNA-KHSRP-2	RFP	CGCCTACTACTCACACTACTA	Genepharma
shNC	RFP	TTCTCCGAACGTGTCACGT	Genepharma

Table S5 PK parameters of DIQ01 in mice (i.v. 3 mg/kg; *p.o.* 25 mg/kg).

Route	$AUC_{0\text{–}t}$	$AUC_{0\text{–inf}}$	CL	V_z	V_{ss}	$t_{1/2}$	T_{max}	C_{max}	F
	(h·ng/mL)	(h·ng/mL)	(mL/min/kg)	(L/kg)	(L/kg)	(h)	(h)	(ng/mL)	(%)
i.v., 3 mg/kg	9951	9981	5.05	1.40	0.82	3.17	NA	NA	NA
p.o., 25 mg/kg	2117	2128	NA	NA	NA	3.17	1.67	530	2.56

- Values are means (n = 3 mice per group).
- NA, not applicable (*e.g.*, CL, *V*z, and *V*ss are reported for i.v. only).
- F (%) = ($AUC_{0\text{–inf}}$, *p.o.* × intravenous dose) / ($AUC_{0\text{–inf}}$, i.v. × oral dose) × 100.
- Plasma: noncompartmental analysis (Phoenix WinNonlin v8.3). Detailed per-animal pharmacokinetic parameters, AUC% extrapolated values, and plasma concentration–time data are provided in Table S5 continued.

Table S5 continued Individual pharmacokinetic parameters of DIQ01 after intravenous administration in mice (i.v., 3 mg/kg).

Parameter	Mouse #19	Mouse #20	Mouse #21	Mean	SD	CV (%)
No. of points used for $t_{1/2}$	3	3	3	NA	NA	NA
$t_{1/2}$ (h)	3.03	2.87	3.61	3.17	0.39	12.18
C_0 (ng/mL)	3865.84	4714.19	3154.16	3911.4	781.01	19.97
$AUC_{0\text{–}t}$ (h ng/mL)	9756.54	11106.77	8990.51	9951.27	1071.48	10.77
$AUC_{0\text{–inf}}$ (h ng/mL)	9778.92	11125.29	9039.88	9981.36	1057.34	10.59
AUC% extrapolated	0.23	0.17	0.55	0.31	0.2	64.86
V_z (L/kg)	1.34	1.12	1.73	1.4	0.31	22.08
V_{ss} (L/kg)	0.81	0.68	0.97	0.82	0.15	17.86
CL (mL/min/kg)	5.11	4.49	5.53	5.05	0.52	10.34
$MRT_{0\text{–inf}}$ (h)	2.65	2.51	2.92	2.69	0.21	7.77
Last time point for $AUC_{0\text{–}t}$ (h)	24	24	24	NA	NA	NA
Time points for $t_{1/2}$ (h)	4, 8, 24	4, 8, 24	4, 8, 24	NA	NA	NA
R^2	0.955	0.96	0.955	NA	NA	NA

Table S5 continued Individual pharmacokinetic parameters of DIQ01 after oral administration in mice (*p.o.*, 25 mg/kg).

Parameter	Mouse #22	Mouse #23	Mouse #24	Mean	SD	CV (%)
No. of points used for $t_{1/2}$	2	3	2	NA	NA	NA
$t_{1/2}$ (h)	2.79	3.18	3.54	3.17	0.38	11.86
T_{max} (h)	2	2	1	1.67	0.58	34.64
C_{max} (ng/mL)	487	721	382	530	173.54	32.74
$AUC_{0–t}$ (h ng/mL)	2071.28	2285.87	1993.85	2117	151.28	7.15
$AUC_{0–inf}$ (h ng/mL)	2077.15	2293.16	2012.22	2127.51	147.09	6.91
AUC% extrapolated	0.28	0.32	0.91	0.5	0.35	70.2
$MRT_{0–inf}$ (h)	4.31	3.07	4.86	4.08	0.92	22.52
$AUC_{0–inf}$/D (h kg ng/mL/mg)	83.09	91.73	80.49	85.1	5.88	6.91
F (%)	2.5	2.76	2.42	2.56	0.18	6.91
Last time point for $AUC_{0–t}$ (h)	24	24	24	NA	NA	NA
Time points for $t_{1/2}$ (h)	8, 24	4, 8, 24	8, 24	NA	NA	NA
R^2	1	0.906	1	NA	NA	NA

Table S5 continued Plasma concentration–time data of DIQ01 after intravenous administration in mice (i.v., 3 mg/kg).

Time (h)	Mouse #19	Mouse #20	Mouse #21	Mean	SD	CV (%)
0.08	3480	4190	2980	3550	608.03	17.13
0.25	2820	3310	2660	2930	338.67	11.56
0.5	2780	3210	2680	2890	281.6	9.74
1	2170	3050	2230	2483.33	491.66	19.8
2	1840	1830	1500	1723.33	193.48	11.23
4	701	785	593	693	96.25	13.89
8	99.9	107	115	107.3	7.55	7.04
24	5.12	4.47	9.49	6.36	2.73	42.93

Table S5 continued Plasma concentration–time data of DIQ01 after oral administration in mice (*p.o.*, 25 mg/kg).

Time (h)	Mouse #22	Mouse #23	Mouse #24	Mean	SD	CV (%)
0.25	92	42.6	227	120.53	95.45	79.19
0.5	184	108	300	197.33	96.69	49
1	476	450	382	436	48.54	11.13
2	487	721	382	530	173.54	32.74
4	32.7	202	36.1	90.27	96.78	107.21
8	78.2	19.7	82.8	60.23	35.18	58.4
24	1.46	1.59	3.6	2.22	1.2	54.12

Note: BLOQ, below the lower limit of quantification; LLOQ = 1 ng/mL. *F* was calculated based on $AUC_{0–inf}$. For cases with high AUC% extrapolated values, the total AUC may be less reliable and additional sampling would be needed for more accurate estimation of the elimination phase.

Table S6 IC_{50} values (μmol/L) of DIQ01 and comparators across five colorectal cancer cell lines and NCM460.

Compounds	HCT116	SW620	SW480	HCT15	HCT15/5-Fu	NCM460
DIQ01	0.065 ±0.009	0.044 ±0.004	0.335 ±0.031	0.366 ±0.032	0.671 ±0.074	0.818 ±0.047
5-Fu	4.338 ±0.140	4.090 ±0.291	13.28 ±5.146	1.904 ±0.079	>1530	5.258 ±0.513
Regorafenib	3.465 ±0.197	5.218 ±0.969	11.78 ±0.754	19.63 ±5.729	12.75 ±2.699	16.26 ±8.830
Olaparib	7.905 ±0.922	15.41 ±1.365	>40	11.61 ±1.678	>40	32.22 ±25.43
GSK461364	0.017 ±0.002	< 0.001	0.004 ±0.001	0.026 ±0.003	0.008 ±0.002	0.004 ±0.001
Fangchinoline	2.387 ±0.172	5.461 ±0.385	3.167 ±0.099	2.459 ±0.103	5.868 ±0.195	1.712 ±0.192

- Values are mean ± SD from n = 3 independent experiments.
- ">40" indicates no 50% inhibition at the highest tested concentration.
- HCT15/5-Fu is a 5-Fu-resistant HCT15 subline.
- Regorafenib (multikinase inhibitor), Olaparib (PARP inhibitor), GSK461364 (PLK1 inhibitor), Fangchinoline (reported KHSRP ligand).

1. General methods and synthesis of DIQ01, DIQ02 and Biotin-DIQ01

Commercial solvents and reagents used for this work were purchased from Shanghai Chemical Reagents Co., Ltd. in China, without further purification. Solvents used for HPLC were of HPLC grade from J&K Scientific Ltd. The ^{1}H NMR and ^{13}C NMR spectra of the target compounds in this study were obtained by the use of Bruker AM-600/400 NMR spectrometers, while HRESIMS was obtained using an Agilent 6230 TOF LC–MS mass spectrometer. Target compounds were purified using preparative HPLC on a Shimadzu high-performance liquid chromatography with an Agilent ODS column (10 μm, 4.6 mm × 250 mm). Column chromatography (CC) was performed using silica gel (100–200, 200–300 mesh, Qingdao Haiyang Chemical Co., Ltd.) and thin-layer chromatography (TLC) monitors used precoated silica gel GF254 plates from Qingdao Haiyang Chemical Co., Ltd. in China. The chemical shifts (δ) for ^{1}H and ^{13}C were measured in parts per million relative to the residual signals of the solvent, which was DMSO-d_6 at 2.50 ppm for ^{1}H NMR and 39.52 ppm for ^{13}C NMR. X-ray single-crystal diffraction of DIQ01 and DIQ02 was performed by Leyan (Shanghai, China) and Shanguan Technology (Guangzhou, China).

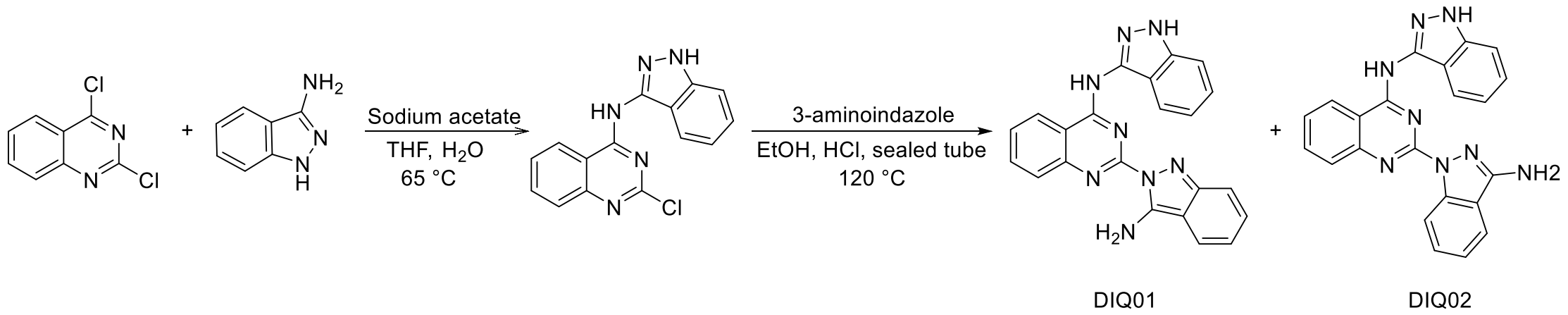

Step 1: 3-Aminoindazole (1.1 equivalents) and sodium acetate were mixed with 1 equivalent of 2,4-dichloroquinazoline at a concentration of 0.1 mmol in a 3:1 mixture of tetrahydrofuran and water (8 mL) and heated at 65 ℃ for 4 h. When the reaction was complete, as monitored by TLC, the solution was diluted with ethyl acetate. The layers

were separated, and the organic phase was washed three times with an equal volume of water and then dried over Na_2SO_4. The compound was recrystallized from a mixture of ethanol and water, filtered, and rinsed with cold ethanol to yield the pure product. The crude product was used directly in the next step.

Step 2: To a vessel containing the entire product from the previous step, ethanol (5 mL), hydrochloric acid (10 μL), and 3-aminoindazole (1 mmol) were added. The vessel was sealed and stirred for 12 h at 120 ℃. After the reaction, the temperature was reduced to room temperature, and the solvent was removed under reduced pressure. The mixture was dissolved in methanol, filtered through a 0.22 μm organic filter membrane, and purified by preparative high-performance liquid chromatography (HPLC) using an Agilent ODS column. The product was eluted using a two-solvent system: (A) trifluoroacetic acid (TFA)/H_2O (0.05% *v*/*v*) and (B) TFA/acetonitrile (ACN), with a 35% isocratic solvent B over 40 min.

2-(3-Amino-2*H*-indazol-2-yl)-*N*-(1*H*-indazol-3-yl)quinazolin-4-amine (DIQ01), yellow solid, yield: 31%. ^{1}H NMR (600 MHz, DMSO-d_6) δ 13.17 (s, 1H), 11.23 (s, 1H), 9.67 (s, 2H), 8.83 (d, *J* = 8.3 Hz, 1H), 8.16 (d, *J* = 8.3 Hz, 1H), 8.05 (ddd, *J* = 8.3, 6.8, 1.2 Hz, 1H), 8.0 (d, *J* = 8.3 Hz, 1H), 7.97 (d, *J* = 8.3 Hz, 1H), 7.78 (ddd, *J* = 8.3, 6.8, 1.0 Hz, 1H), 7.76 (ddd, *J* = 8.3, 6.8, 1.0 Hz, 1H), 7.65 (d, *J* = 8.6 Hz, 1H), 7.50 (ddd, *J* = 8.3, 6.8, 1.0 Hz, 1H), 7.47 (d, *J* = 8.5 Hz, 1H), 7.27 (t, *J* = 7.6 Hz, 1H), 7.20 (t, *J* = 7.6 Hz, 1H). ^{13}C NMR (150 MHz, DMSO-d_6) δ 158.98, 158.15, 150.81, 149.50, 149.33, 142.75, 140.90, 139.47, 135.14, 135.11, 127.44, 127.11, 126.81, 124.29, 123.48, 122.00, 120.57, 120.49, 116.10, 113.57, 111.65, 110.73. HRMS (ESI) *m*/*z* calcd. for $C_{22}H_{16}N_8$ [M + H]$^+$: 393.1571; found, 393.1573.

2-(3-Amino-1*H*-indazol-1-yl)-*N*-(1*H*-indazol-3-yl)quinazolin-4-amine (DIQ02), yellow solid, yield: 35%. ^{1}H NMR (600 MHz, DMSO-d_6) δ 13.43 (s, 1H), 11.96 (s, 1H), 8.71 (d, *J* = 8.4 Hz, 1H), 8.16 (d, *J* = 8.4 Hz, 1H), 8.04 (ddd, *J* = 8.4, 7.0, 1.2 Hz, 1H), 7.90 (d, *J* = 8.4 Hz, 1H), 7.74 (d, *J* = 8.4 Hz, 1H), 7.73 (d, *J* = 8.4 Hz, 1H), 7.71 (ddd, *J* = 8.4, 7.0, 1.2 Hz, 1H), 7.46 (ddd, *J* = 8.4, 7.0, 1.2 Hz, 1H), 7.20 (t, *J* = 7.7 Hz, 1H), 7.08-6.99 (m, 1H), 6.95 (d, *J* = 8.4 Hz, 1H), 6.81 (s, 2H), 6.70 (t, *J* = 7.7 Hz, 1H). ^{13}C NMR (150 MHz, DMSO-d_6) δ 161.25, 157.27, 154.70, 154.28, 147.55, 141.38, 140.09, 138.79, 138.47, 136.18, 129.00, 127.02, 126.19, 124.61, 124.14, 121.08, 120.84, 120.65, 117.42, 115.66, 111.12, 110.84. HRMS (ESI) *m*/*z* calcd. for $C_{22}H_{16}N_8$ [M + H]$^+$: 393.1571; found, 393.1568.

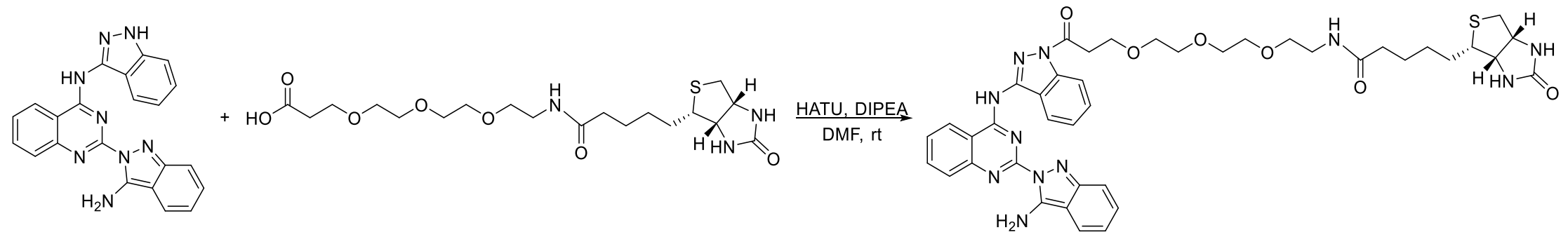

Step 3: A solution of 14-oxo-18-((3a*S*,4*S*,6a*R*)-2-oxohexahydro-1*H*-thieno[3,4-d]imidazol-4-yl)-4,7,10-trioxa-13-azaoctadecanoic acid (0.1 mmol) (1.0 eq) in DMF (4 mL) was treated with HATU (1.5 eq), DIPEA (2.0 eq) and DIQ01 (1.5 eq). The mixture was stirred at room temperature for 48 h. Solvent was then removed under reduced pressure and the residue was dissolved in DCM and washed with saturated aqueous solution of $NaHCO_3$, saturated aqueous NaCl and then dried over Na_2SO_4. After filtration and removal of the solvent a residue was obtained that was purified by preparative RP-HPLC using an Agilent ODS column. The product was eluted using a two-solvent system: (A) TFA/H_2O (0.05% *v*/*v*) and (B) TFA/methanol (MeOH), with a 40% to 70% gradient of solvent B over 10 min.

Biotin-DIQ01, yellow oil, yield: 78%. ^{1}H NMR (600 MHz, DMSO-d_6) δ 11.59 (s, 1H), 9.42 (s, 2H), 8.81 (d, J = 7.9 Hz, 1H), 8.49 (d, J = 8.6 Hz, 1H), 8.15-8.03 (m, 4H), 7.81–7.76 (m, 4H), 7.46 (t, J = 7.7 Hz, 1H), 7.41 (d, J = 8.5 Hz, 1H), 7.28 (s, 1H), 6.41 (s, 1H), 6.37 (s, 1H), 4.28 (dd, J = 7.9, 4.7 Hz, 1H), 4.10 (dd, J = 7.7, 4.4 Hz, 1H), 3.93-3.91 (m, 2H), 3.56 (t, J = 4.8 Hz, 2H), 3.53–3.46 (m, 4H), 3.46–3.43 (m, 2H), 3.42–3.39 (m, 2H), 3.31 (t, J = 6.0 Hz, 2H), 3.12 (q, J = 5.9 Hz, 2H), 3.05 (ddd, J = 8.6, 6.1, 4.3 Hz, 1H), 2.79 (dd, J = 12.4, 5.1 Hz, 1H), 2.56 (d, J = 12.4 Hz, 1H), 2.02 (t, J = 7.5 Hz, 2H), 1.61–1.53 (m, 1H), 1.47–1.41 (m, 3H), 1.31–1.20 (m, 2H). ^{13}C NMR (150 MHz, DMSO-d_6) δ 172.11, 171.05, 162.72, 158.25, 158.02, 150.67, 149.25, 142.75, 140.87, 139.54, 135.51, 130.83, 127.23, 127.18, 124.66, 124.46, 123.46, 123.27, 122.19, 120.44, 118.08, 115.18, 113.57, 111.62, 110.71, 69.78, 69.67, 69.61, 69.48, 69.09, 65.74, 61.03, 59.19, 55.41, 40.06, 39.49, 38.38, 35.05, 28.16, 28.01, 25.23. HRMS (ESI) *m*/*z* calcd. for $C_{41}H_{47}N_{11}O_6S$ $[M + H]^+$: 822.3504; found, 822.3503.

2. Crystallographic data, NMR, HRMS and HPLC spectra of DIQ01, DIQ02 and Biotin-DIQ01

2.1.Crystallographic data for DIQ01 and DIQ02

Crystal data of DIQ01: 3($C_{22}H_{16}N_8 \cdot C_2HF_3O_2$), M =1519.36, crystal size 0.2 × 0.08 × 0.06 mm^3, triclinic, space group P-1 (no. 2), a = 10.4640(5) Å, b = 15.6901(10) Å, c = 27.8657(15) Å, α = 74.696(4)°, β = 86.052(4)°, γ = 75.172(4)°, V = 4265.8(4) Å3, Z =

2, T = 218.0 K, μ(Cu Kα) = 0.795 mm^{-1}, $Dcalc$ = 1.183 g/cm^3, 37230 reflections measured (6.0° ≤ 2θ ≤ 136.51°), 15166 unique (R_{int} = 0.1057, R_{sigma} = 0.0707) which were used in all calculations. The final R_1 was 0.1359 (I > 2σ(I) and wR_2 was 0.2659 (all data). (deposition number: CCDC 2449697).

Crystal data of DIQ02: $C_{22}H_{16}N_8$, M =392.43, crystal size 0.15 × 0.1 × 0.08 mm^3, triclinic, space group P-1 (no. 2), a = 13.926(2) Å, b = 14.094(2) Å, c = 14.645(2) Å, α = 66.136(15)°, β = 80.518(13)°, γ = 66.368(15)°, V = 2408.3(7) Å3, Z = 4, T = 170.0 K, μ(Cu Kα) = 0.560 mm^{-1}, $Dcalc$ = 1.082 g/cm^3, 15469 reflections measured(6.6° ≤ 2θ ≤ 133.17°)), 8432 unique ($Rint$ = 0.1124, $Rsigma$ = 0.2196) which were used in all calculations. The final R_1 was 0.1078 (I > 2σ(I)) and wR_2 was 0.2988 (all data). (deposition number: CCDC 2449670).

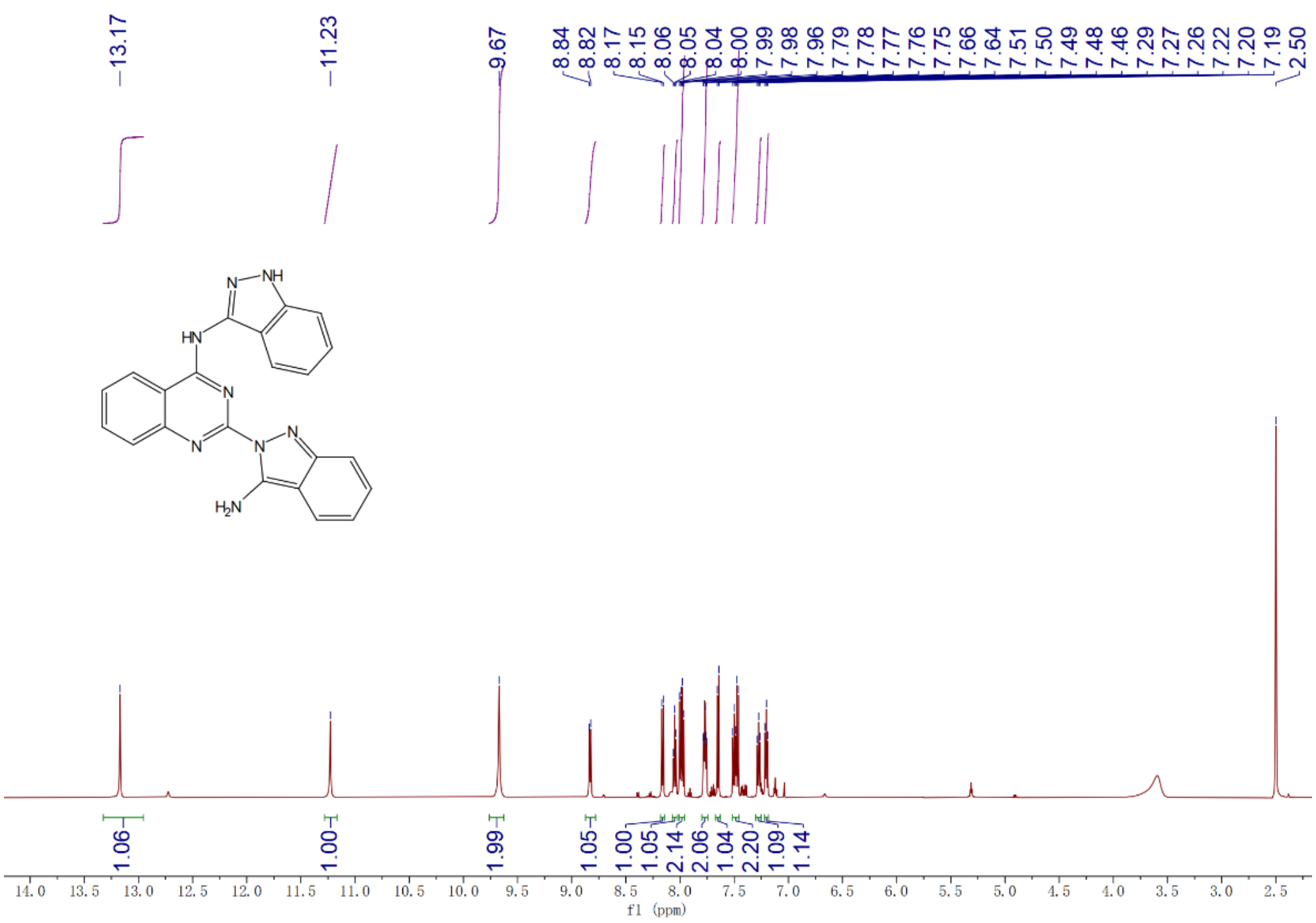

Figure S9 ^{1}H NMR spectrum of 2-(3-amino-2*H*-indazol-2-yl)-*N*-(1*H*-indazol-3-yl)quinazolin-4-amine (DIQ01).

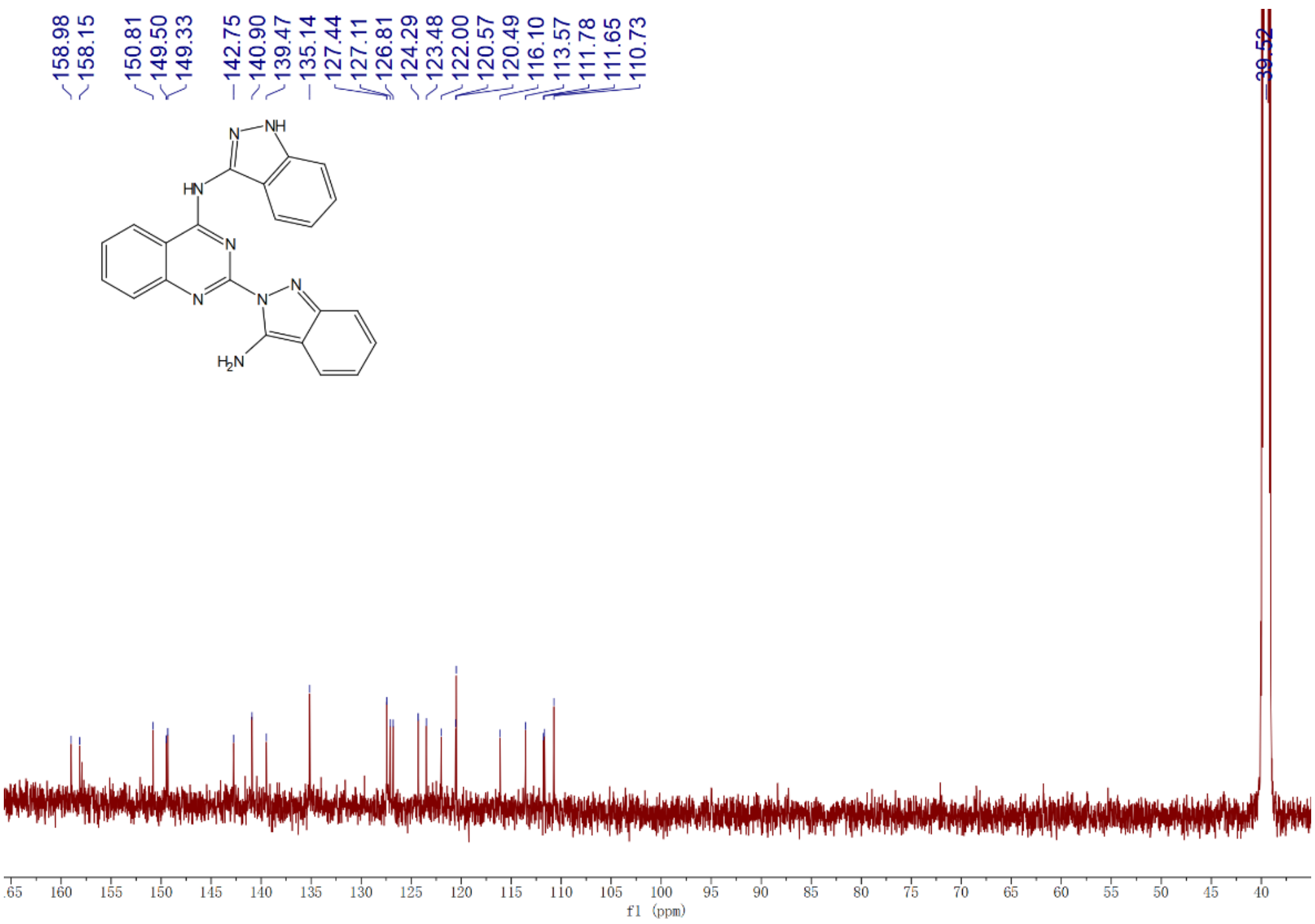

Figure S10 ^{13}C NMR spectra of 2-(3-amino-2*H*-indazol-2-yl)-*N*-(1*H*-indazol-3-yl)quinazolin-4-amine (DIQ01).

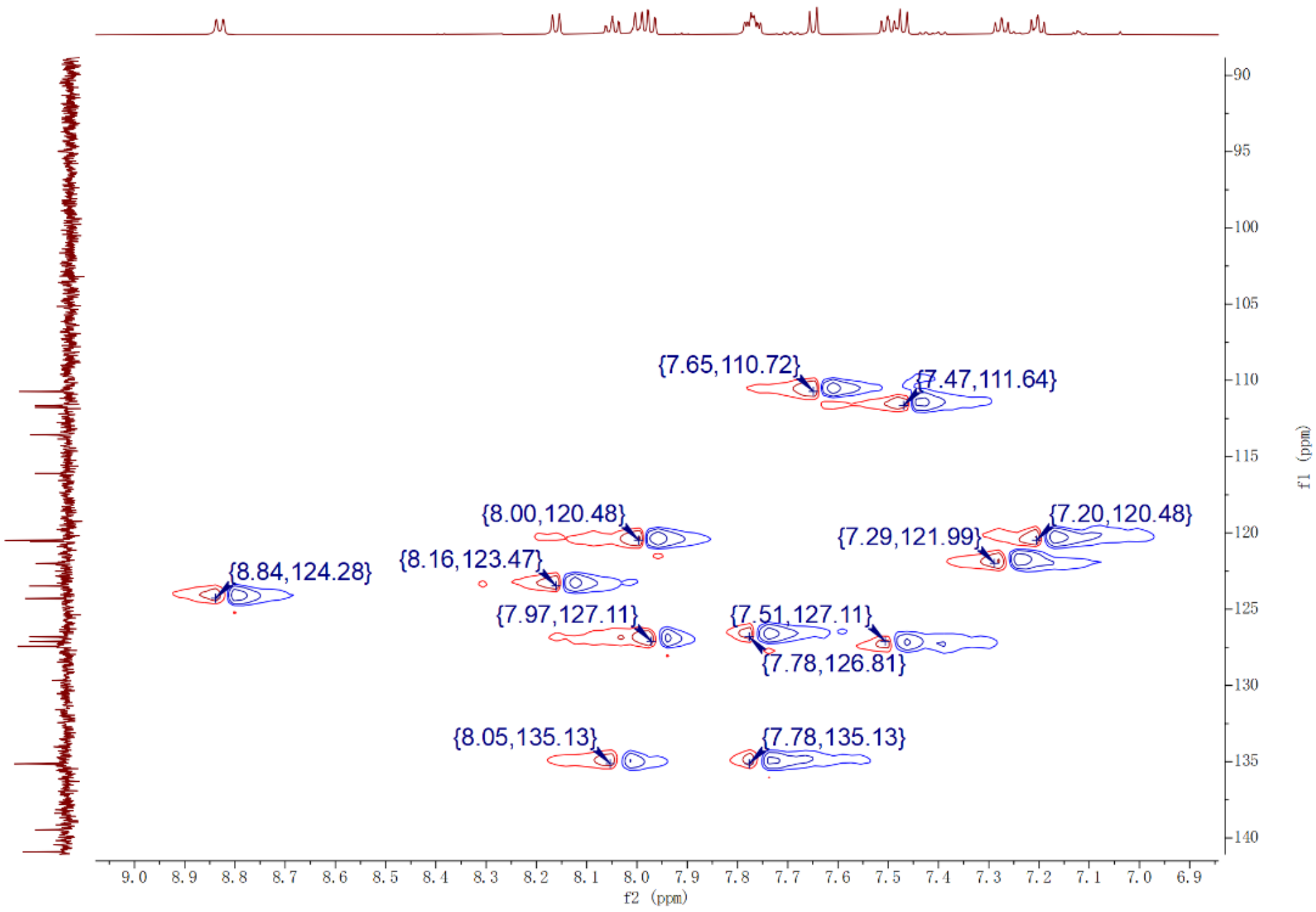

Figure S11 HSQC spectrum of 2-(3-amino-2*H*-indazol-2-yl)-*N*-(1*H*-indazol-3-yl)quinazolin-4-amine (DIQ01).

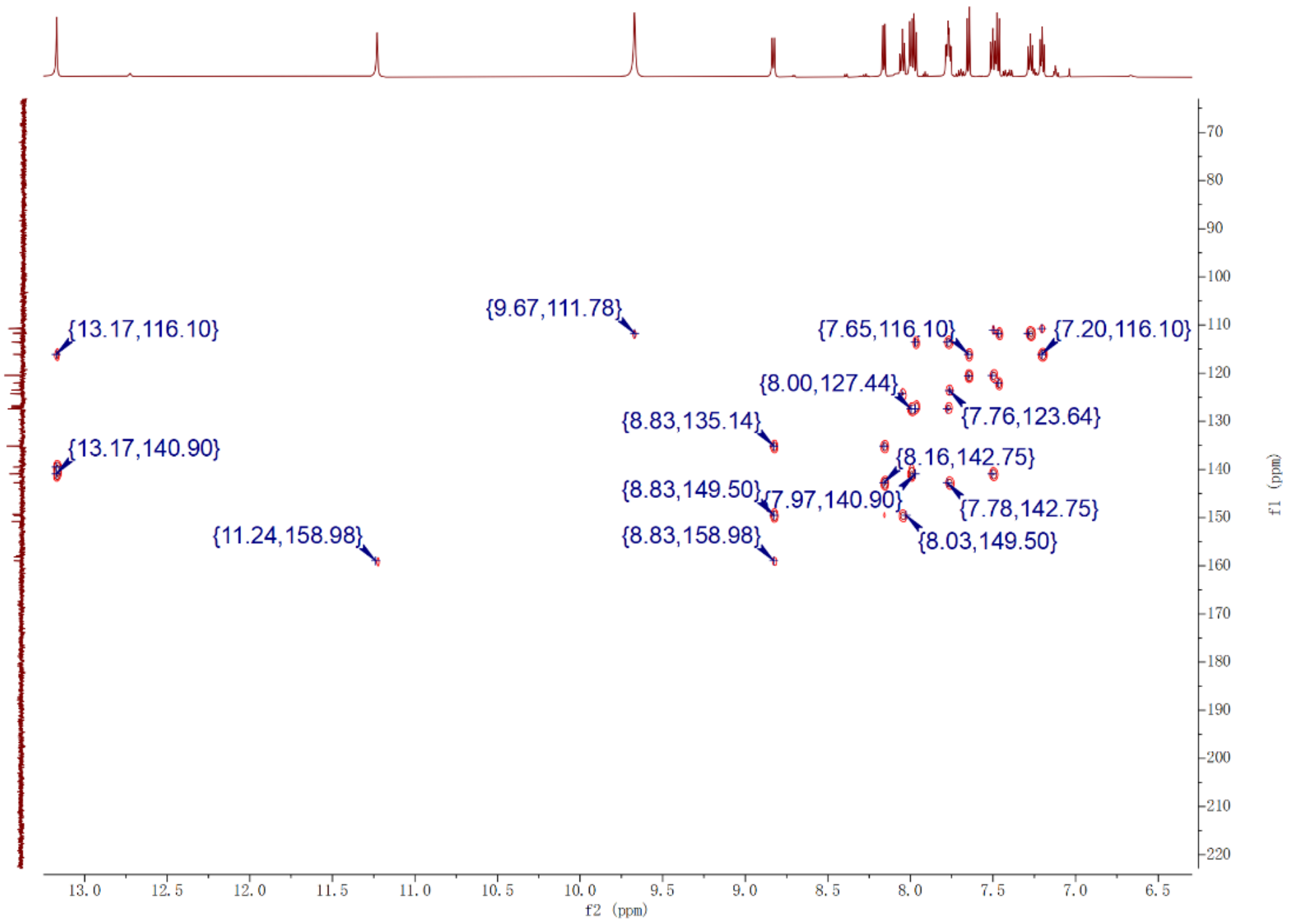

Figure S12 HMBC spectra of 2-(3-amino-*2H*-indazol-2-yl)-*N*-(*1H*-indazol-3-yl)quinazolin-4-amine (DIQ01).

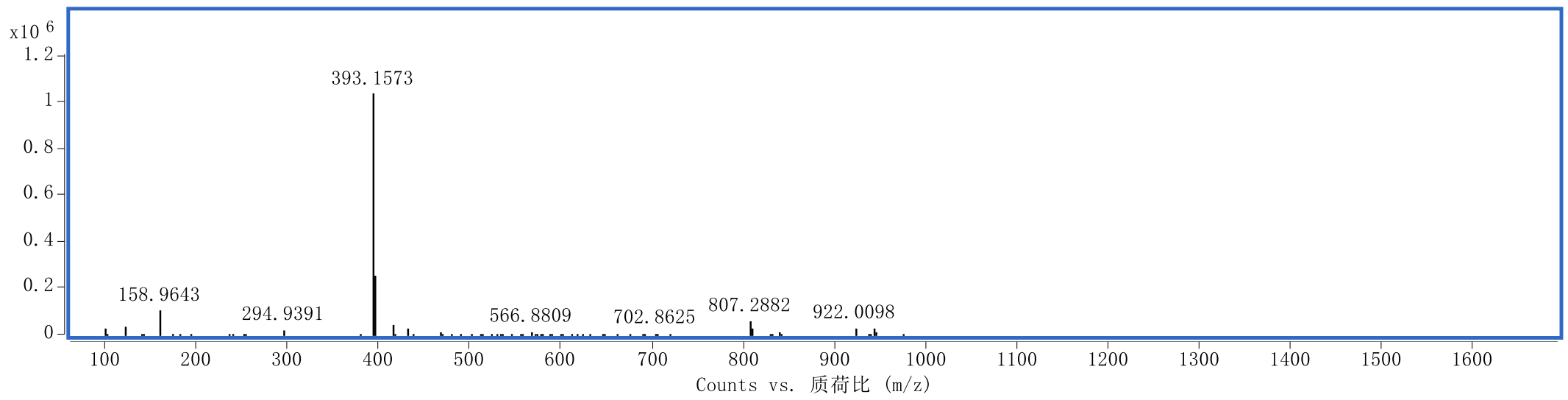

Figure S13 HRESIMS data of 2-(3-amino-*2H*-indazol-2-yl)-*N*-(*1H*-indazol-3-yl)quinazolin-4-amine (DIQ01).

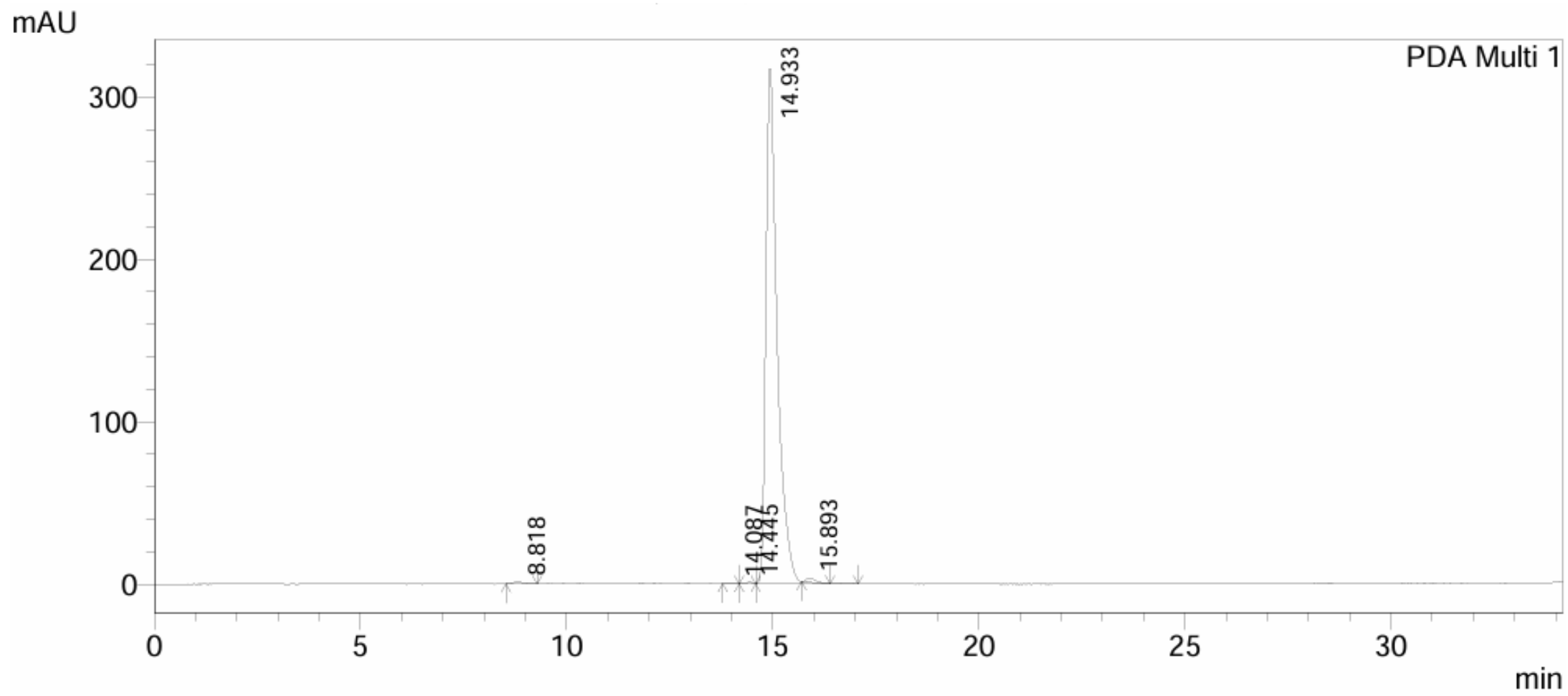

1 PDA Multi 1/254nm 4nm
PeakTable
PDA Ch1 254nm 4nm

Peak#	Ret. Time	Area	Height	Area %	Height %
1	8.818	21097	1098	0.343	0.340
2	14.087	5303	277	0.086	0.086
3	14.445	14227	894	0.231	0.277
4	14.933	6068708	317451	98.649	98.414
5	15.893	42466	2848	0.690	0.883
Total		6151801	322568	100.000	100.000

Figure S14 HPLC of DIQ01, 30%–70% 30 min, MeCN (0.05% TFA)-H_2O (0.05% TFA).

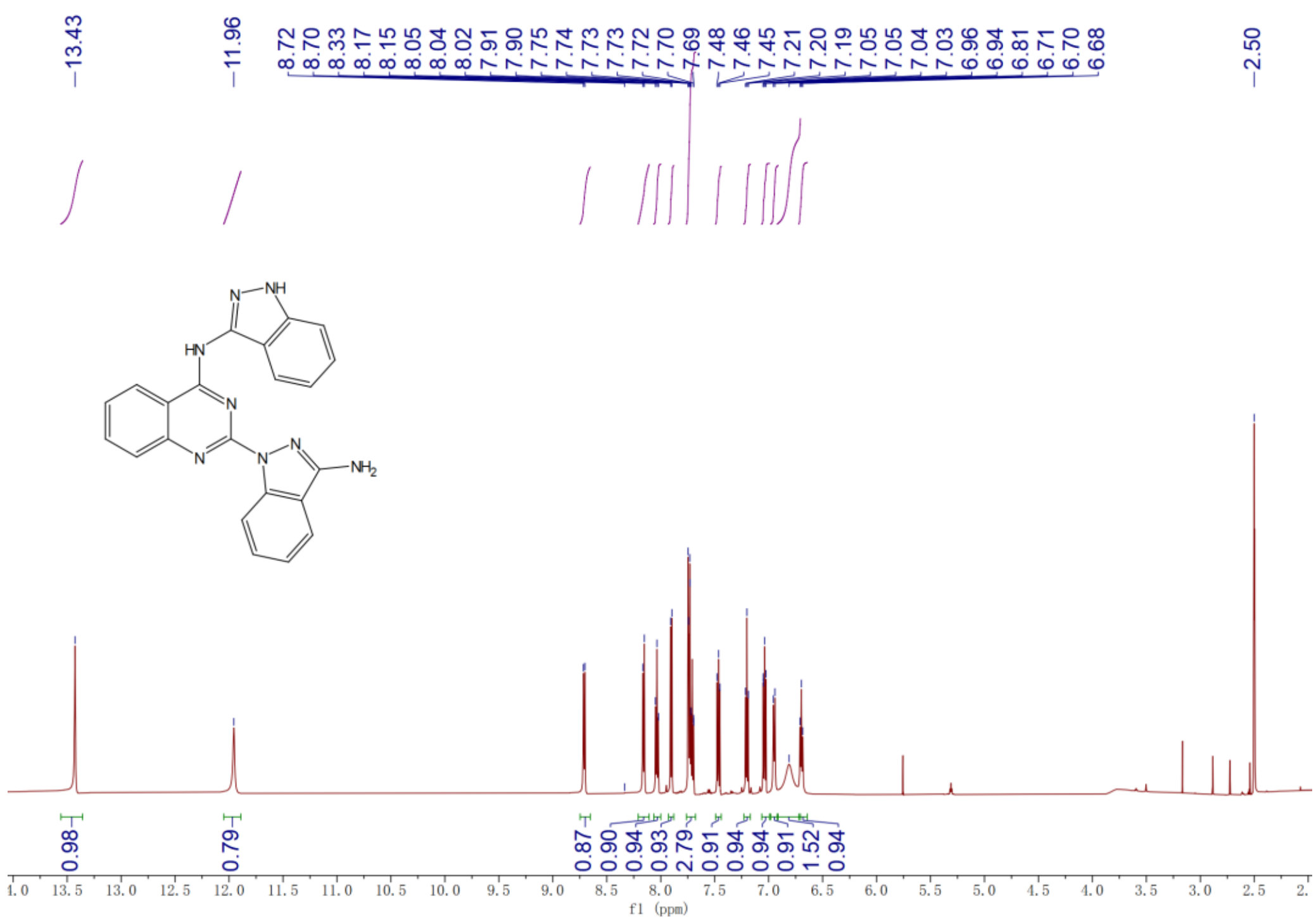

Figure S15 ^{1}H NMR spectrum of 2-(3-amino-*1H*-indazol-1-yl)-*N*-(*1H*-indazol-3-yl)quinazolin-4-amine (DIQ02).

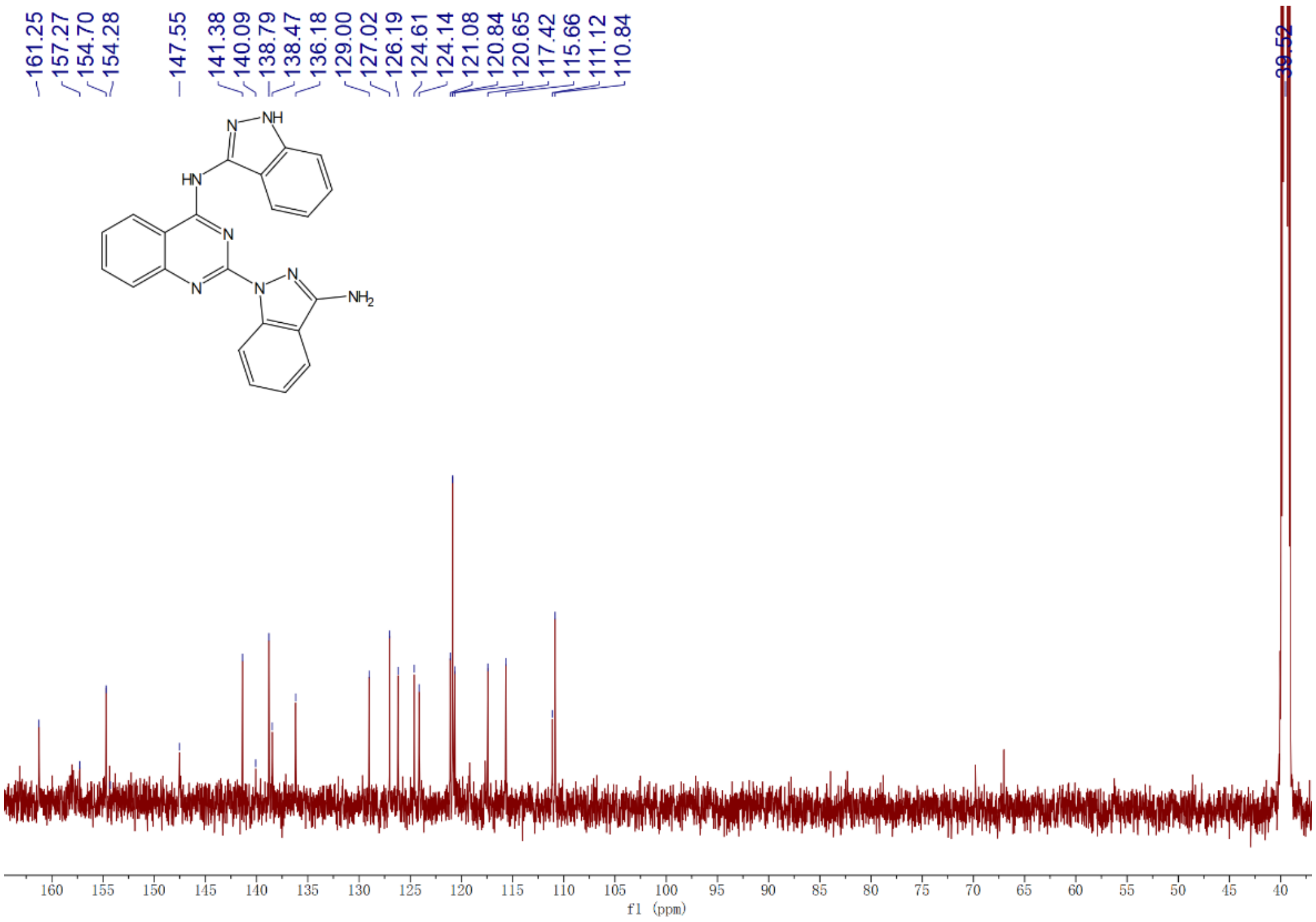

Figure S16 ^{13}C NMR spectra of 2-(3-amino-1*H*-indazol-1-yl)-*N*-(1*H*-indazol-3-yl)quinazolin-4-amine (DIQ02).

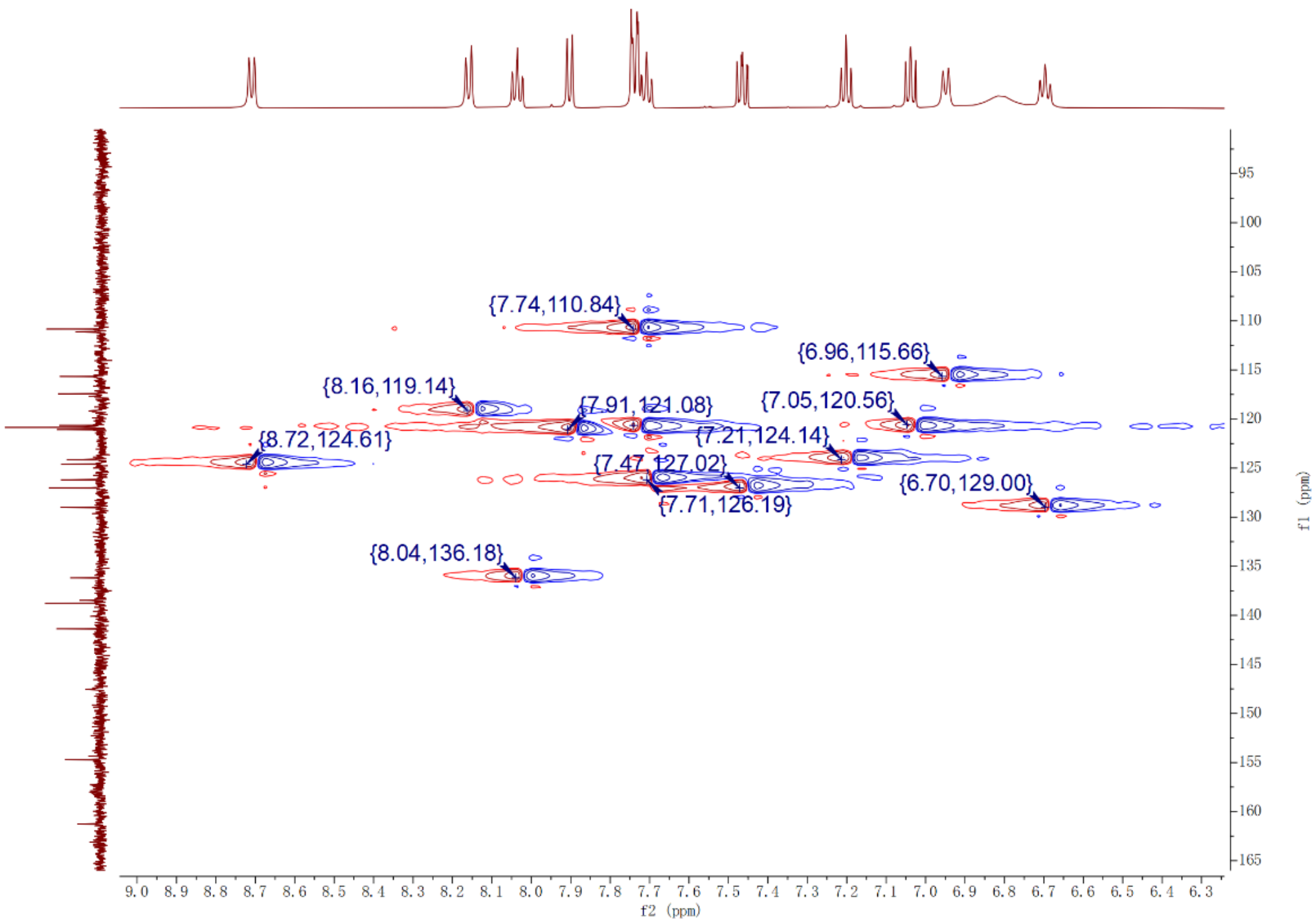

Figure S17 HSQC spectrum of 2-(3-amino-1*H*-indazol-1-yl)-*N*-(1*H*-indazol-3-yl)quinazolin-4-amine (DIQ02).

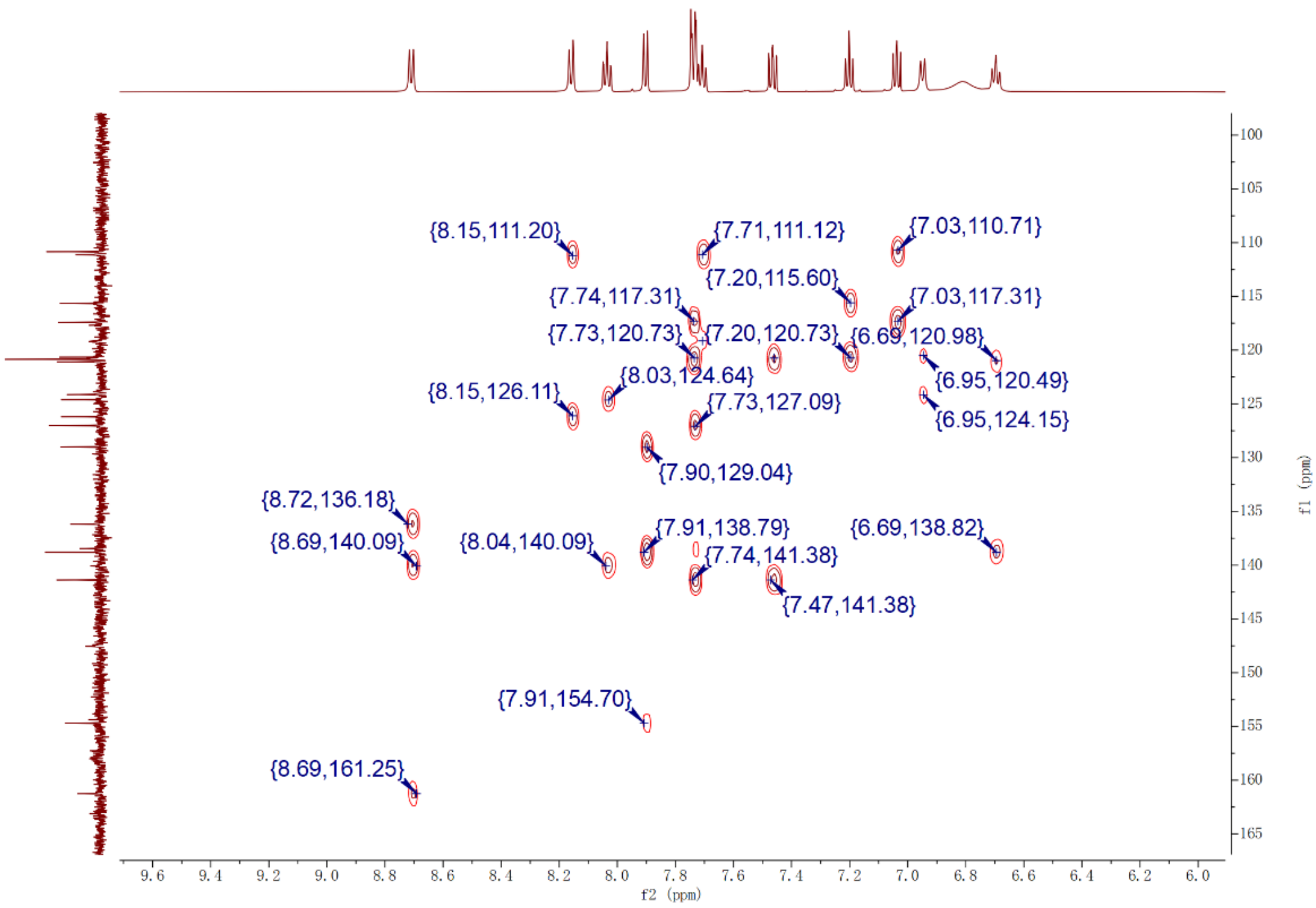

Figure S18 HMBC spectra of 2-(3-amino-1*H*-indazol-1-yl)-*N*-(1*H*-indazol-3-yl)quinazolin-4-amine (DIQ02).

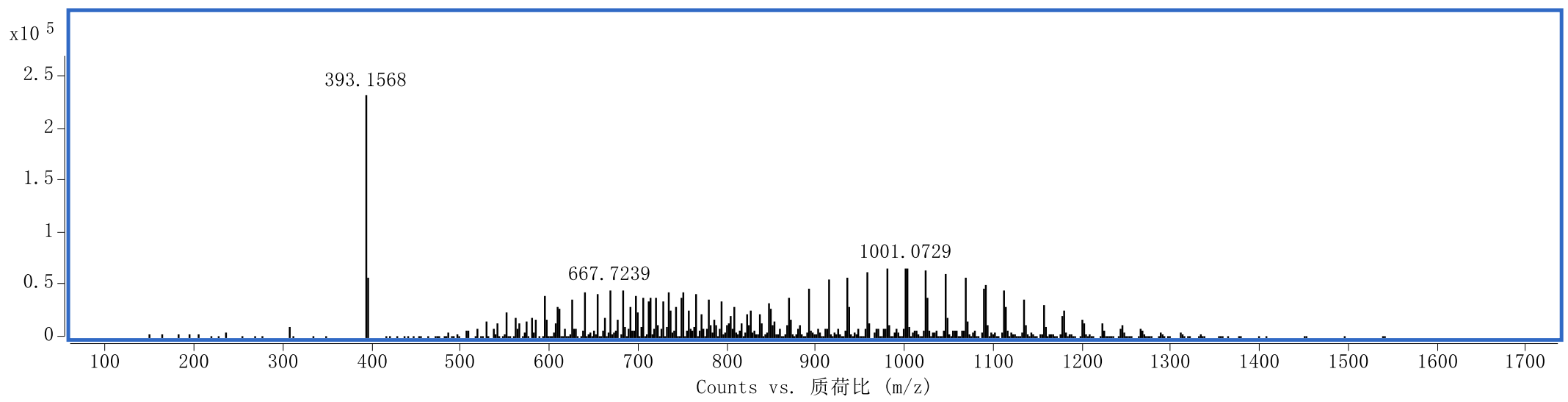

Figure S19 HRESIMS data of 2-(3-amino-1*H*-indazol-1-yl)-*N*-(1*H*-indazol-3-yl)quinazolin-4-amine (DIQ02).

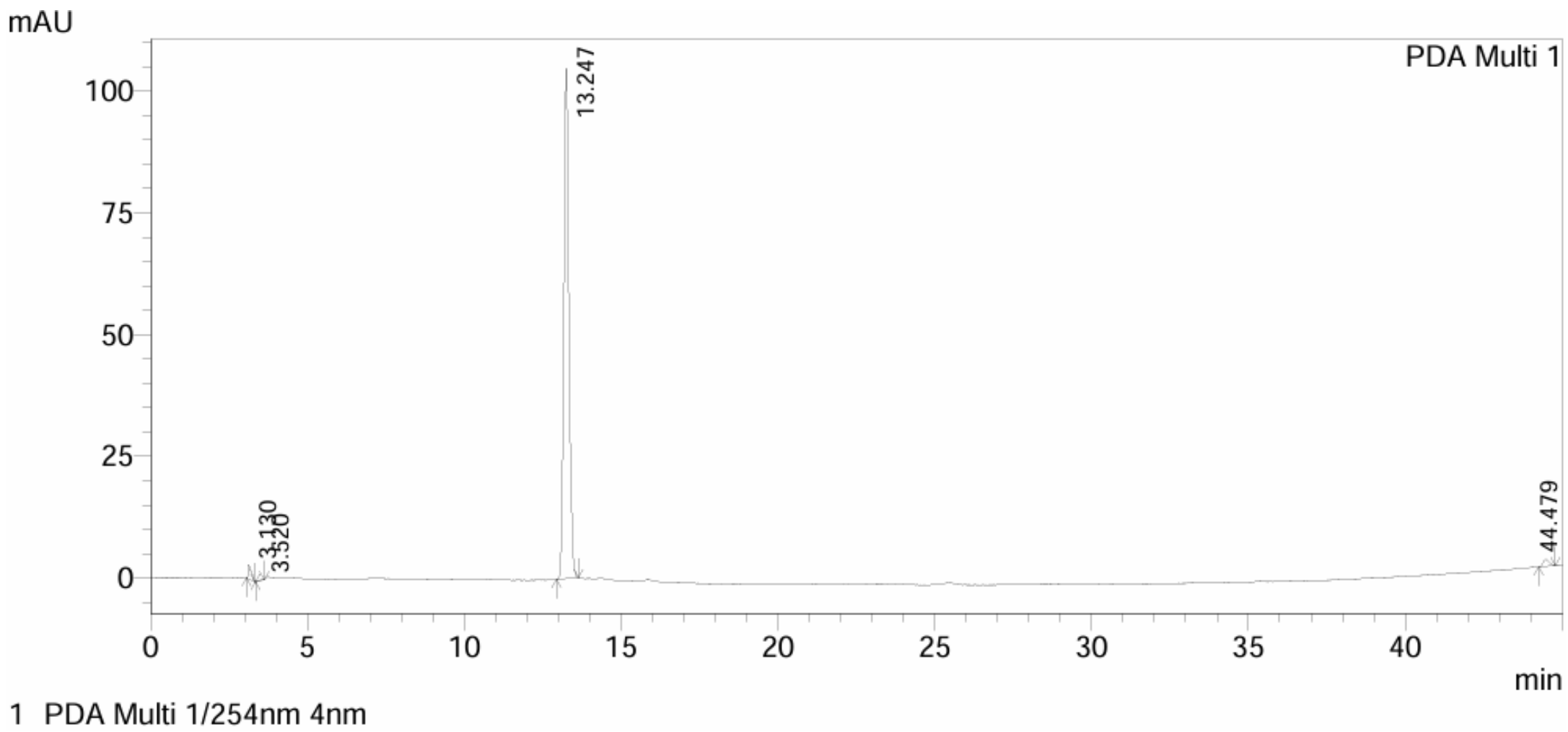

PeakTable

PDA Ch1 254nm 4nm

Peak#	Ret. Time	Area	Height	Area %	Height %
1	3.130	18459	2936	1.528	2.685
2	3.520	2160	192	0.179	0.175
3	13.247	1169123	104851	96.753	95.876
4	44.479	18619	1382	1.541	1.263
Total		1208361	109361	100.000	100.000

Figure S20 HPLC of DIQ02**,** 30%–70% 30 min, MeCN (0.05% TFA)-H_2O (0.05% TFA).

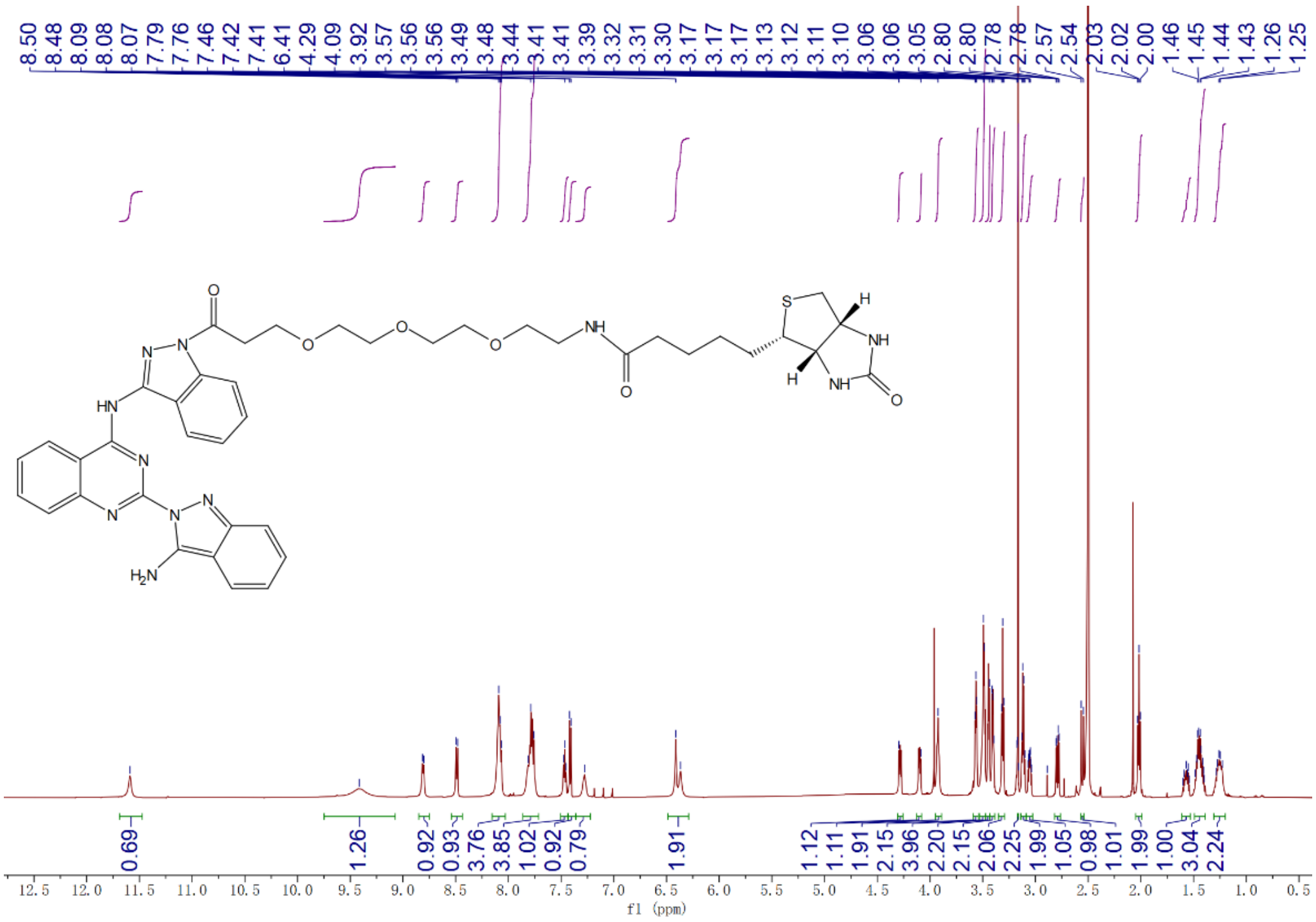

Figure S21 ^{1}H NMR spectrum of Biotin-DIQ01.

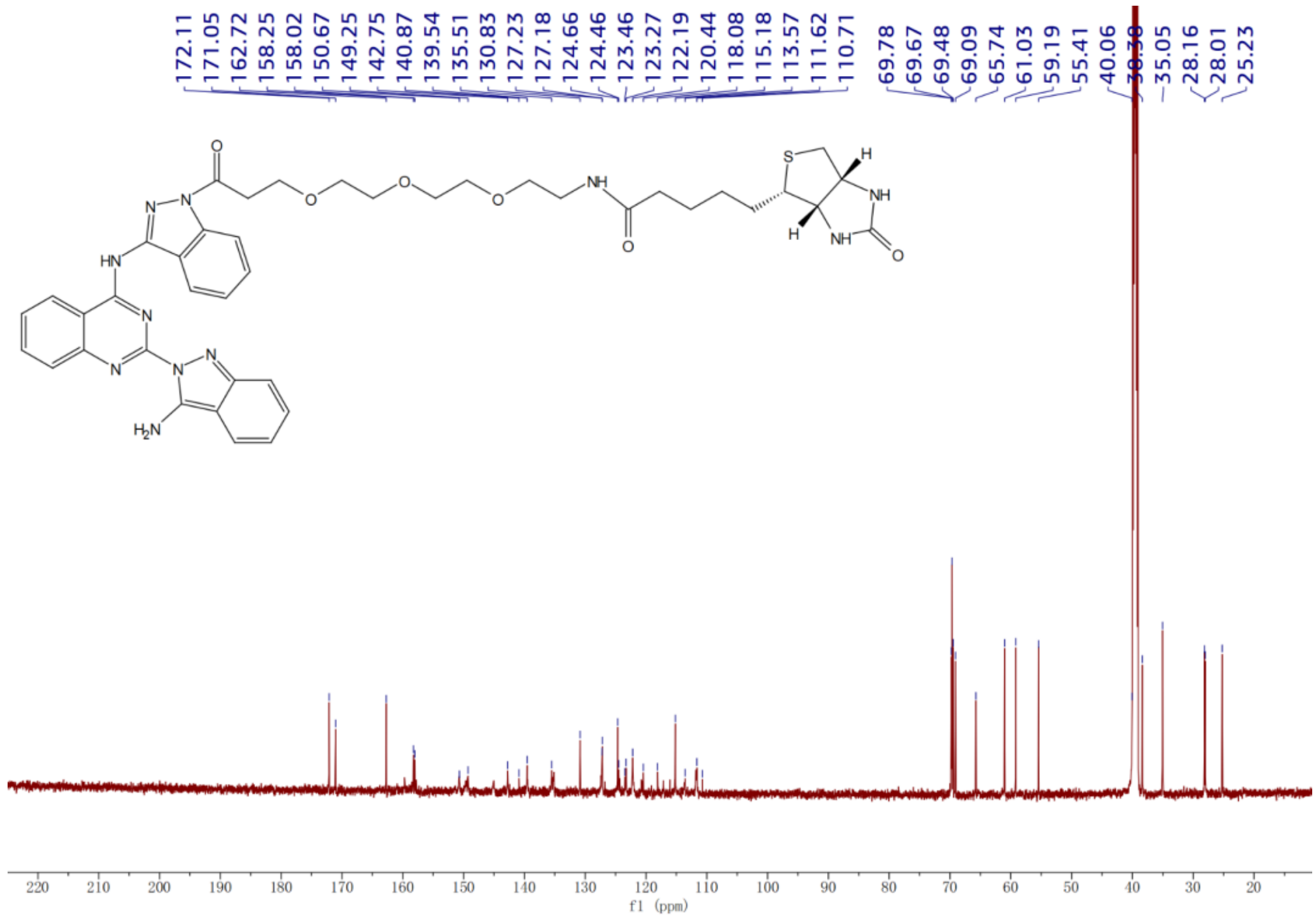

Figure S22 ^{13}C NMR spectra of Biotin-DIQ01.

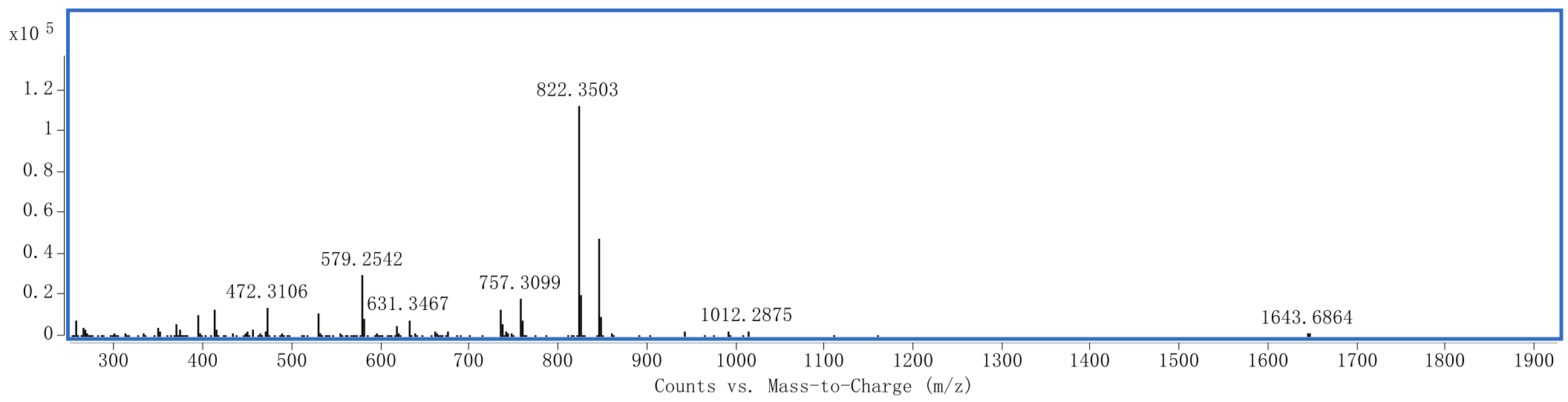

Figure S23 HRESIMS data of Biotin-DIQ01.

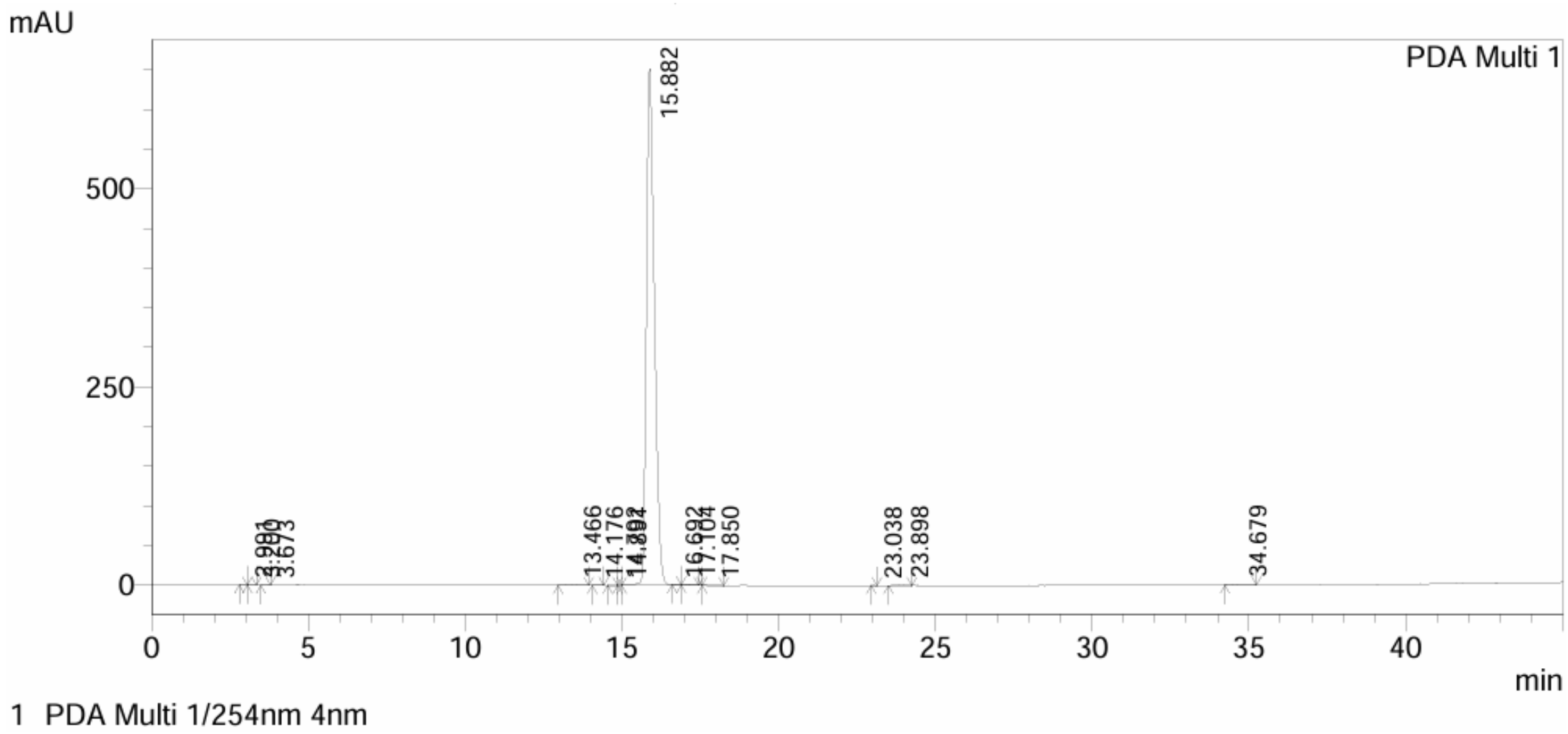

PeakTable

PDA Ch1 254nm 4nm

Peak#	Ret. Time	Area	Height	Area %	Height %
1	2.991	2023	176	0.017	0.027
2	3.200	4085	385	0.035	0.058
3	3.673	2497	204	0.021	0.031
4	13.466	28890	1534	0.248	0.233
5	14.176	1620	90	0.014	0.014
6	14.792	2496	214	0.021	0.032
7	14.891	1097	184	0.009	0.028
8	15.882	11512295	652171	98.957	99.000
9	16.692	1547	151	0.013	0.023
10	17.104	16431	1078	0.141	0.164
11	17.850	6595	336	0.057	0.051
12	23.038	1471	166	0.013	0.025
13	23.898	19311	887	0.166	0.135
14	34.679	33267	1181	0.286	0.179
Total		11633625	658756	100.000	100.000

Figure S24 HPLC of Biotin-DIQ01, 30%–70% 30 min, MeCN (0.05% TFA)-H_2O (0.05% TFA).

3. Western blot grayscale quantification and statistical analysis

Figure 1G

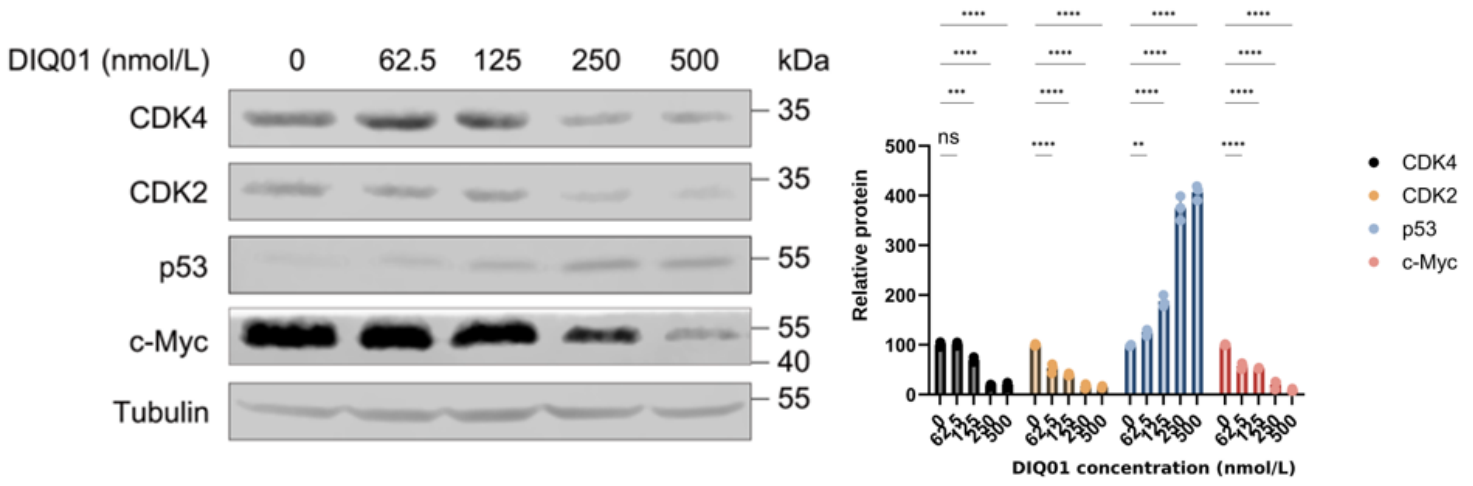

Figure1H

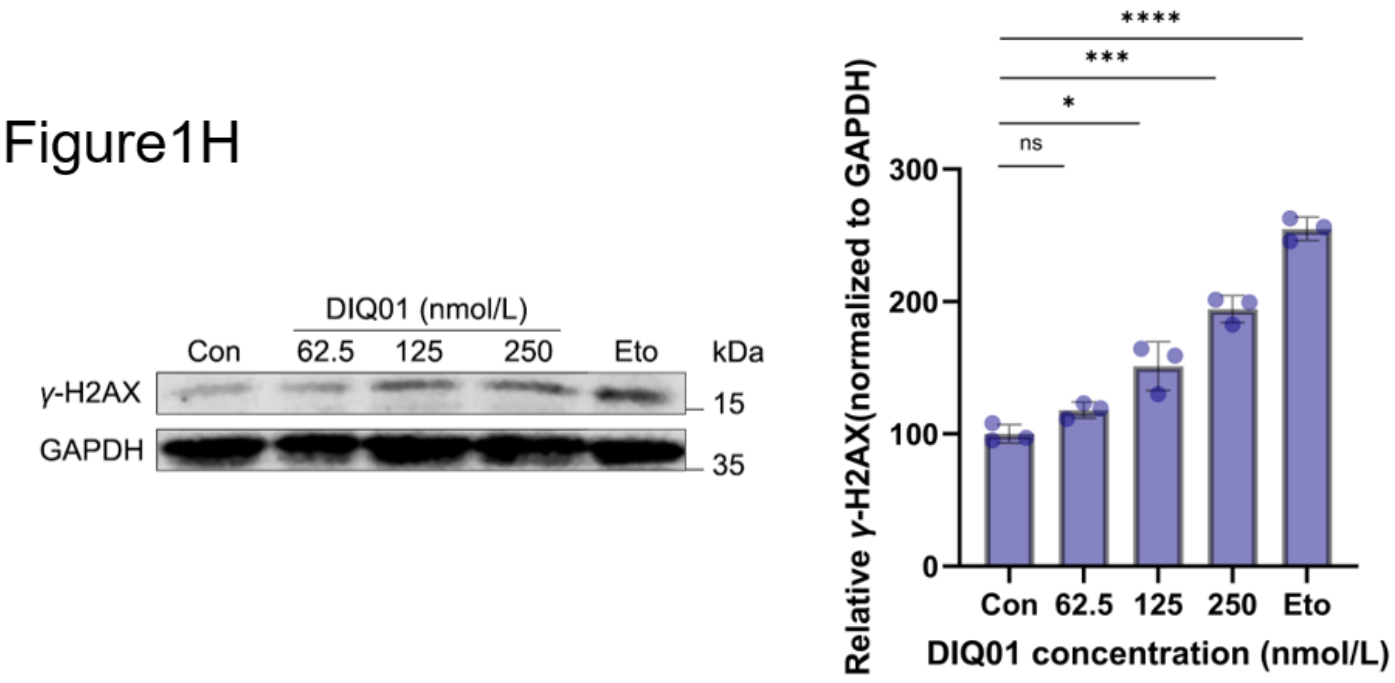

Figure 4C

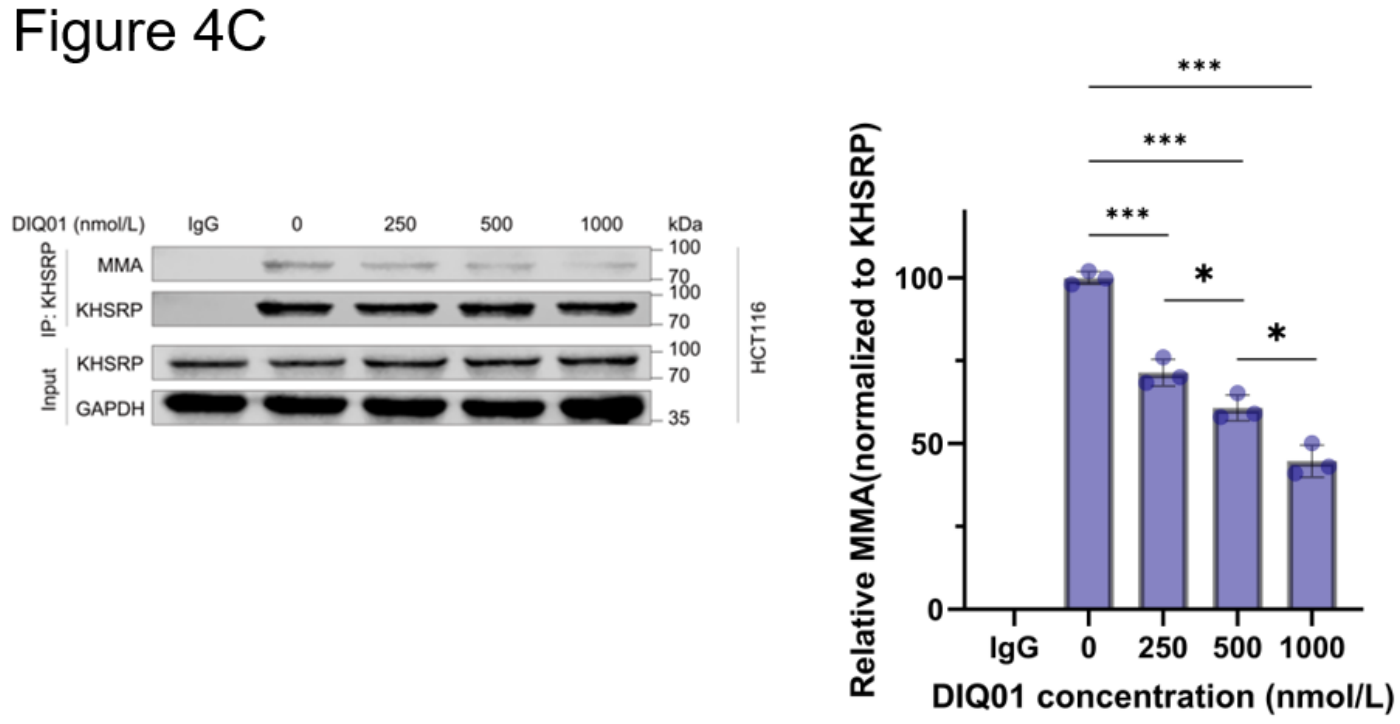

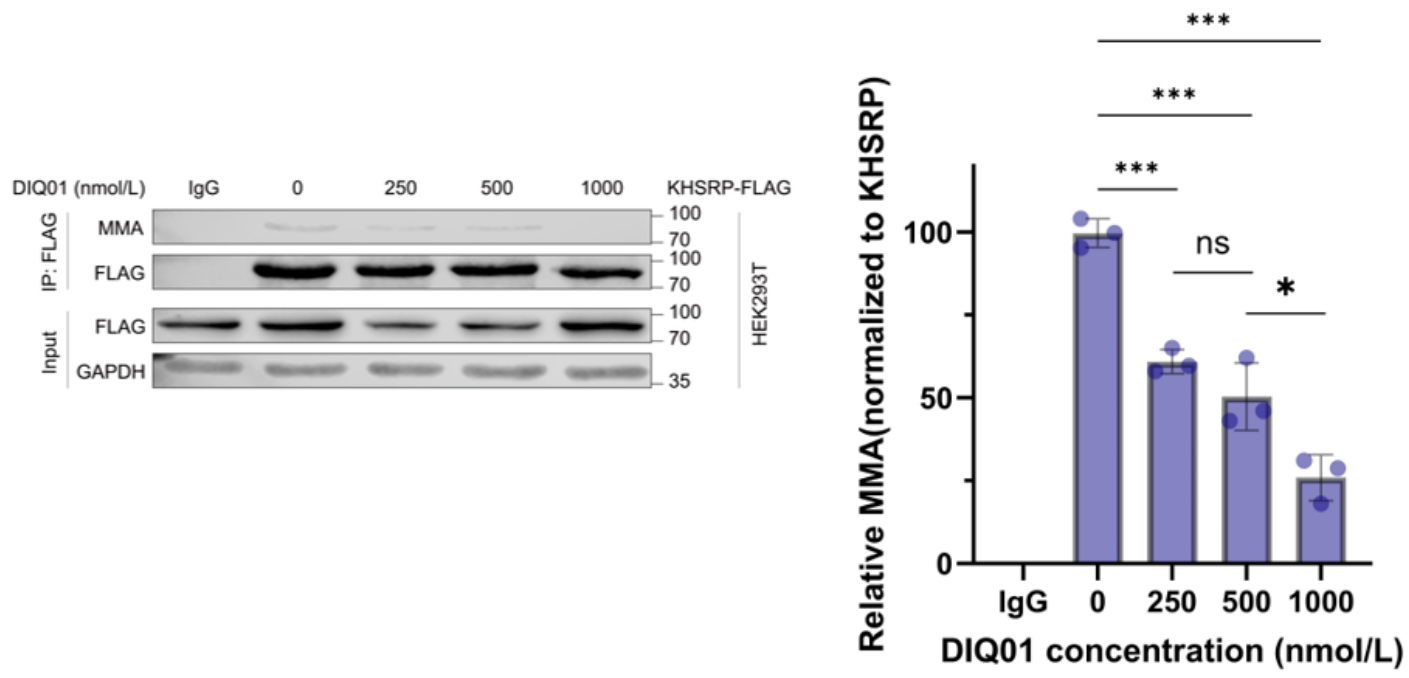

Figure 4E

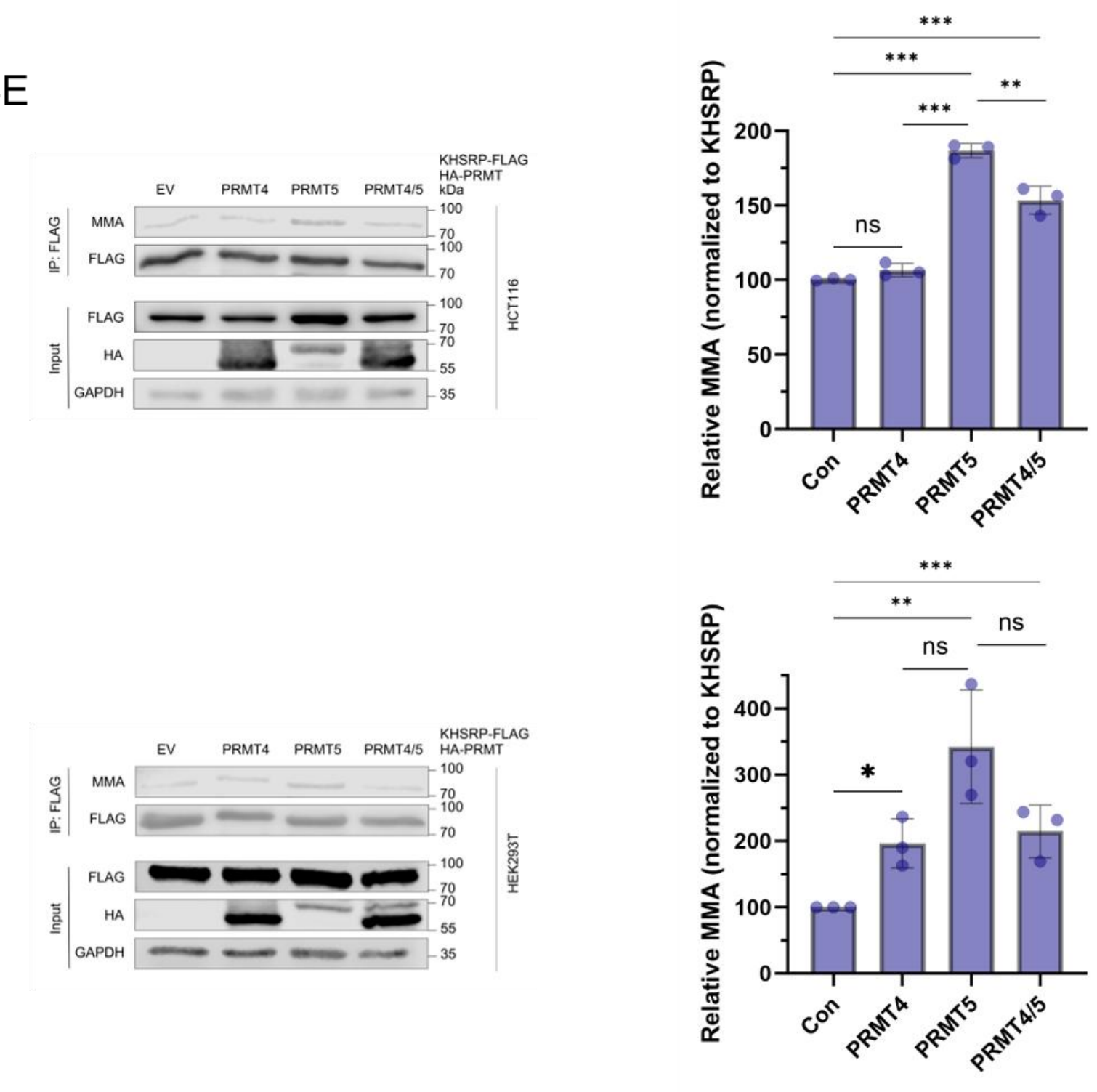

Figure 4F

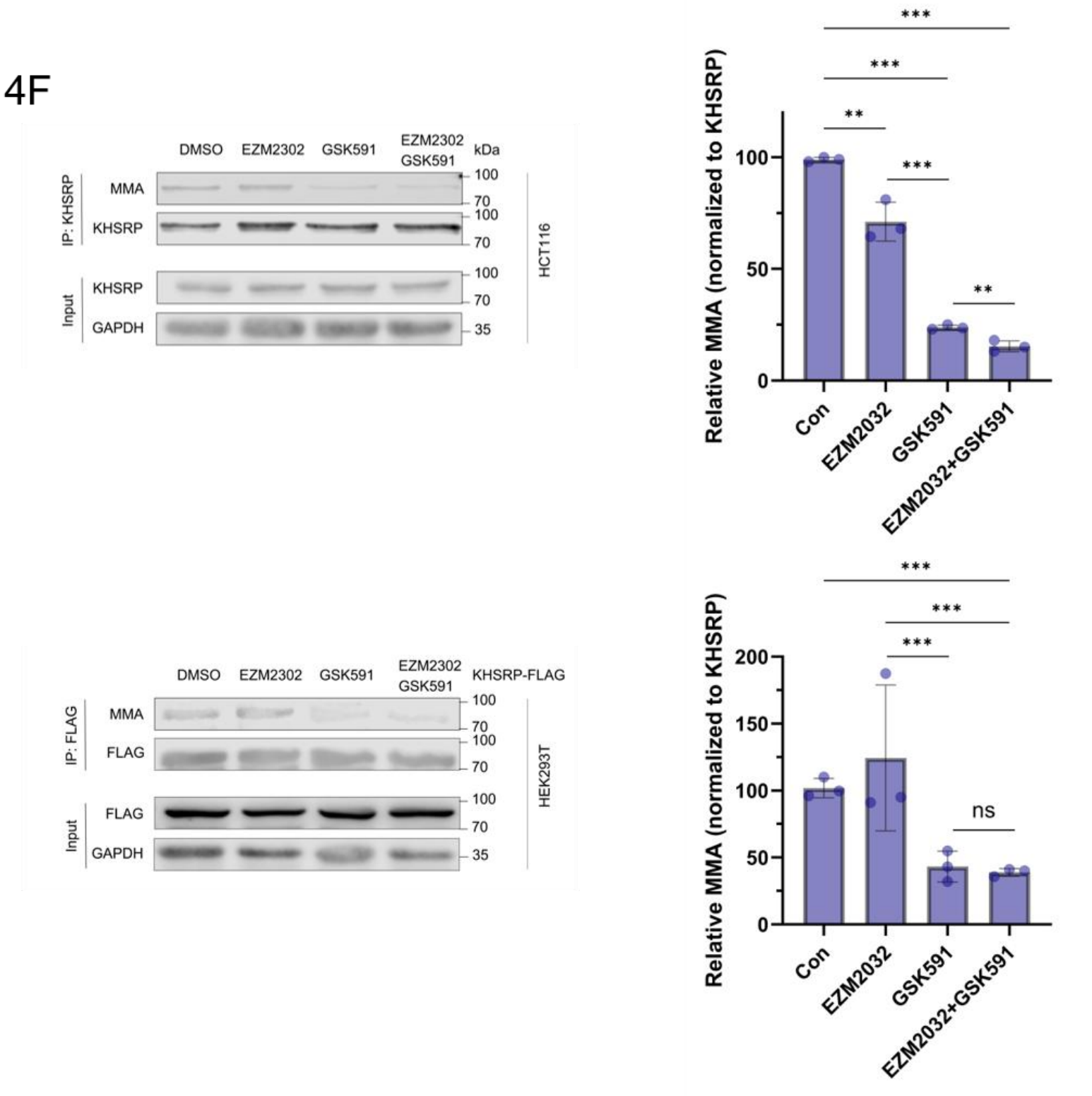

Figure 4G

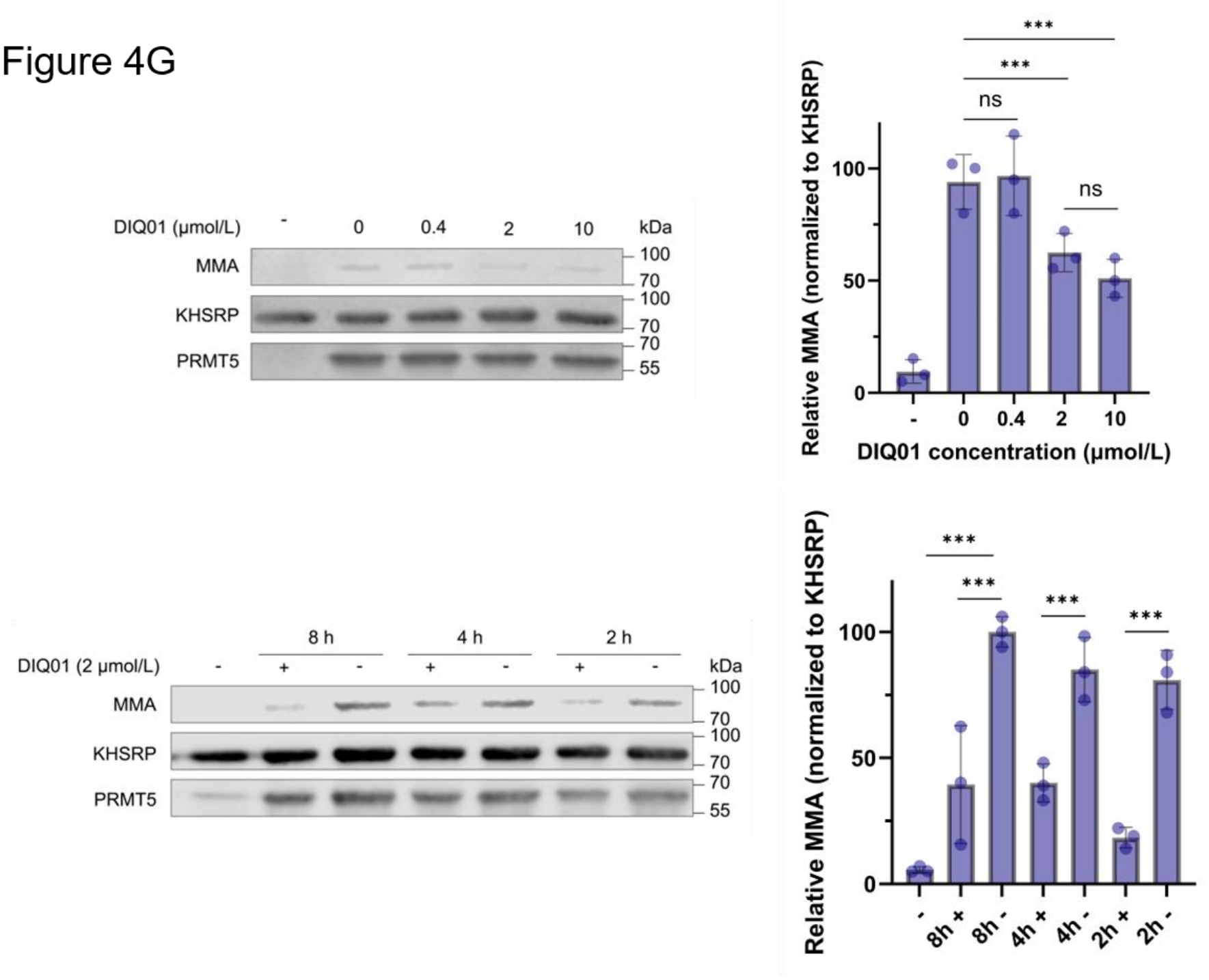

Figure 4L

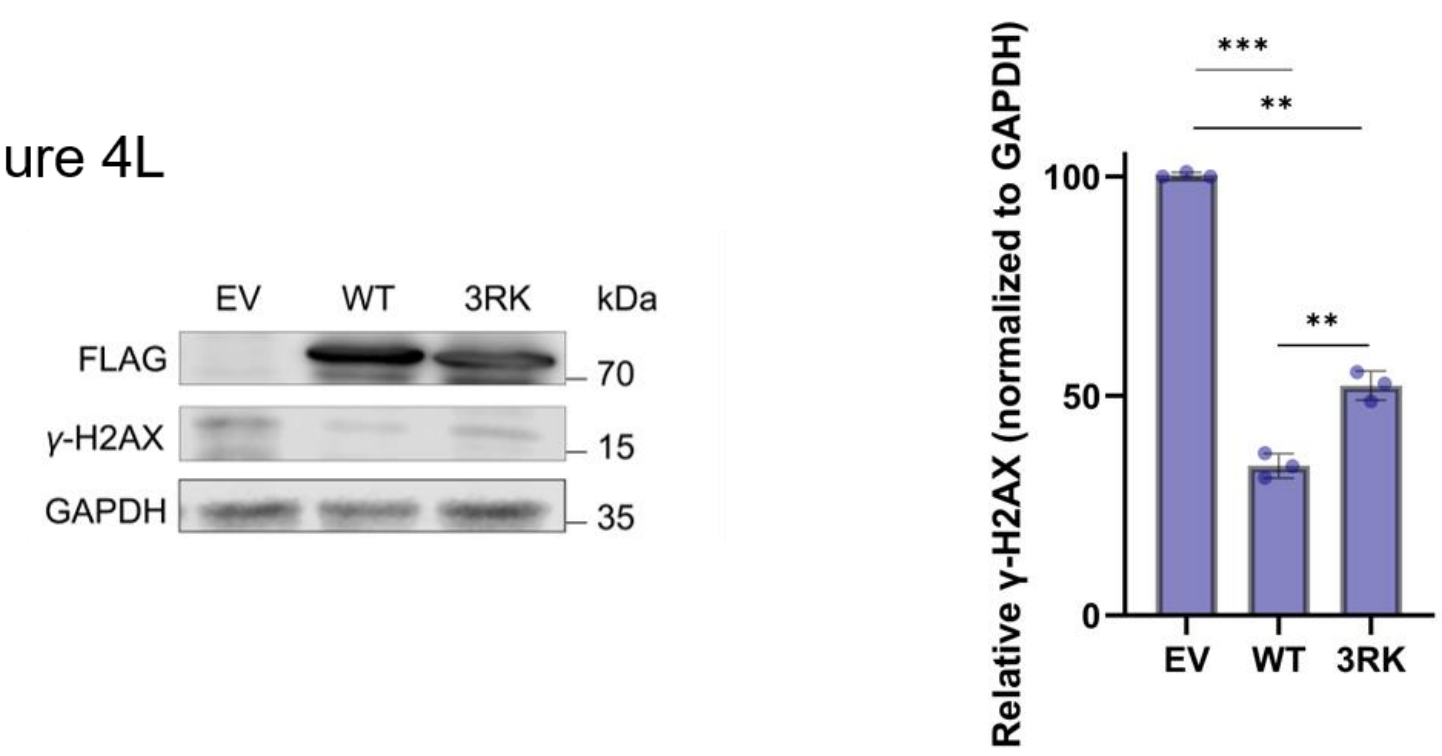

Figure S4C

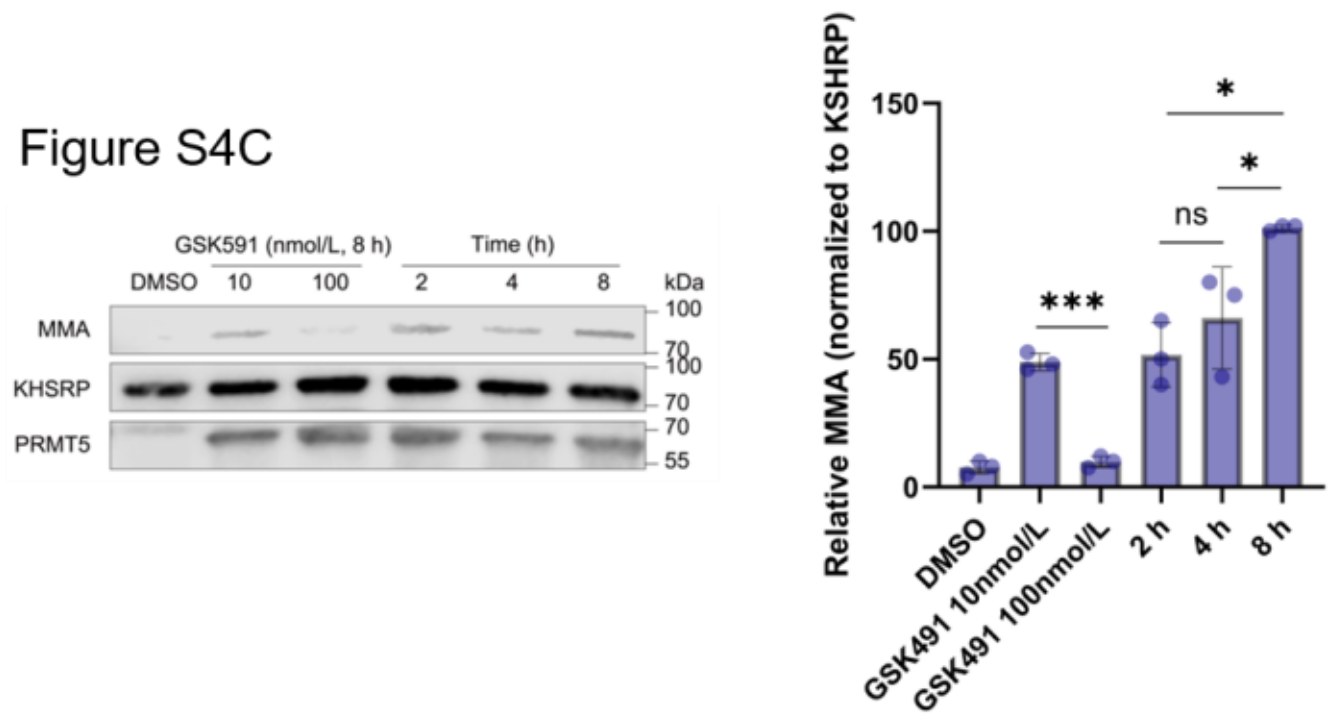

Figure 6H

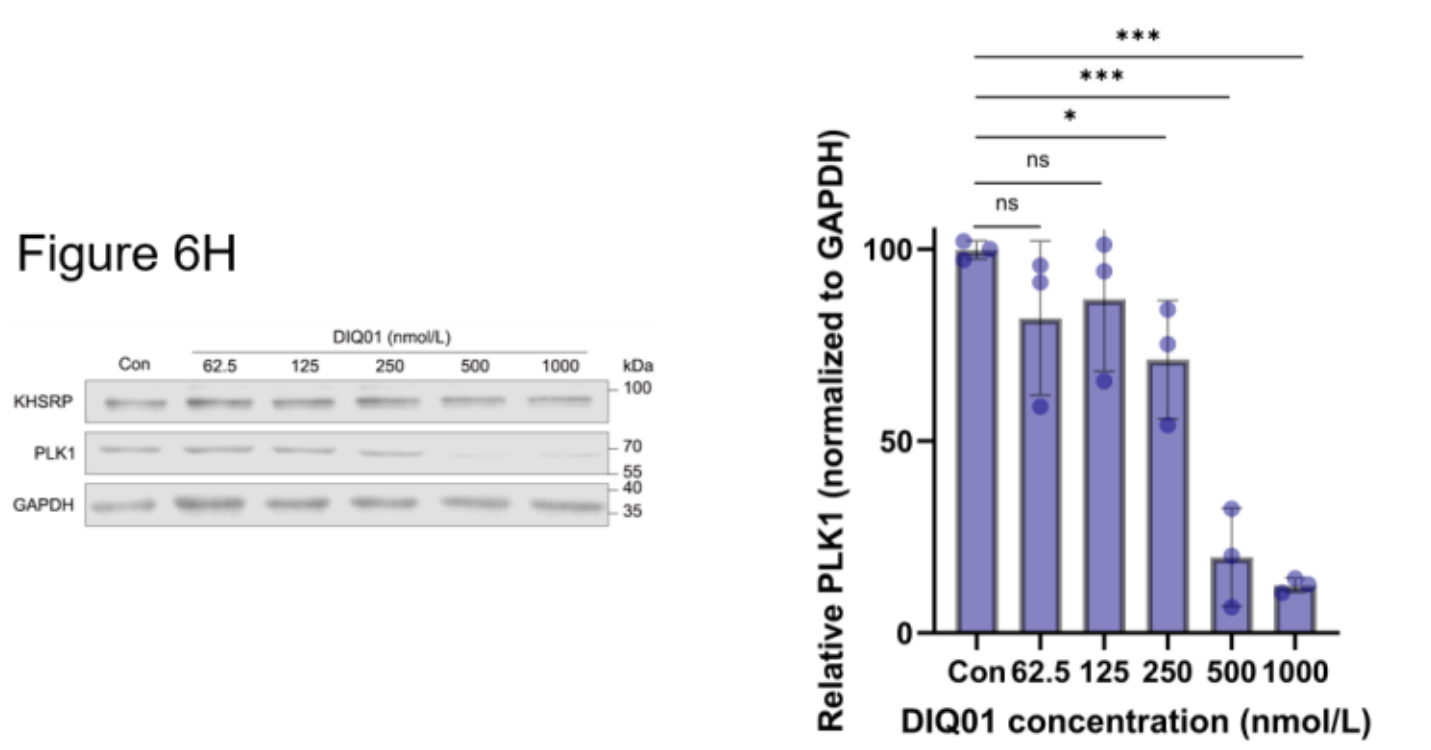

Figure 6T

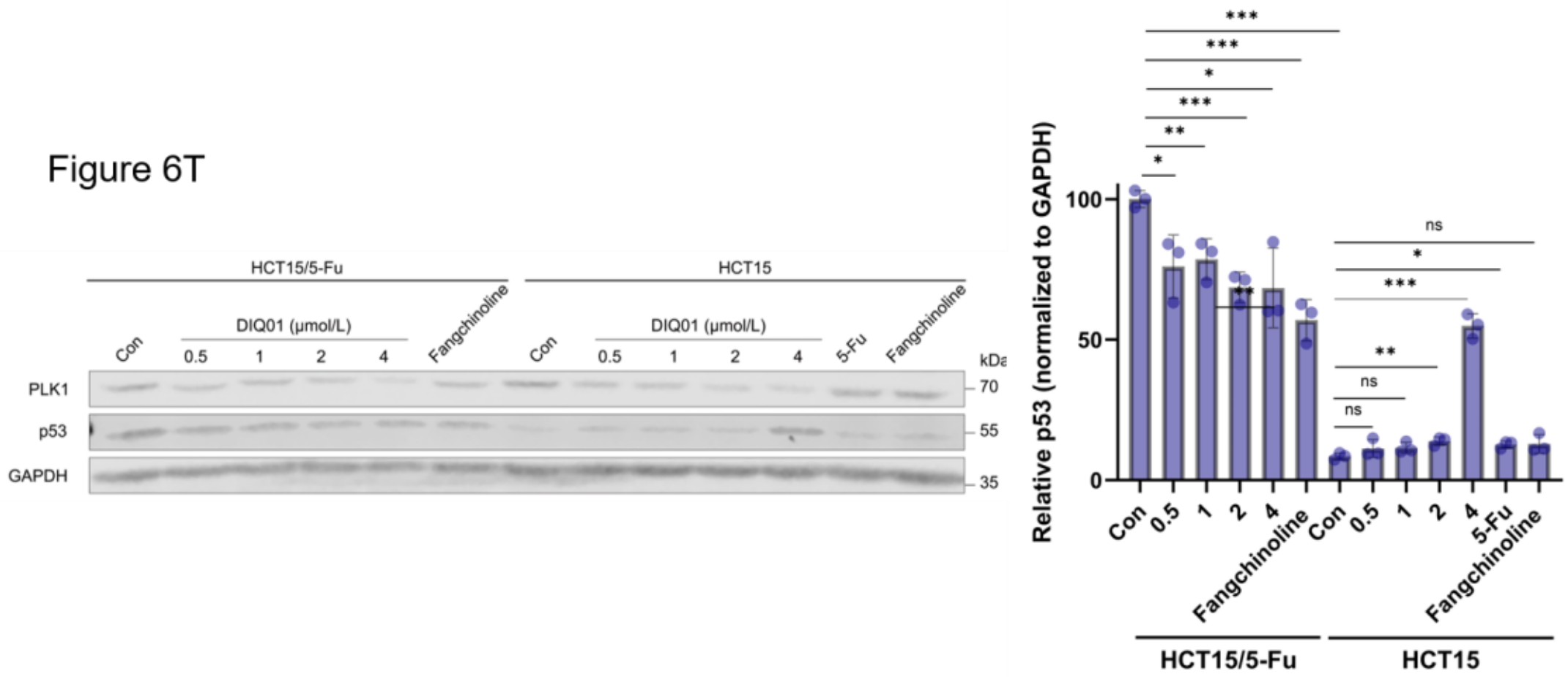

4. Uncropped blots

Figure 1G

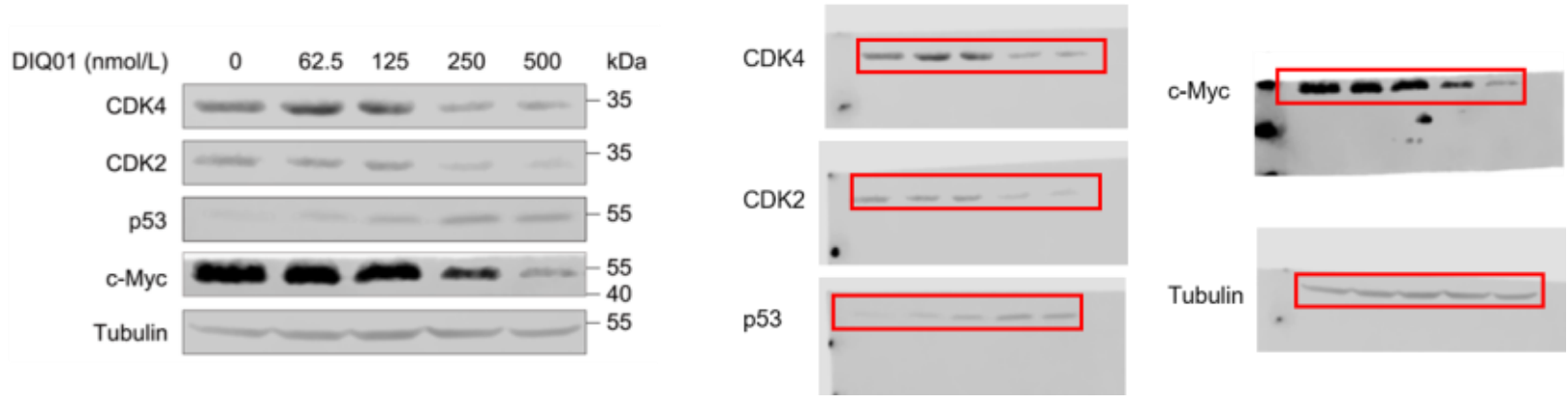

Figure 1H

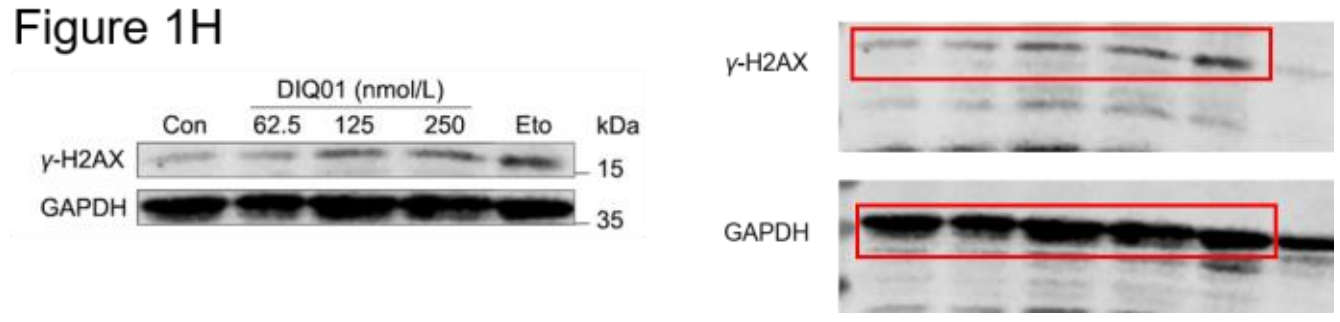

Figure2G

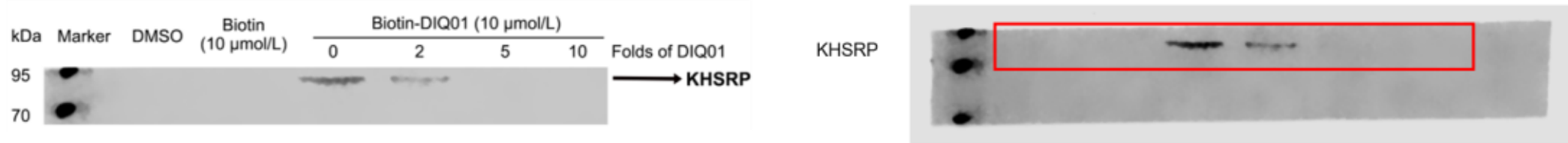

Figure 2H

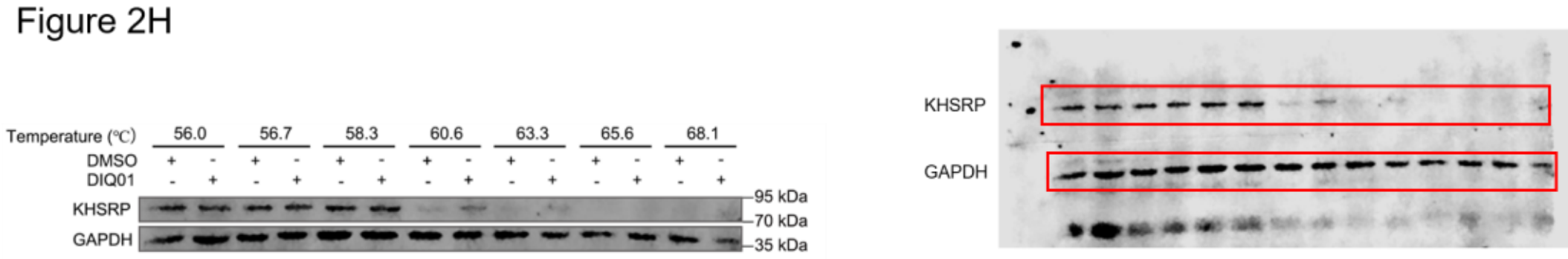

Figure S2D

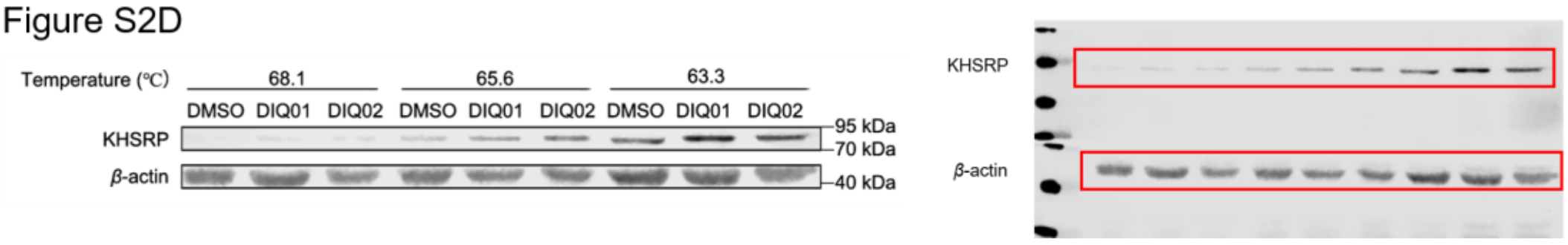

Figure 3E

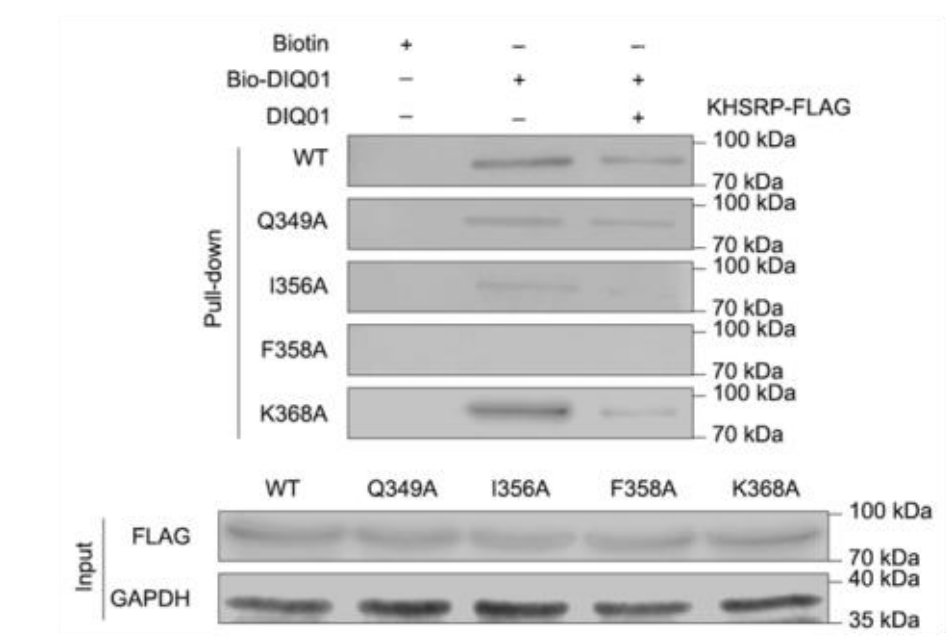

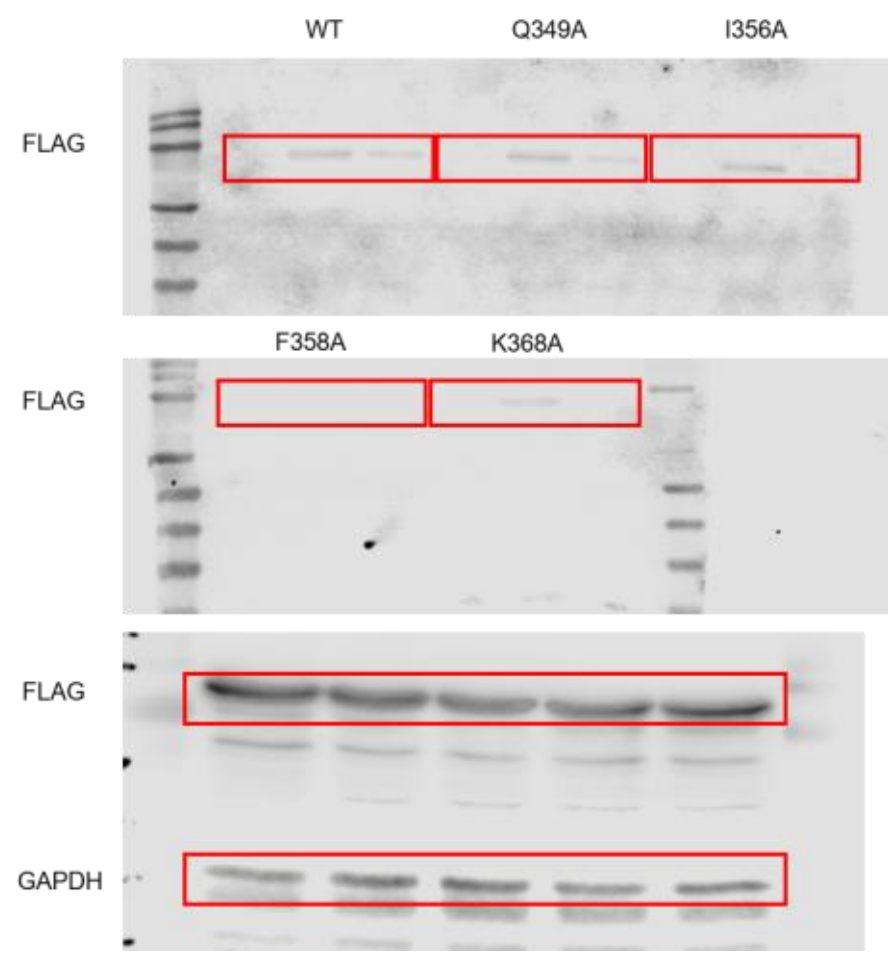

Figure 4C

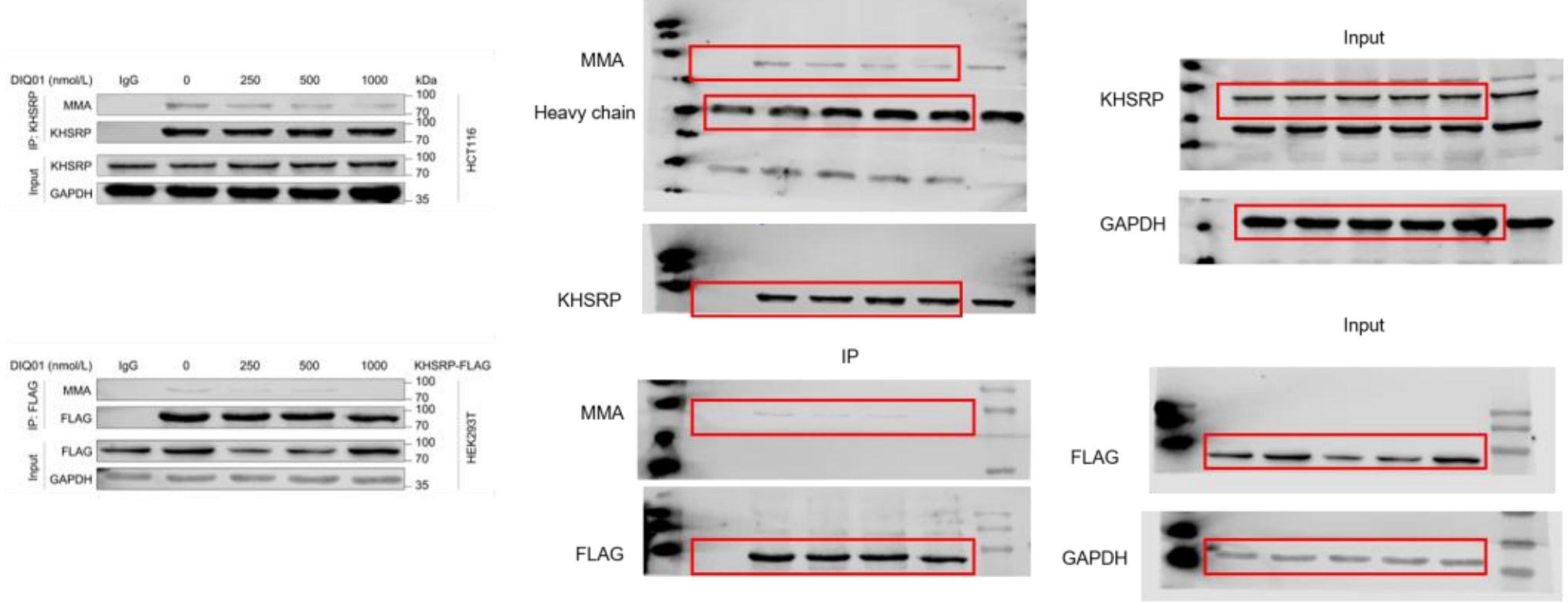

Figure 4E

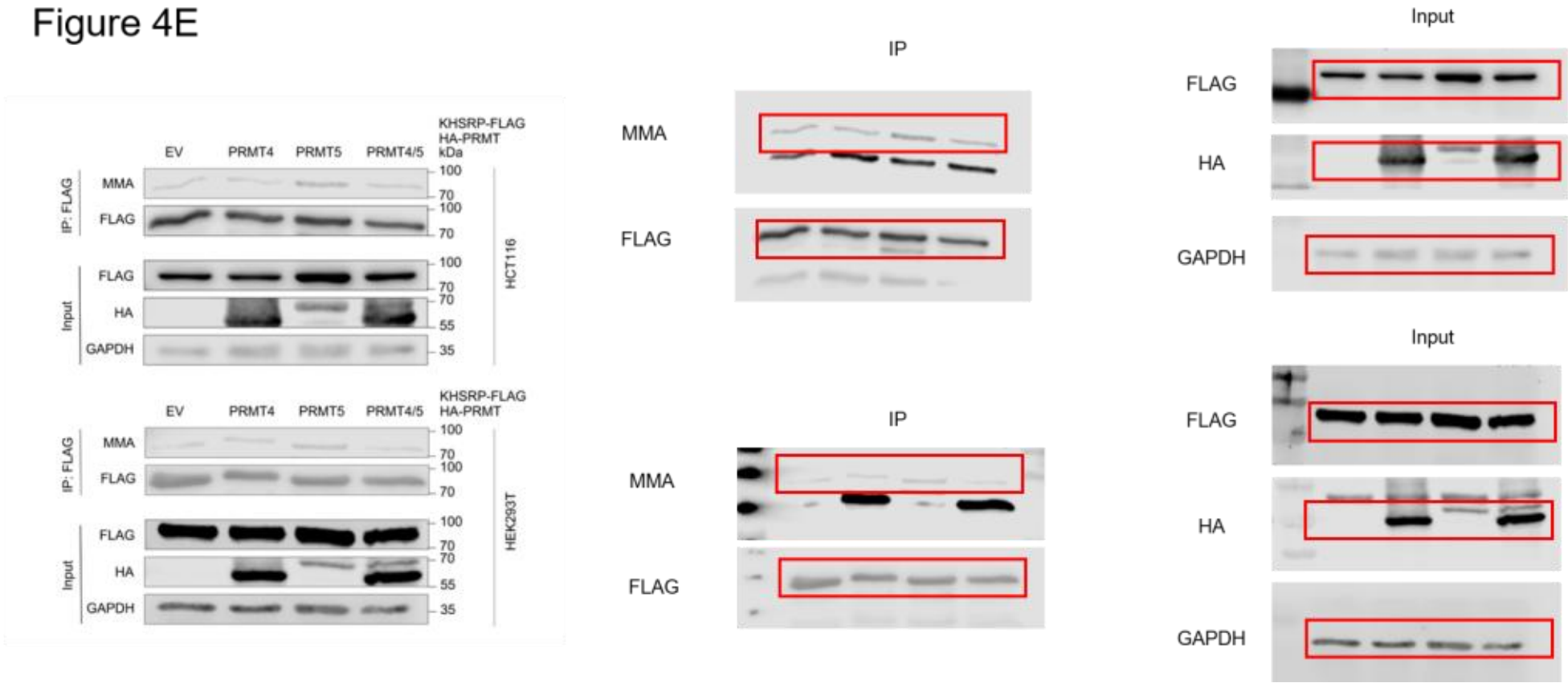

Figure 4F

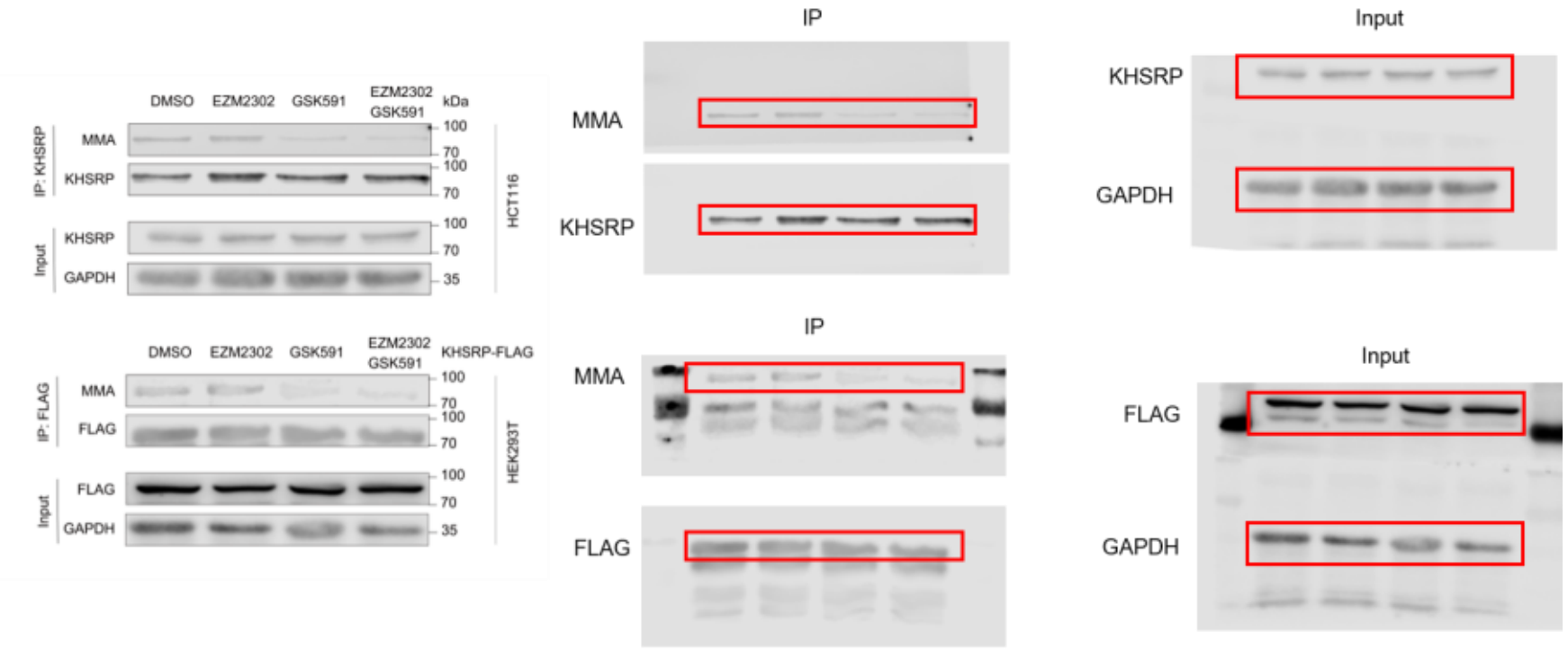

Figure 4G

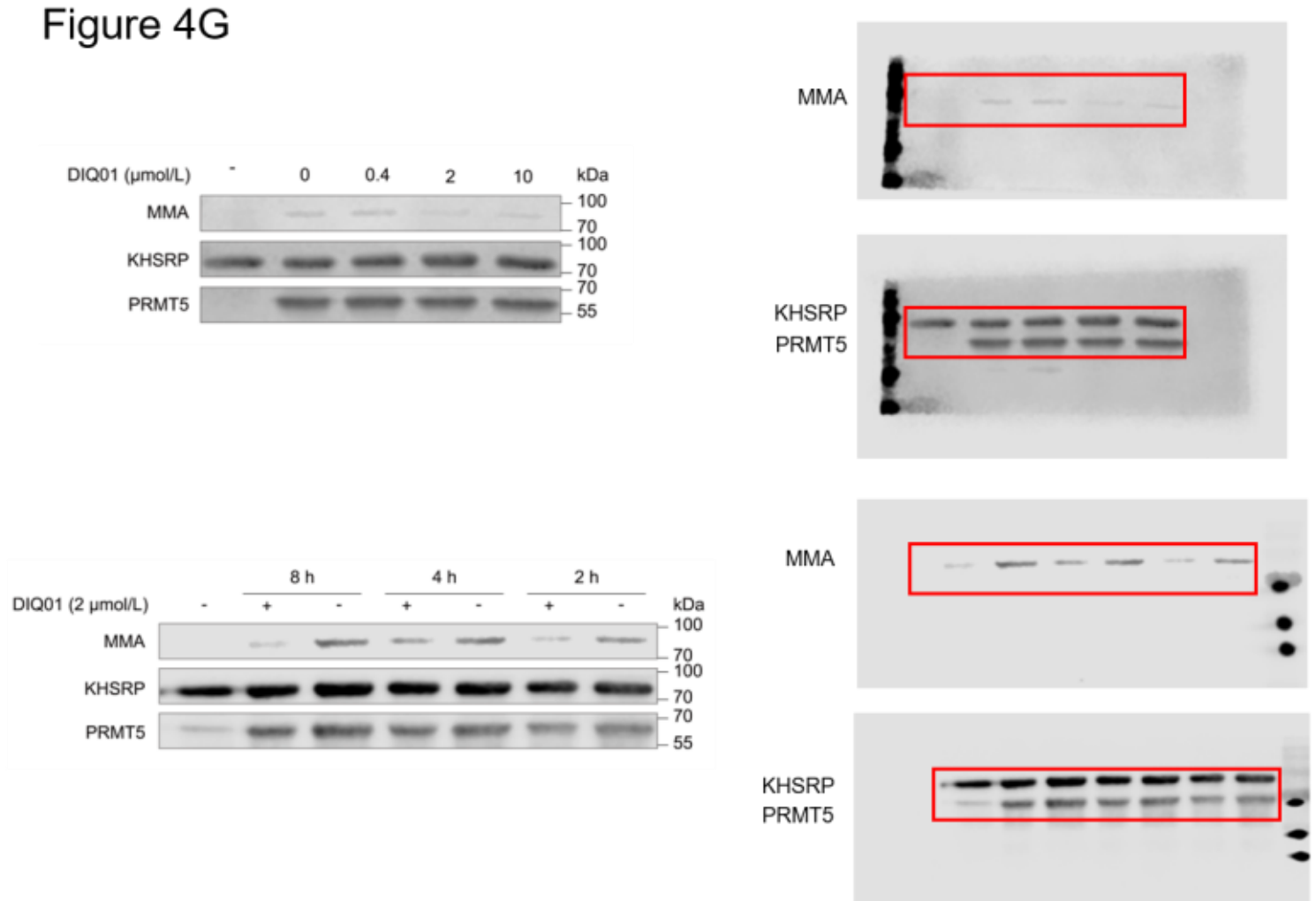

Figure 4H

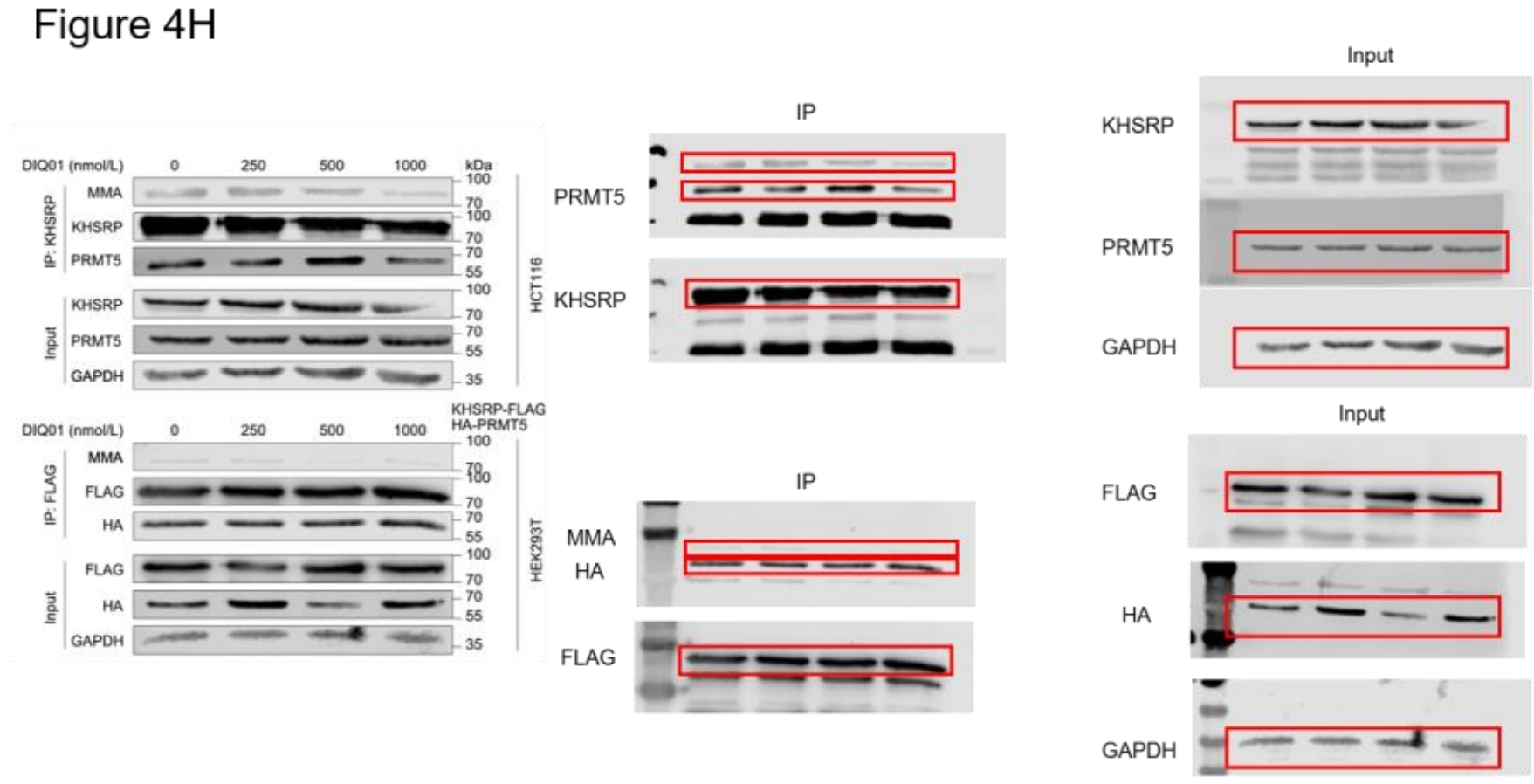

Figure 4I

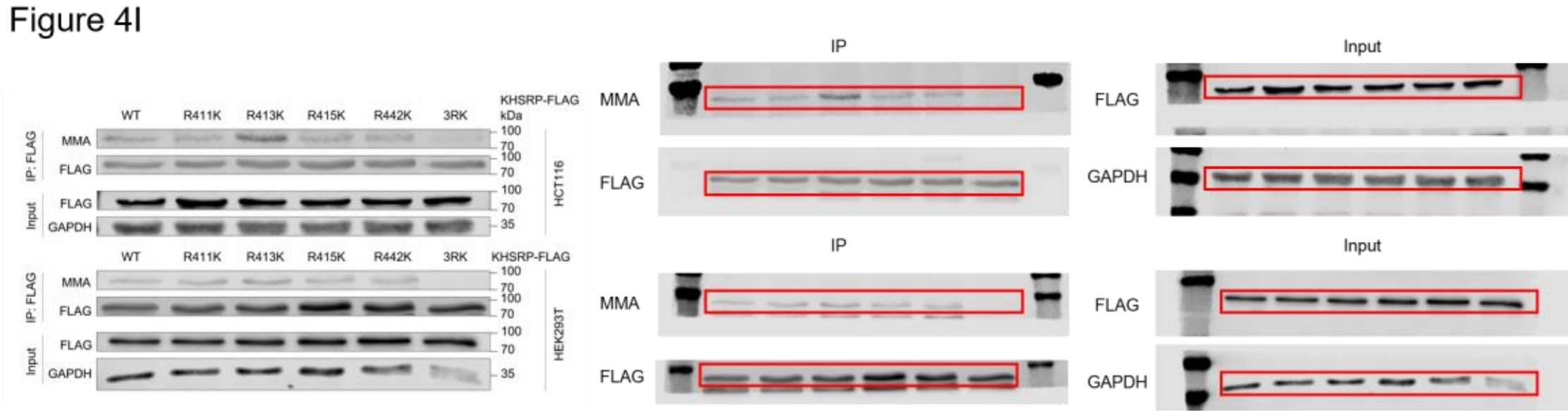

Figure 4J

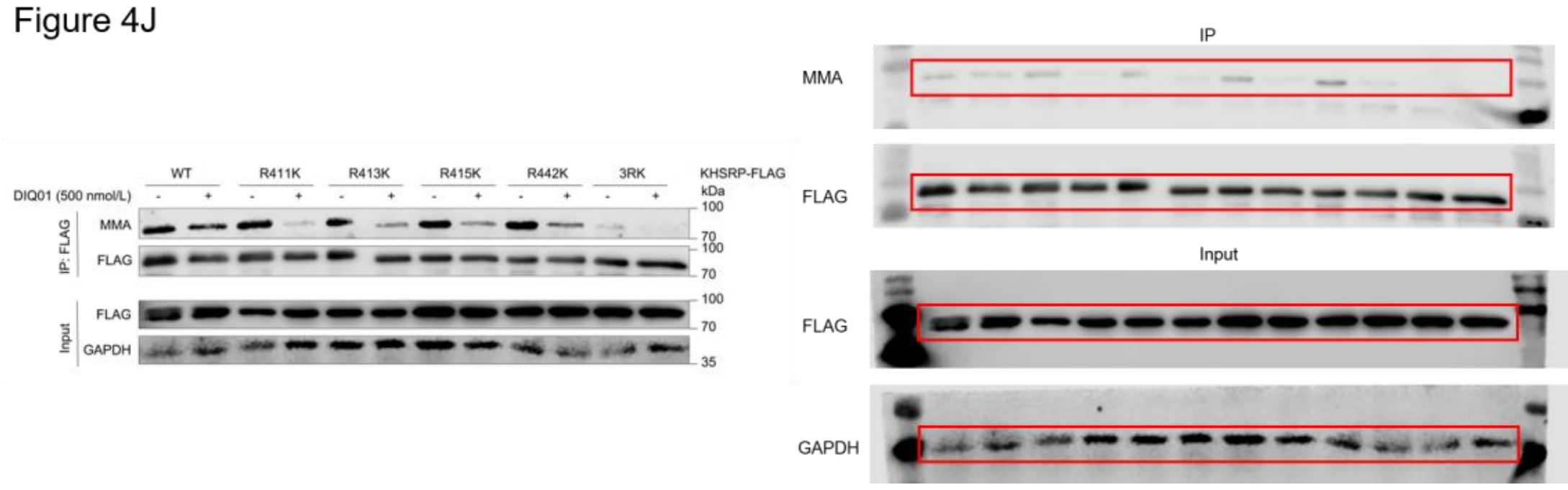

Figure 4L

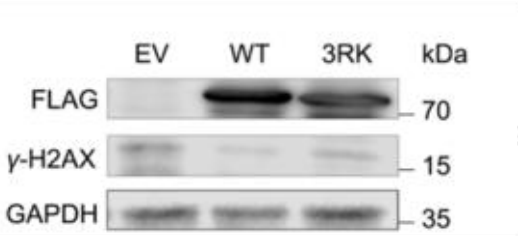

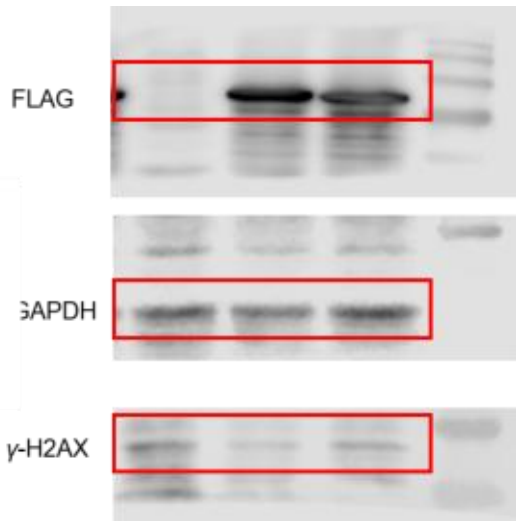

Figure S4A

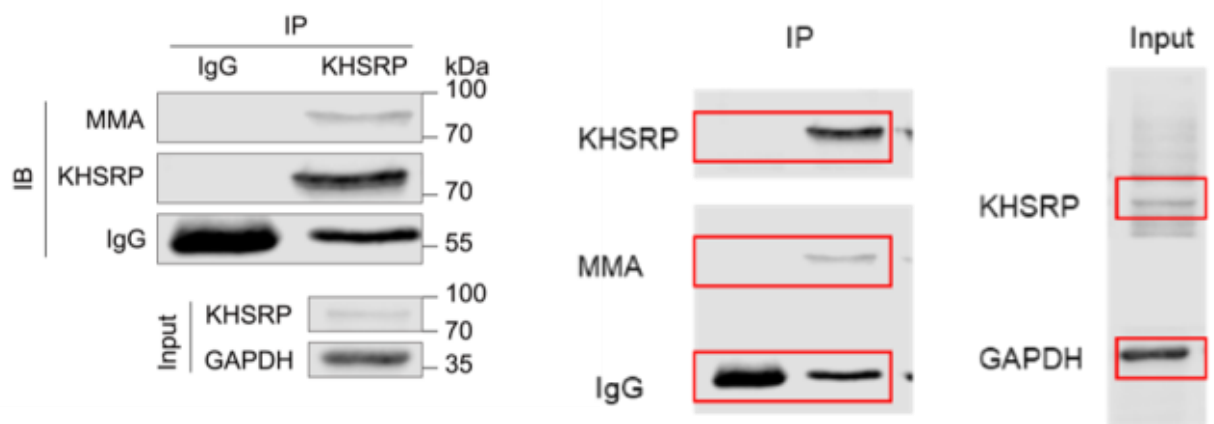

Figure S4B

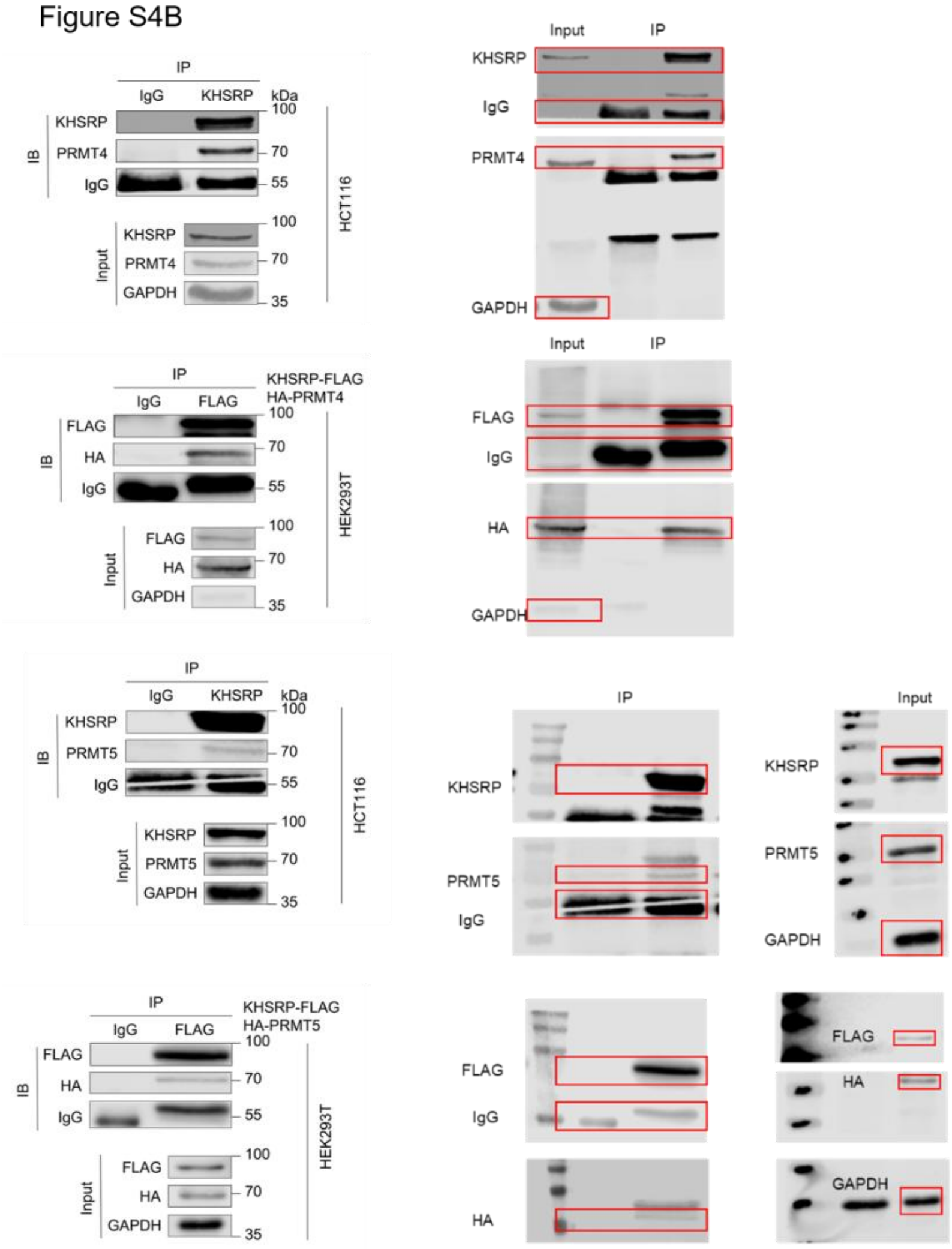
IP
IgG
KHSRP
kDa
100
70
55
IB
PRMT4
IgG
Input
GAPDH
35
HCT116
KHSRP-FLAG
HA-PRMT4
FLAG
HA
HEK293T
PRMT5
HA-PRMT5
Input
IP

Figure S4C

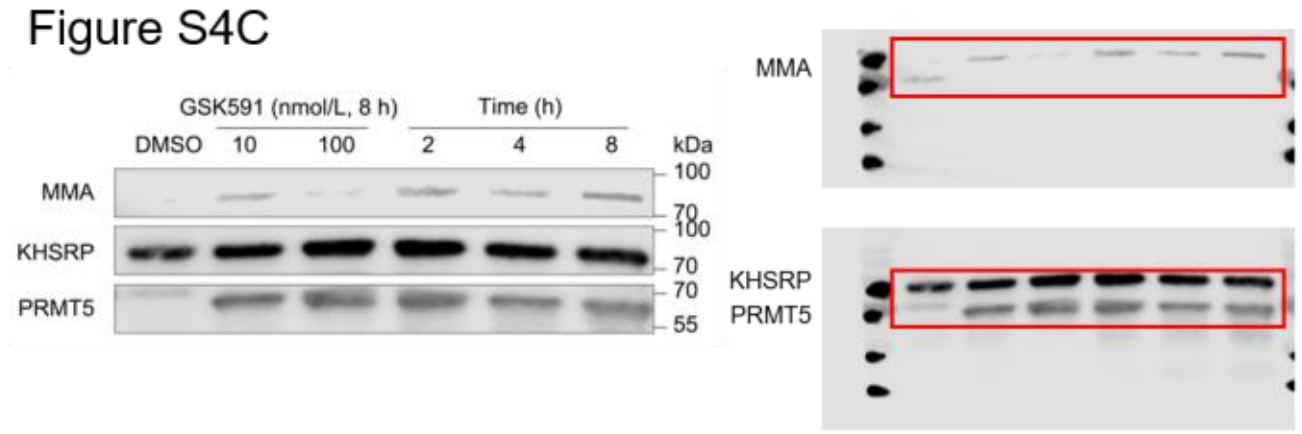

Figure S4D

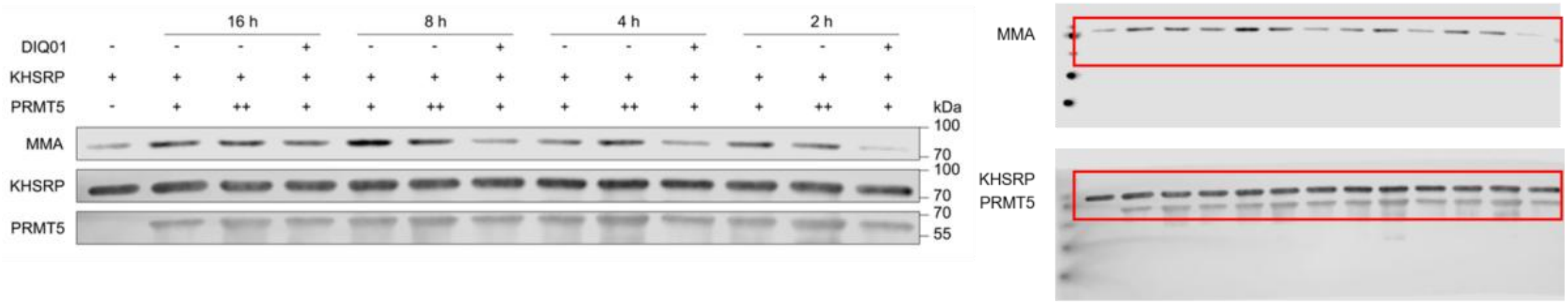

Figure S4E

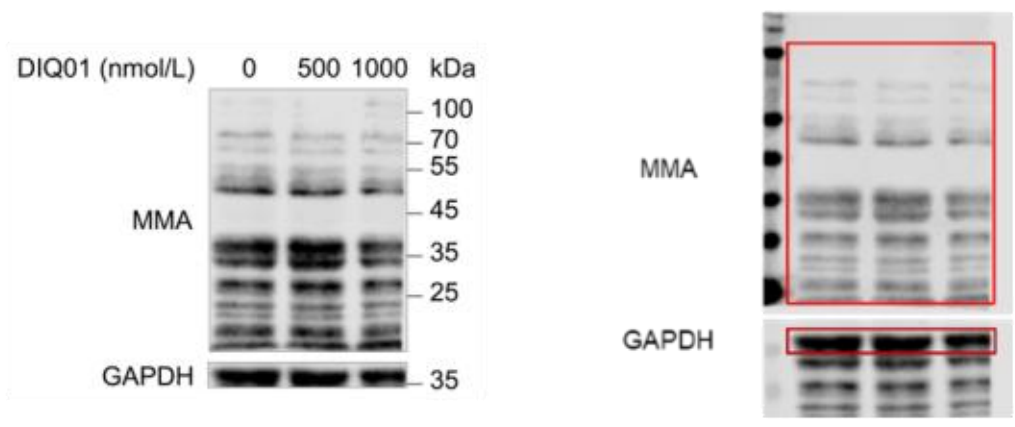

Figure S4G

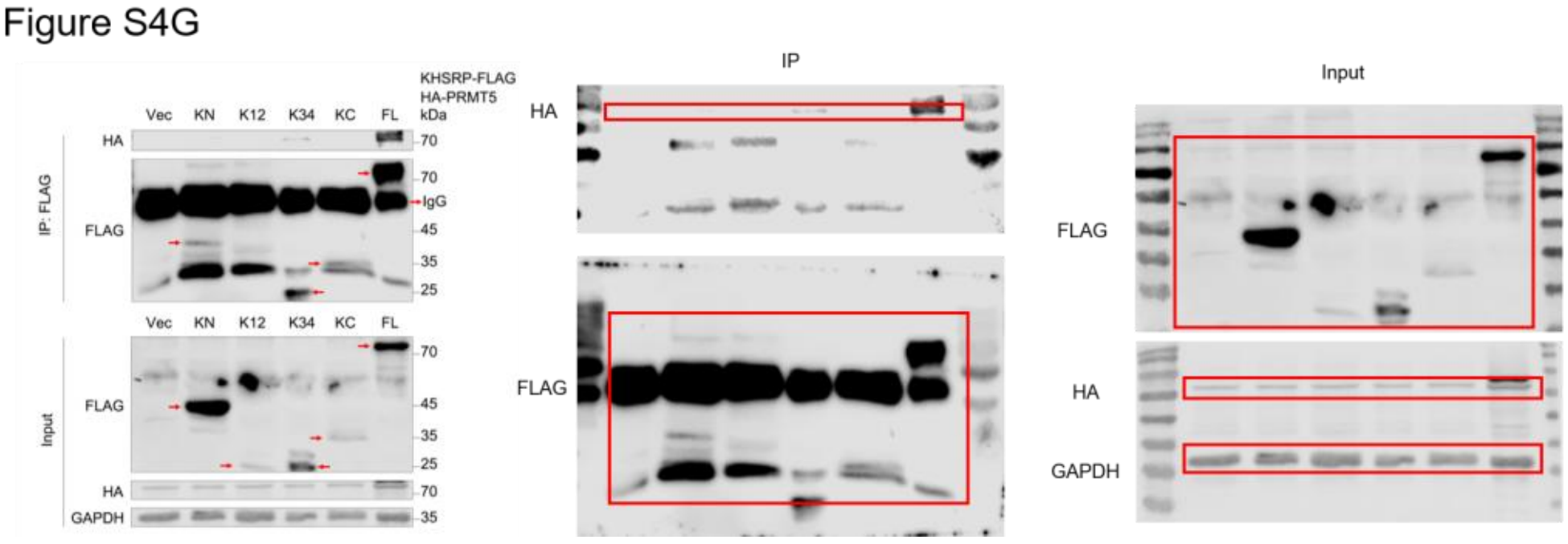

Figure S6B

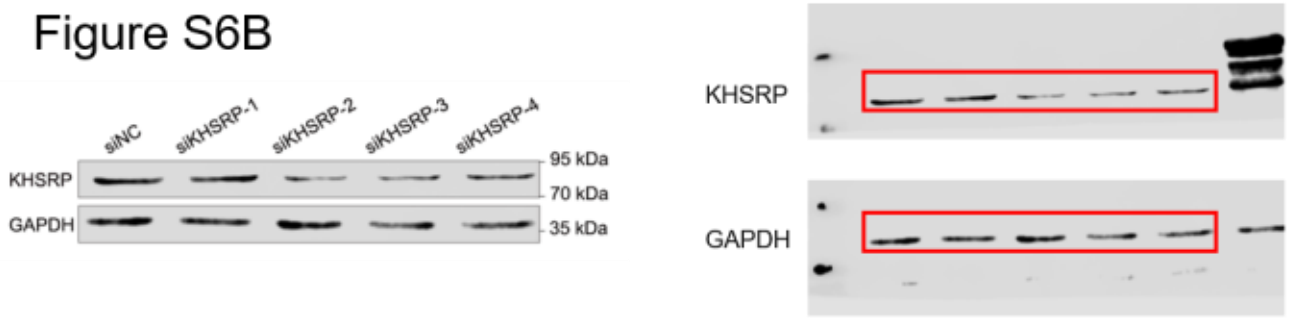

Figure S6C

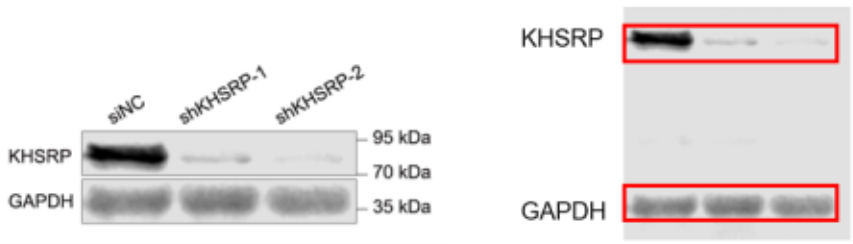

Figure S6D

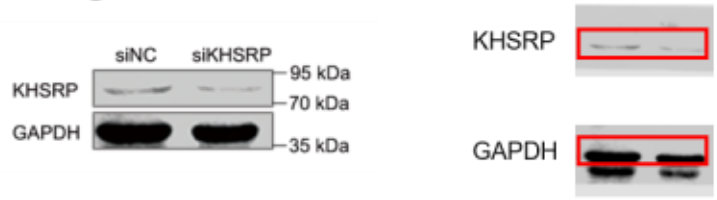

Figure S6E

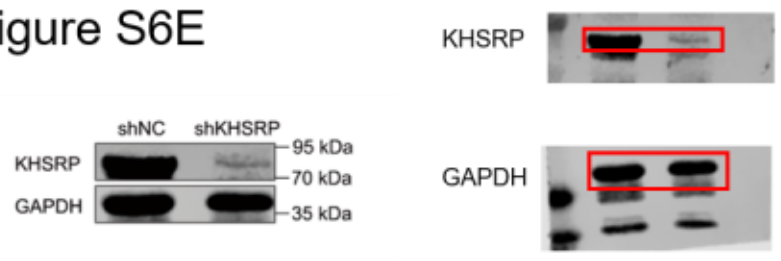

Figure S6F

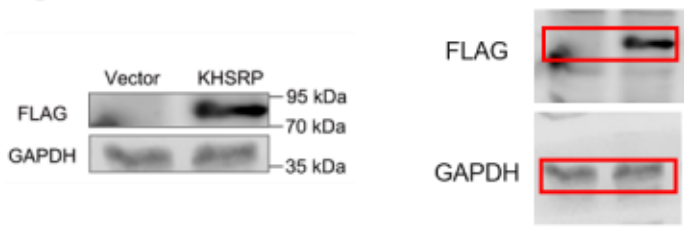

Figure S6H

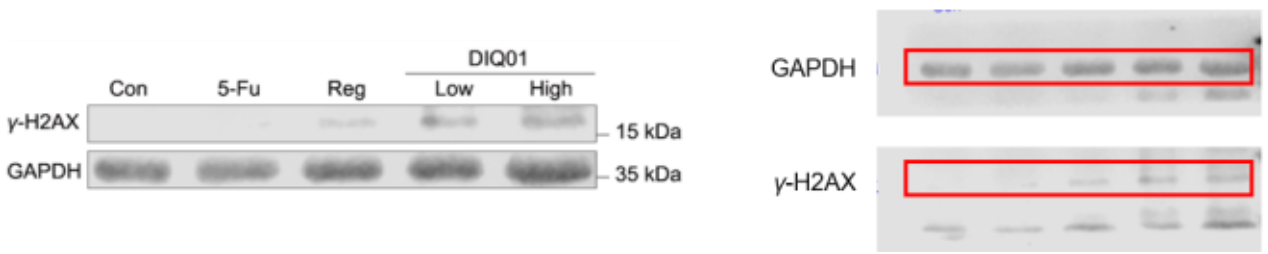

Figure 6H

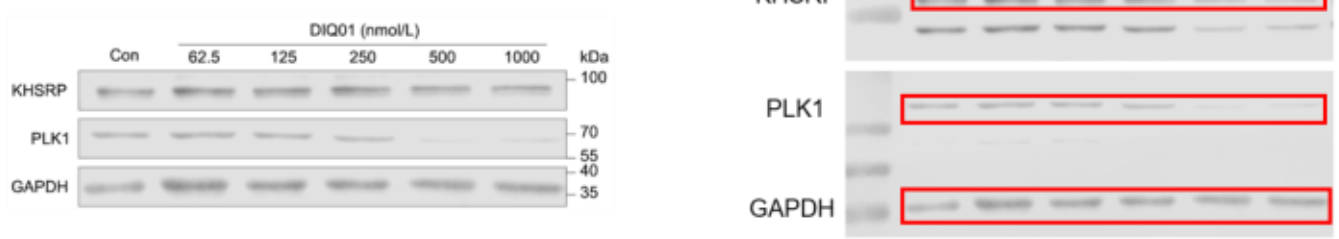

Figure 6I

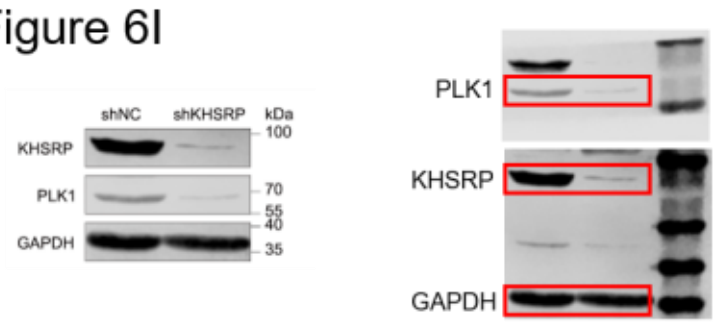

Figure 6K

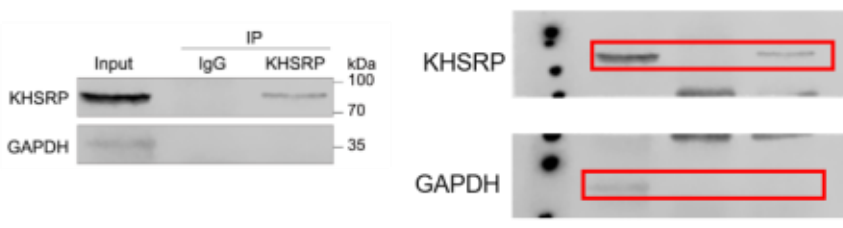

Figure 6J

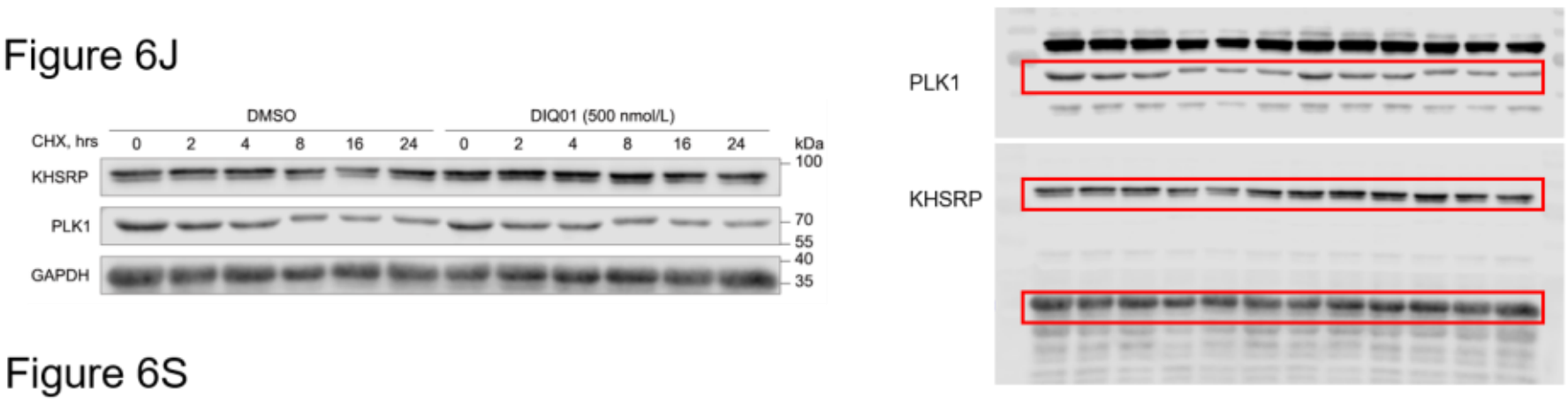

Figure 6S

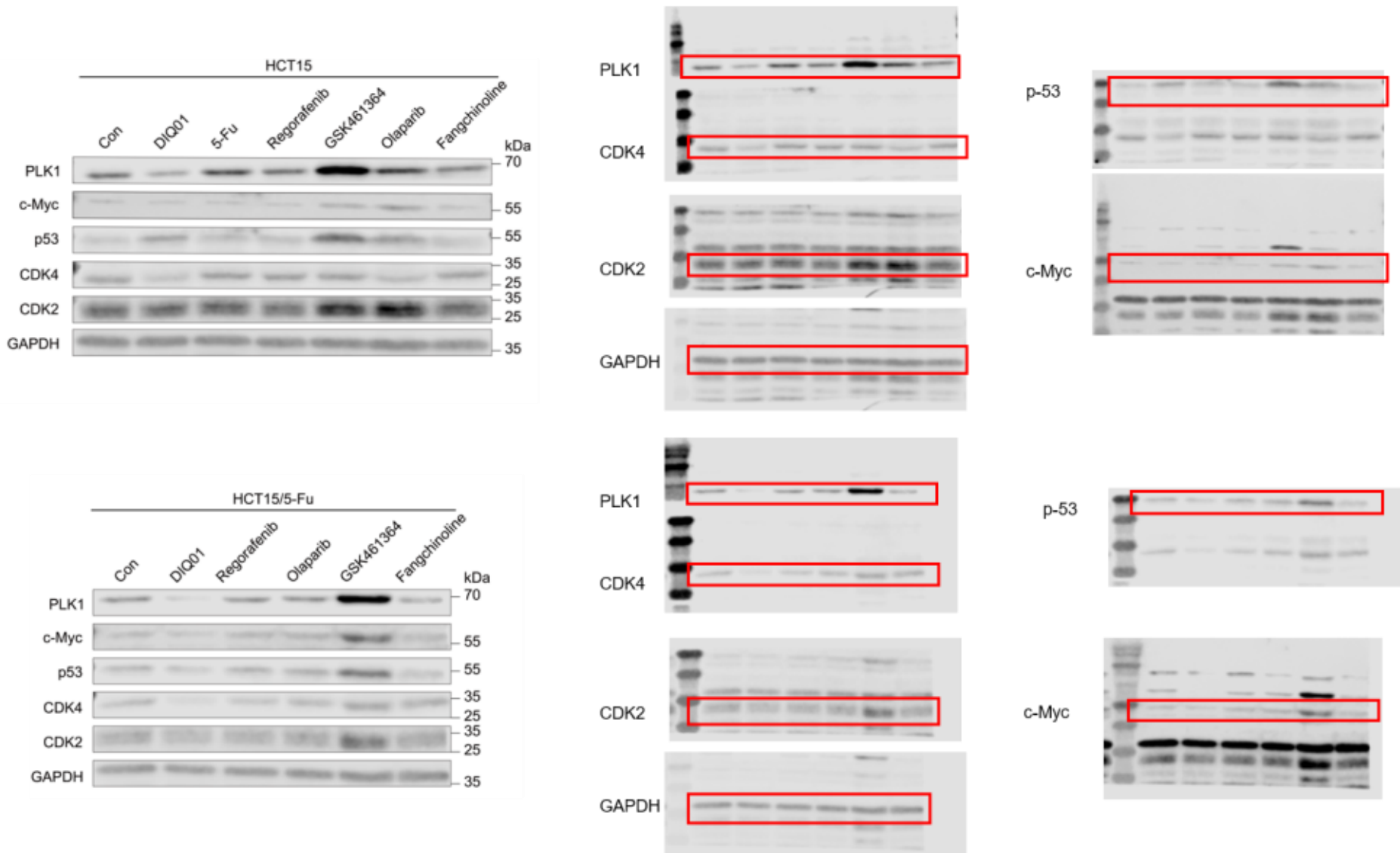

Figure 6T

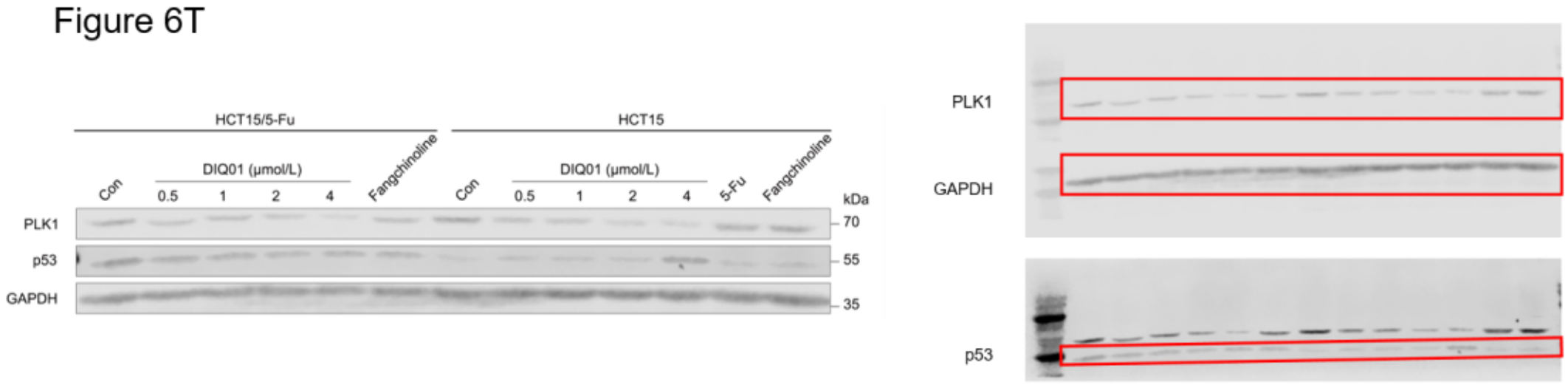

Figure S7G

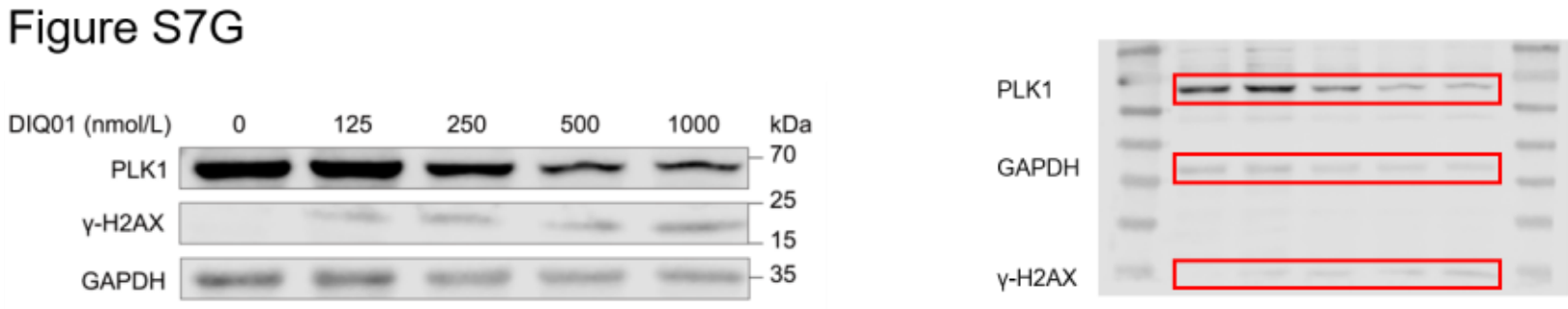

Figure S7H

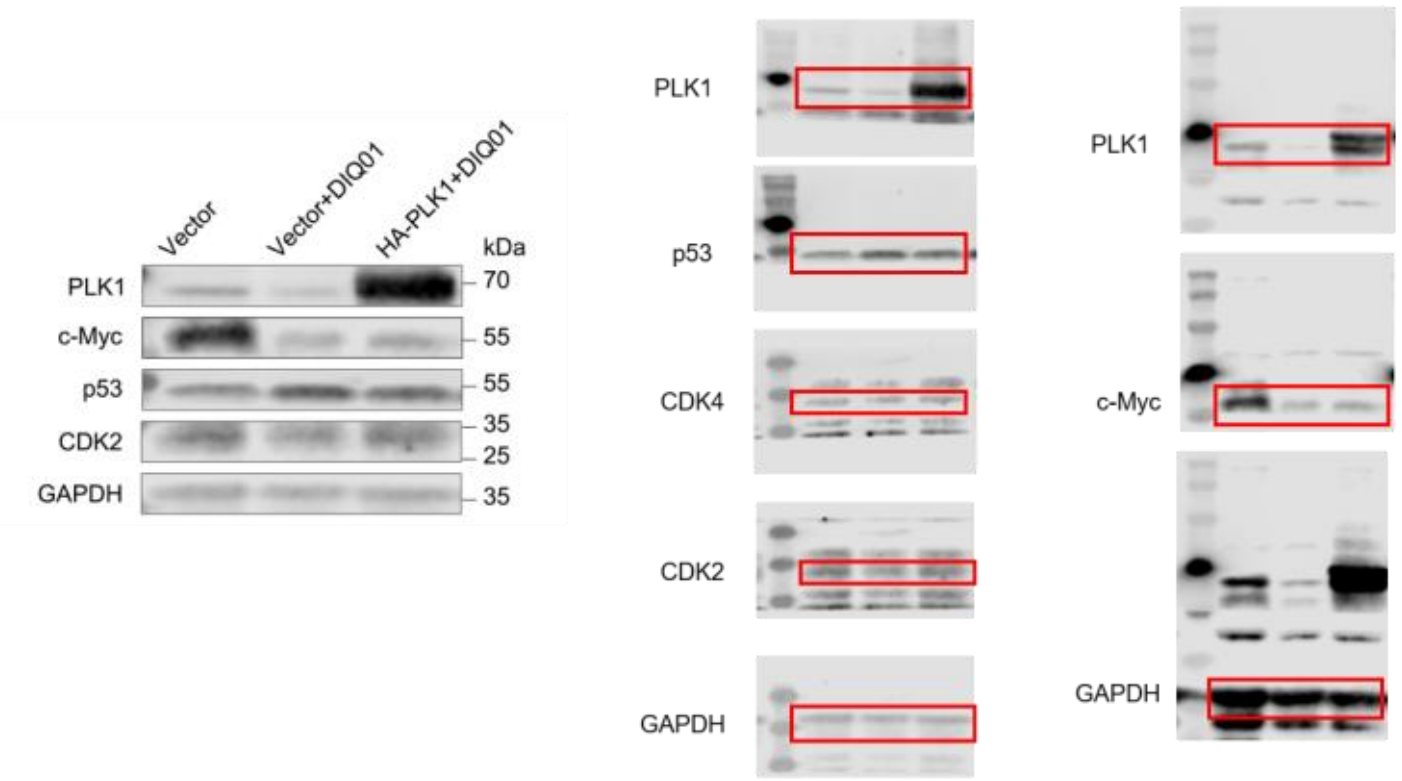

Figure S7I

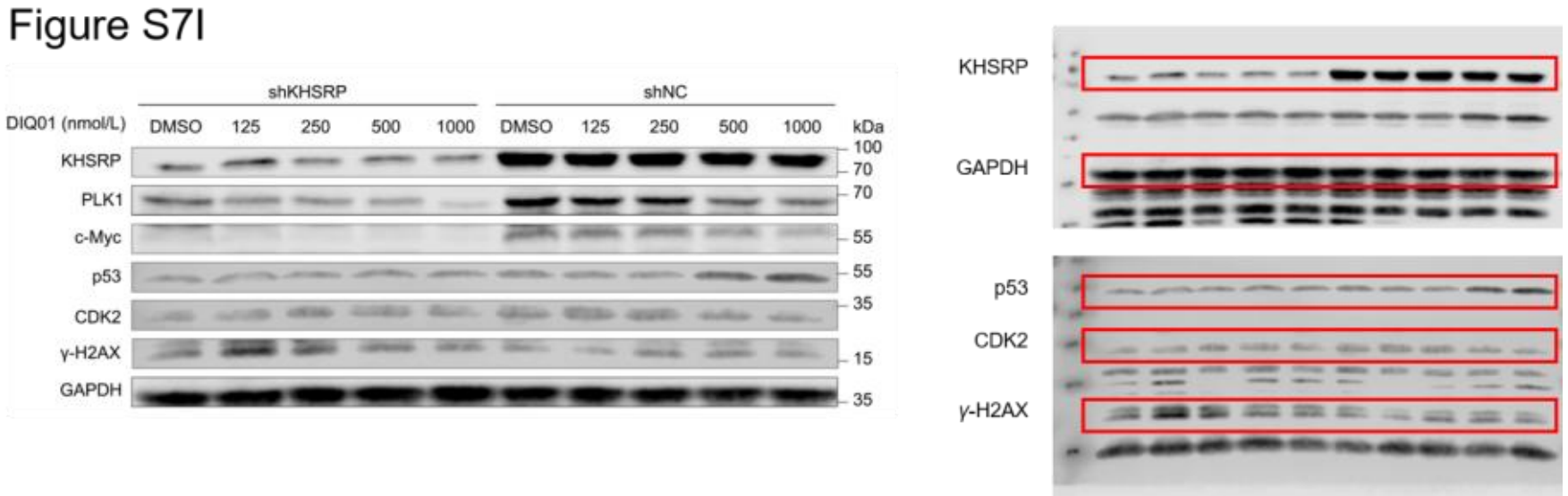

Figure S8G

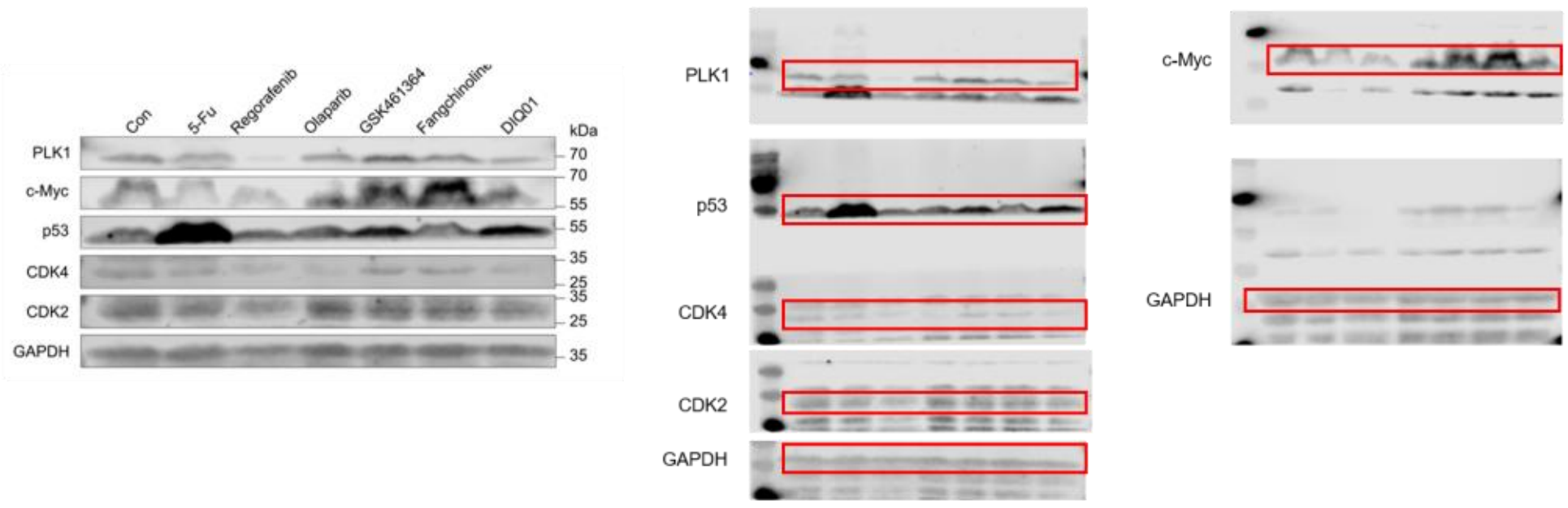

Figure S8H

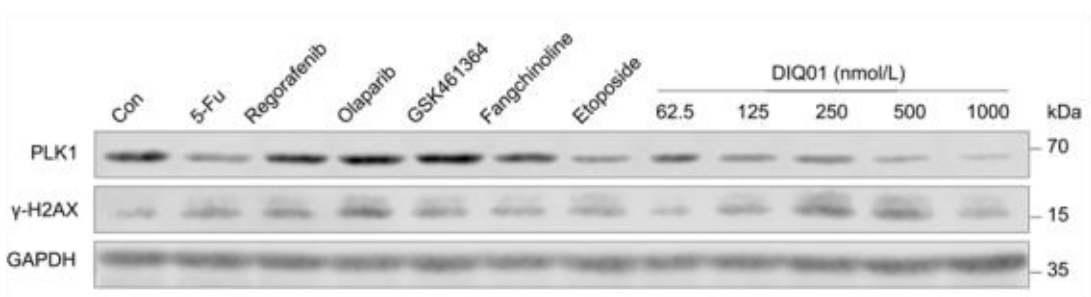

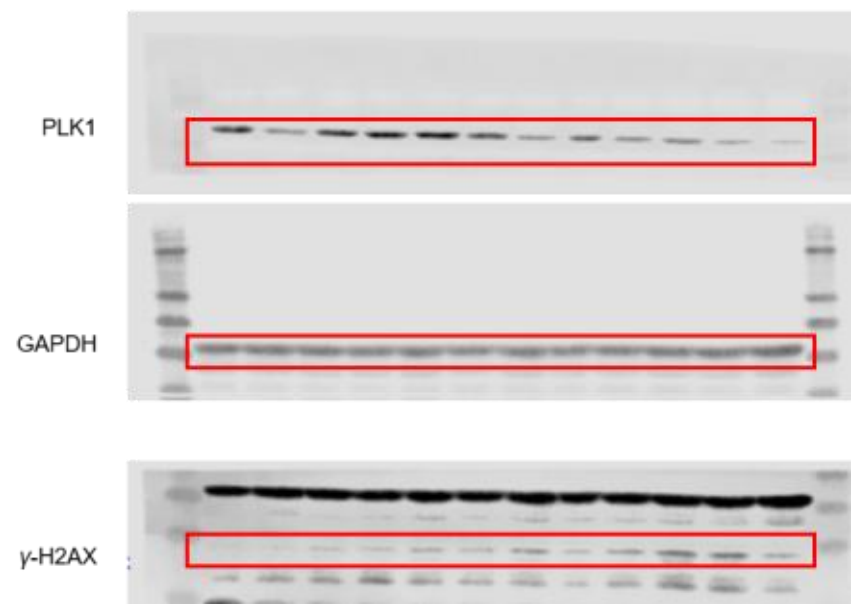

Supplement: Supplementary file 1 [file mmc1.pdf]
